# Manganese-Catalyzed Synthesis of Polyketones Using Hydrogen-Borrowing Approach

**DOI:** 10.1021/acscatal.4c03019

**Published:** 2024-06-28

**Authors:** Pavel S. Kulyabin, Oxana V. Magdysyuk, Aaron B. Naden, Daniel M. Dawson, Ketan Pancholi, Matthew Walker, Massimo Vassalli, Amit Kumar

**Affiliations:** † EaStCHEM, School of Chemistry, University of St Andrews, North Haugh, St Andrews KY16 9ST, U.K.; ‡ The Sir Ian Wood Building, Robert Gordon University, Garthdee Rd, Garthdee, Aberdeen AB10 7GE, U.K.; § Centre for the Cellular Microenvironment, Advanced Research Centre, University of Glasgow, Glasgow G116EW, U.K.; ∥ James Watt School of Engineering, University of Glasgow, Glasgow G12 8QQ, U.K.

**Keywords:** manganese, polyketones, dehydrogenation, diketone, hydrogen-borrowing

## Abstract

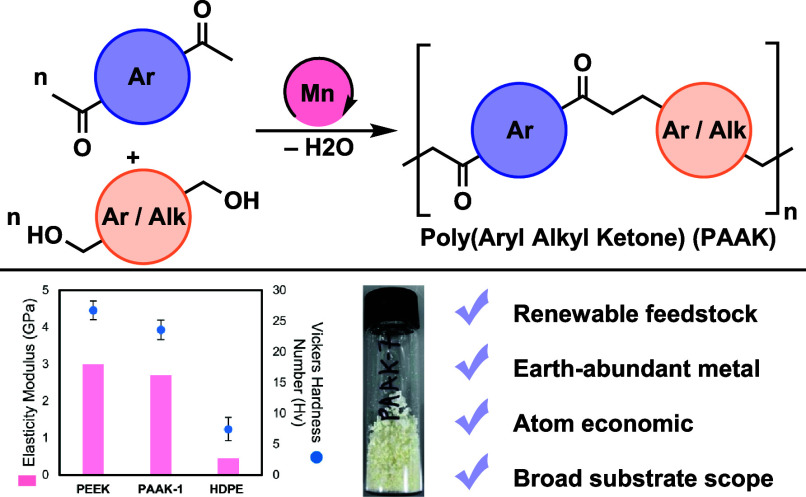

We report here a method of making polyketones from the coupling of diketones and diols using a manganese pincer complex. The methodology allows us to access various polyketones (polyarylalkylketone) containing aryl, alkyl, and ether functionalities, bridging the gap between the two classes of commercially available polyketones: aliphatic polyketones and polyaryletherketones. Using this methodology, 12 polyketones have been synthesized and characterized using various analytical techniques to understand their chemical, physical, morphological, and mechanical properties. Based on previous reports and our studies, we suggest that the polymerization occurs via a hydrogen-borrowing mechanism that involves the dehydrogenation of diols to dialdehyde followed by aldol condensation of dialdehyde with diketones to form chalcone derivatives and their subsequent hydrogenation to form polyarylalkylketones.

## Introduction

**Figure 1:**
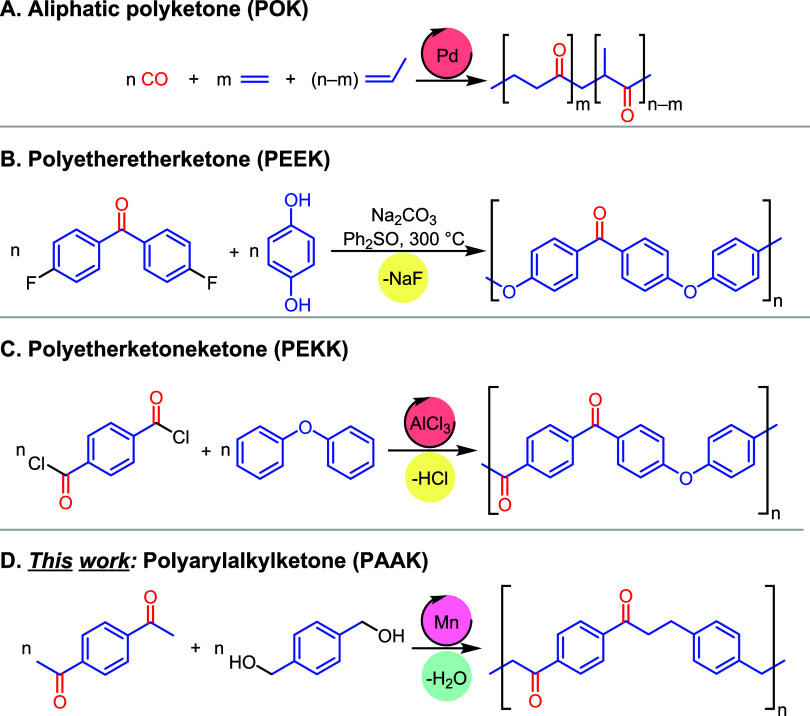
Methods for the synthesis of previously reported polyketones: aliphatic polyketone (POK), polyetheretherketone (PEEK), polyetherketoneketone (PEKK), and the polyketone reported herein: polyarylalkylketone (PAAK).

Polyketones are high-performance thermoplastics with a wide range of applications in the automotive, electronics, electrical, and medical industries.^1,2^ Compared with the structure of polyolefins, polyketones contain additional C=O groups in the polymer backbone chains which due to its polarity imparts excellent mechanical properties, crystallinity, hydrophilicity, and surface properties.^3^ Compared with polyamides, polyketones lack the NH group in the polymer backbone chain, which makes it much less hygroscopic and less sensitive to moisture. Despite the excellent properties of polyketones, this class of polymer has been relatively less studied. Aliphatic polyketones (POKs) are made from the reaction of late transition-metal-catalyzed coupling of ethene and/or propene with carbon monoxide (Figure 1A).^4^ Although the seminal reports on the synthesis of aliphatic polyketones (POK) date back to the 1940s and 1950s using nickel,^5,6^ and the 1980s using palladium,^7^ it was only in 1996 that POK was first commercialized by the Shell. However, the product was discontinued in 2000 due to reasons such as low demand and difficulty in polymer processing. Nevertheless, due to the demand of the POK, the product was relaunched in 2015 by Hyosung (a company in South Korea). Another class of polyketones is aromatic polyketones that also contain ether linkages and is known as polyaryletherketone (PAEK).^8^ The most common types of polymers from this class are polyetheretherketone (PEEK) and polyetherketoneketone (PEKK). These polymers have been commercialized since the 1980s and exhibit exceptional mechanical properties and chemical resistance. Their performance is considered the highest among all thermoplastics, and as a result, they are used in demanding applications such as aerospace, oil and gas drilling, and medical implants.^1,2^ Nevertheless, these polymers are difficult to process, and there is an ongoing need to develop new materials of this class bearing higher processability and keeping similar levels of thermal and mechanical properties. Additionally, in comparison to aliphatic polyketones (POK), aromatic polyketones or polyaryletherketones (PAEK) are around 10 times more expensive due to the use of more expensive feedstock/reagents. For example, polyetheretherketone (PEEK) is made from the nucleophilic substitution of 4,4′-difluorobenzophenone by the disodium salt of hydroquinone in the presence of a polar aprotic solvent such as diphenylsulfone at 300 °C (Figure 1B). Similarly, PEKK (polyetherketoneketone) is made via electrophilic polycondensation of diphenyl ether with mixtures of terephthaloyl chloride in the presence of AlCl_3_ catalyst (Figure 1C). Another drawback of these methodologies is limited substrate scope due to the lack of commercial or inexpensive functional monomers of this type. It is noteworthy that polyarylketones have been considered for several emerging applications in the recent past such as in the containment vessel for nuclear power plants,^9,10^ cryogenic hydrogen storage,^11^ and separators for batteries.^12^ Thus, there is a need to develop new methods to access diverse polyarylketones that could offer excellent thermal, physical, and mechanical properties and can be produced and processed economically.

It has been suggested in the past that the presence of alkyl chains in the aliphatic ketones provides the necessary flexibility for desirable processing whereas the presence of aromatic groups in polyaryletherketones (PAEK) provides the exceptional mechanical properties.^13^ Therefore, a polyketone containing both aryl and alkyl groups, polyarylalkylketone (PAAK) could potentially fill the gap between the properties of aliphatic and aromatic polyketones.

The concept of acceptorless dehydrogenative catalysis (where H_2_ gas is released as a byproduct) and borrowing hydrogen catalysis (where the released H_2_ is utilized to hydrogenate an intermediate in the reaction) are atom-economic approaches for the synthesis of organic compounds.^14^ The area has led to the discovery of several green transformations to make prevalent functional groups/compounds such as ketones,^15,16^ esters,^17^ amides,^18−20^ carboxylic acids,^21^ carbamates,^22,23^ ureas,^24−26^ amines,^27,28^ acetals,^29^ imines,^30,31^ and heterocycles.^32^ These strategies have also been utilized for the synthesis of polymers such as polyesters^33,34^ and polyamides,^34−36^ and more recently polyureas^37−39^ and polyethylenimines^40^ by us and others. Directly relevant to this report is the C-alkylation of ketones using alcohols that has been reported to undergo a borrowing hydrogen pathway by a number of transition metal catalysts such as ruthenium, manganese, and iron as recently reviewed by several groups.^41−47^ Despite several reports on this chemical transformation, the study has remained limited to the synthesis of small molecules. We envisioned that this strategy might allow us to make the hypothesized polyarylalkylketones (PAAK) from the metal-catalyzed coupling of diacetylaryls and diols for the first time. Since some diols can be prepared from renewable feedstocks, this approach can also allow us to make semirenewable aromatic polyketones for the first time.

## Results and Discussion

**Figure 2:**
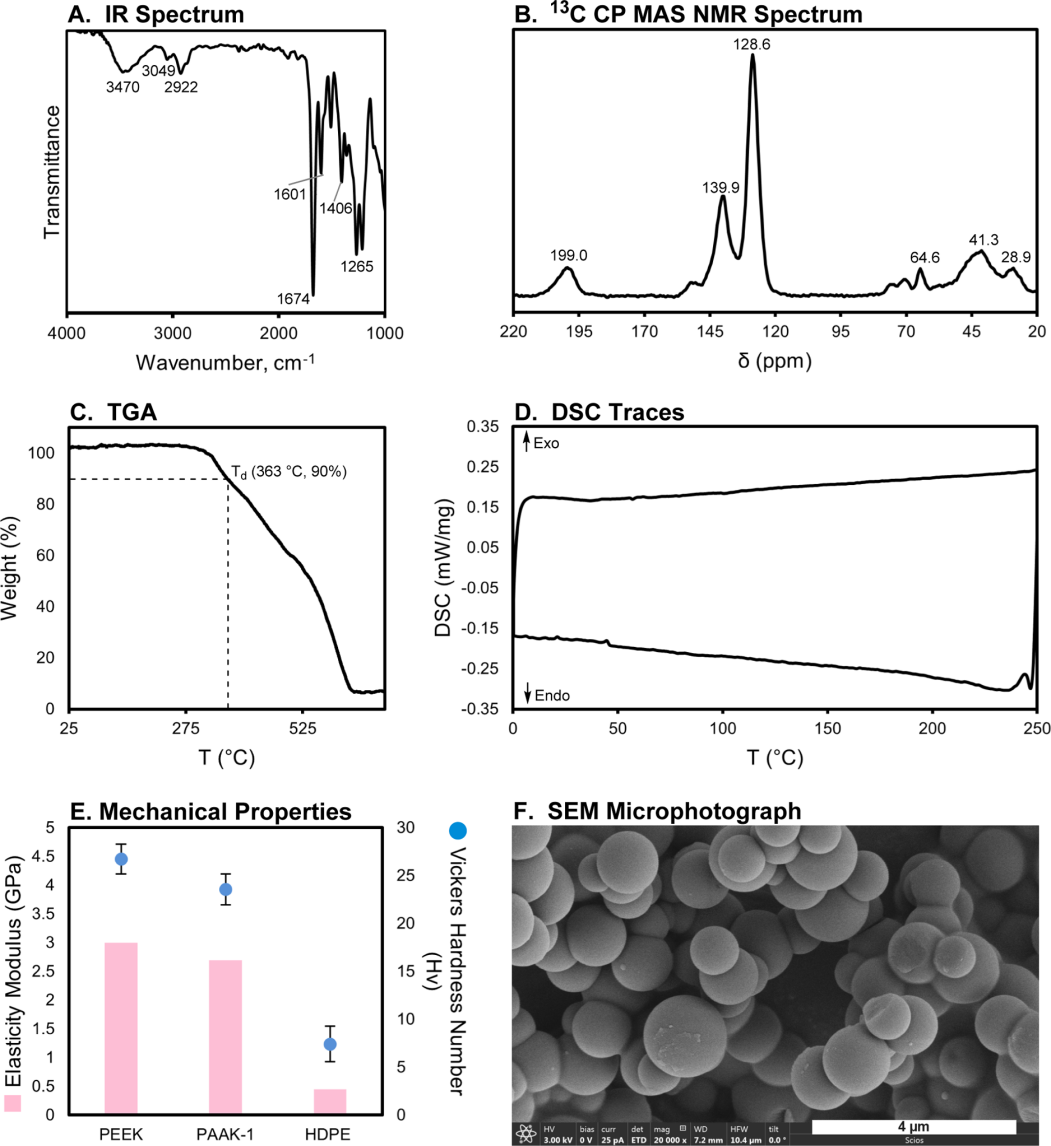
Charecterization of PAAK-1. (A) Infrared spectrum (ATR-FTIR). (B) ^13^C CP MAS NMR spectrum. (C) Mass loss as a function of temperature. (D) DSC plot. (E) Elasticity modulus and Vickers hardness number of commercial PEEK, polyketone PAAK-1, and HDPE. (F) SEM microphotograph.

**Scheme 1:**
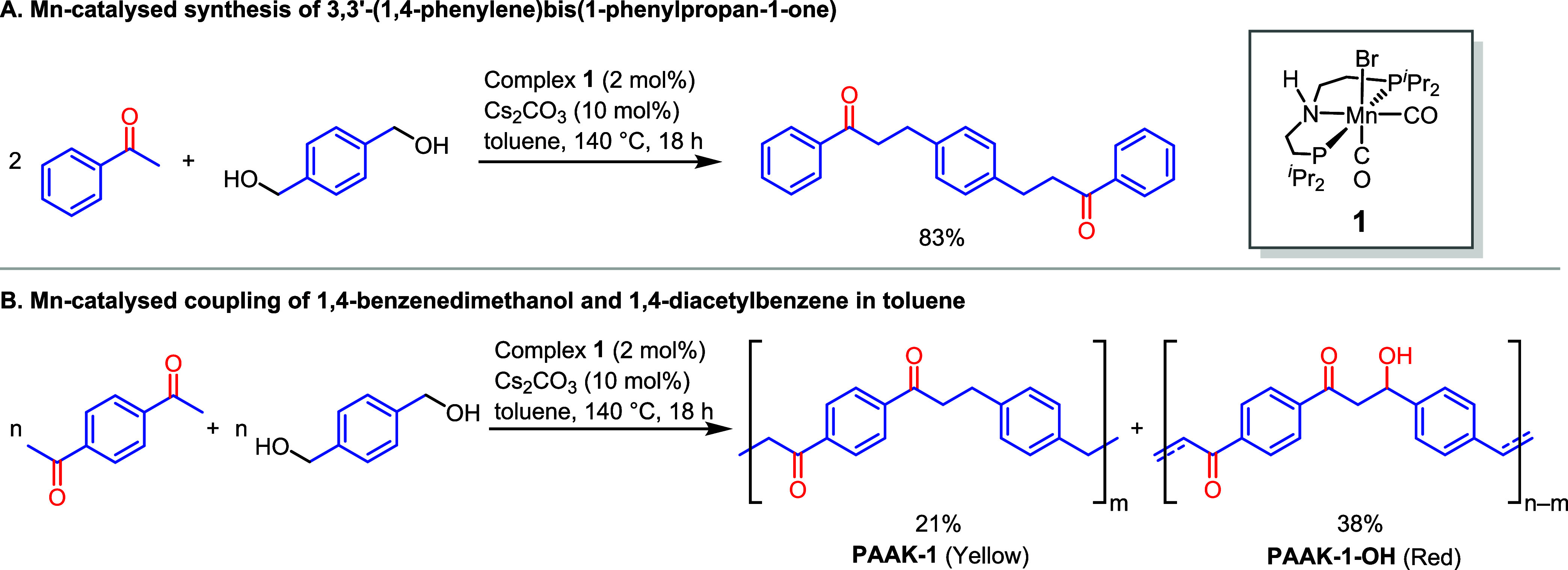
Coupling of Acetophenone (A) or 1,4-Diacetylbenzene (B) with 1,4-Benzenedimethanol in the Presence of the Precatalyst **1**

We started our investigation by studying a model reaction: coupling of acetophenone (0.4 M) with 1,4-benzenedimethanol (0.2 M) in the presence of 2 mol % complex **1** and 10 mol % Cs_2_CO_3_ in toluene (140 °C, 18 h). The choice of our initial catalytic conditions was inspired by the previous reports^41^ on the transition-metal-catalyzed C-alkylation of ketones using alcohols especially the one by Beller where reactions in toluene were as effective as that in 1,4-dioxane and *tert*-amyl alcohol.^48^ Remarkably, this led to the formation of the expected diketone in 83% isolated yield, which was characterized by NMR and IR spectroscopy (Scheme 1A). Motivated by this initial result, we studied the coupling of 1,4-diacetylbenzene (0.2 M) with 1,4-benzenedimethanol (0.2 M) under identical reaction conditions. The reaction led to the isolation of a mixture of yellow (21% yield) and red (38% yield) solids that could be physically separated (Scheme 1B). Both of these solids were found to be insoluble in common solvents such as toluene, DCM, acetone, chloroform, tetrahydrofuran (THF), chlorobenzene, water, dimethylformamide (DMF), dimethyl sulfoxide (DMSO), and trifluoroacetic acid, because of which we could not employ solution-state NMR spectroscopy to analyze the chemical structure of these materials. The IR spectrum of the yellow solid (Figure 2A) showed signals at 3049 and 2922 cm^–1^ corresponding to aromatic and aliphatic C–H stretching frequencies. The presence of aromatic rings was further confirmed by signals at 1601 and 1508 cm^–1^ characteristic of aromatic C=C stretches. A sharp signal at 1674 cm^–1^ characteristic of an aromatic C=O (ketone) stretching frequency was observed. A broad signal at 3470 cm^–1^ can be assigned to the O–H group, presumably the end group of the polymer. These spectral assignments are suggestive of the structure of the polymer to be PAAK-1 (Scheme 1B) and are also in agreement with a reported polyketone made from the reaction of styrene and CO that contained phenyl groups, CH_2_ linkages, and ketone groups.^49^ The IR spectrum of the red solid (Figure S5, see SI) looked very similar to that of the yellow solid except for the two distinctive signals at 1605 and 1373 cm^–1^ which are attributed to olefinic C=C and C–O bonds, respectively. Additionally, a much broader signal at 3348 cm^–1^ corresponding to the O–H stretch was also observed. Based on these observations, we suggest that the red solid is a polyarylalkylketone with some double bonds and O–H groups characterized to be PAAK-1-OH (Scheme 1B).

We hypothesized that the elimination of water from PAAK-1-OH might be facilitated by the presence of a proton source in the reaction mixture that would convert the hydroxy group into a better leaving group (water) or by using a polar protic solvent. Indeed, performing the reaction in *tert*-amyl alcohol (*t*AmOH) solvent resulted in the selective formation of PAAK-1 in 89% yield (Table 1, entry 1). Performing the same reaction in the presence of ruthenium^50,51^ and iridium^47^ complexes, **2**–**4** that have been previously reported for the catalytic dehydrogenative transformations led to relatively lower yields of PAAK-1 (Table 1, entries 2–4). We then studied the effect of various catalytic conditions, e.g., concentration of starting materials and base, and size of the reaction vessel, on the yield of the reaction. Interestingly, using 1 mol % complex **1** also led to the isolation of PAAK-1 in 87% yield (entry 5). Further reducing the catalytic loading to 0.5 mol % led to a lower but still very good, isolated yield (70%) of PAAK-1 (entry 6). Conducting a reaction at 0.1 M concentration of 1,4-diacetylbenzene and 1,4-benzene-dimethanol led to the formation of PAAK-1 in 85% yield which is similar to that conducted at 0.2 M concentration (entry 5) although a higher *T*_d_ (decomposition temperature, 373 °C, entry 7) was observed in the case of 0.1 M concentration in comparison to that of 0.2 M concentration (*T*_d_ = 356 °C, entry 5). Decreasing the concentration further to 0.05 M did not make any significant difference in the yield or *T*_d_ of the isolated polymer, in comparison to that of 0.1 M (entry 8). Changing the size of the reaction vessel from 100 to 15 mL did not make any significant difference in the yield and thermal stability of the polymer (entry 9). Increasing the amount of Cs_2_CO_3_ to 20 mol % (entry 10) led to a slight increment in yield (90%), whereas decreasing the amount of Cs_2_CO_3_ reduced the yield (80%, entry 11), suggesting the significance of base in the coupling process. Lowering the temperature to 110 °C reduced the yield to 67% (entry 12). Interestingly, studying the time profile of the reaction suggested that the reaction reaches completion in 2 h leading to 89% yield of the PAAK-1, whereas 81% yield is obtained in 1 h (entries 13, 14). Finally, when the reaction was conducted in the absence of complex **1** and by using just Cs_2_CO_3_ (10 mol %), 8% of the solid was isolated (entry 15). Based on the IR spectrum and thermal studies of the isolated material, we suggest that the obtained product is a polymer resulting from the self-condensation of 1,4-diacetylbenzene (see SI, Figures S44 and S45). At the same time, no conversion of any starting material was obtained when the reaction was conducted in the presence of complex **1** without using any base (entry 16). Another control experiment was carried out using the preactivated catalyst **1A**; however, although it resulted in the transfer hydrogenation of diketone through the dehydrogenation of diol, it did not result in the formation of the expected polyketone suggesting the significance of the role of base in polymer chain propagation (entries 17, 18). Additionally, conducting the reaction in the presence of Mn(CO)_5_Br (1 mol %) and the combination of Mn(CO)_5_Br (1 mol %) + PPh_3_ (3 mol %) resulted in only less than 5% yield of the polyketone material (entries 19 and 20) suggesting that the manganese-MACHO pincer complex (**1**) is important in the catalytic process. Thus, the optimized catalytic conditions are complex **1** (1 mol %), Cs_2_CO_3_ (10 mol %), 1,4-diacetylbenzene (0.1 M), 1,4-benzenedimethanol (0.1 M), 140 °C, 2 h, *t*AmOH (entry 13).

**Table 1:**
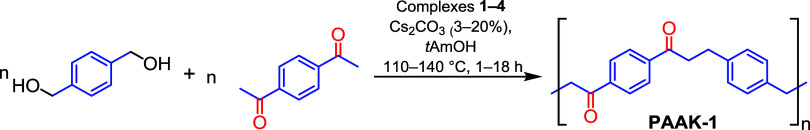
Optimization of Catalytic Conditions for the Coupling of 1,4-Diacetylbenzene and 1,4-Benzenedimethanol^a^

entry	complex	conc. (M)	Cs_2_CO_3_ (mol %)	time (h)	yield^b^ (%)	*T*_d_^c^, (°C)
1^d^	**1** (2 mol %)	0.2	10	18	89	338
2^d^	**2** (2 mol %)	0.2	10	18	<5	n.d.
3^d^	**3** (2 mol %)	0.2	10	18	51	353
4^d^	**4** (2 mol %)	0.2	10	18	17	319
5^d^	**1** (1 mol %)	0.2	10	18	87	356
6^d^	**1** (0.5 mol %)	0.2	10	18	70	348
7	**1** (1 mol %)	0.1	10	18	85	373
8^e^	**1** (1 mol %)	0.05	10	18	86	342
9^f^	**1** (1 mol %)	0.1	10	18	73	342
10	**1** (1 mol %)	0.1	20	18	90	369
11	**1** (1 mol %)	0.1	3	18	80	335
12^g^	**1** (1 mol %)	0.1	10	18	67	328
13	**1** (1 mol %)	0.1	10	2	89	363
14	**1** (1 mol %)	0.1	10	1	81	343
15	none	0.1	10	18	8	396
16	**1** (1 mol %)	0.1	none	18	none	n.d.
17^h^	**1A** (1 mol %)	0.1	1	18	none	n.d.
18^h^	**1A** (1 mol %)	0.1	2	18	<5	n.d.
19	Mn(CO)_5_Br (1 mol %)	0.1	10	18	<5	n.d.
20	Mn(CO)_5_Br (1 mol %)/PPh_3_ (3 mol %)	0.1	10	18	<5	n.d.

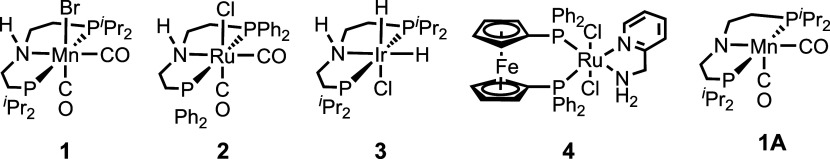

a General reaction conditions: 1,4-diacetylbenzene (0.5 mmol), 1,4-benzenedimethanol (0.5 mmol), 100 mL ampule with J-Young’s valve, temperature 140 °C, *t*AmOH.

b All yields are isolated yields.

c *T*_d_ stands for the decomposition temperature calculated from TGA (thermogravimetric analysis) as a temperature of 10% weight loss. N.d. stands for not detected.

d 1 mmol of 1,4-diacetylbenzene and 1,4-benzenedimethanol was used.

e 10 mL of *t*AmOH was used.

f Reaction in 15 mL pressure vessel.

g Reaction at 110 °C.*^h^* The activated complex **1A** was prepared with 1 or 2 equivalents of KO^t^Bu. See SI (Page S27–S28) for details.

h The activated complex **1A** was prepared with 1 or 2 equivalents of KO^t^Bu. See SI (Page S27–S28) for details.

The structure of PAAK-1 was further corroborated by a solid-state ^13^C{^1^H} CP MAS NMR spectrum that showed signals at δ 29–50, 128–151, and 199 ppm characteristic of alkyl, aryl, and ketone regions, respectively, confirming the structure of PAAK-1 (Figure 2B, corresponding to Table 1, entry 13). Additionally, analysis of the mother liquor upon precipitation of polymer in the case of Table 1, entry 6, by ^1^H and ^13^C{^1^H} NMR spectroscopy showed the presence of phenylcarbonyl, phenylenemethanol, acetophenonyl, and 1-phenyl-ethanol end groups and 1,3-diphenylpropanone fragments (see Section 1.4 in the SI). Further analysis of mother liquor by electrospray-ionization-mass spectrometry (ESI-MS) confirmed the presence of oligomers containing ketone and alcohol components (see section 1.13 in the SI). These intermediates support the structure of PAAK but it is possible that the polymer is irregular with randomly distributed keto and hydroxy groups and double bonds along the polymer chain.

Thermal properties of the polymer were investigated by thermogravimetric analysis (TGA) and differential scanning calorimetry (DSC), which revealed that the PAAK-1 is a thermoset material with a decomposition temperature of 363 °C (*T*_d_, 10% weight loss) as no melting temperature could be observed (Figure 2C,D). This was further confirmed by the powder X-ray diffraction (XRD) study that revealed that the polymer is amorphous in nature (Figure S39, SI). According to DSC analysis, the newly synthesized PAAK-1 does not have glass transition point (*T*_g_) (Figure S49, SI).^52^

To get some understanding of the mechanical properties of the synthesized polyketone (PAAK-1), we processed the polymer using hot compression to prepare a film of 2 mm thickness, which was used to study the load–displacement curve using nanoindentation. The elasticity modulus and Vickers hardness of the PAAK-1 were measured to be 2.7 GPa and 23.6 HV, respectively (Figure 2E). For comparison, the nanoindentation study was conducted with a commercial sample of PEEK and HDPE (high-density polyethylene) under identical conditions. Remarkably, the elasticity modulus and Vickers hardness number of the PAAK-1 were found to be comparable with the commercial sample of PEEK (3 GPa, and 26.7 HV), and higher than those measured for the HDPE (0.45 GPa and 7.4 HV). These numbers are also consistent with previous reports in the literature on the measurement of elasticity modulus and Vickers hardness of commercial PEEK and HDPE.^53,54^

The morphology of polymers plays important roles in polymer processing and their applications and spherical particles are desirable for various processing techniques such as selective laser sintering which is used for 3-D printing or additive manufacturing which can also be used to process polyketones.^55^ Polyketones can also be used in engineered powder, as was recently demonstrated by an electronics manufacturing company, Jabil Inc., which has launched PK5000 for additive manufacturing. This polyketone has desirable chemical and mechanical properties such as high impact strength and high abrasion resistance in comparison to nylons.^56,57^ A study of the morphology of PAAK-1 (made using Table 1, entry 9) using scanning electron microscopy (SEM) showed granular structures composed of small spherical particles of size around 1.3–1.5 μm (Figure 2F).

Having optimized the reaction conditions for the synthesis of PAAK-1 (polyarylalkylketone), we studied the substrate scope of our methodology to understand the structure–property relationships. As described in Table 2 (entry 2), the coupling of 1,4-diacetylbenzene and 1,3-benzenedimethanol led to the formation of the corresponding polyarylalkylketone in 77% yield. However, a lower yield of polyketone (41%) was obtained from the coupling of 1,4-diacetylbenzene and 1,4-cyclohexanedimethanol (entry 3). Remarkably, excellent yields of polyketones were obtained from the coupling of 1,3-diacetylbenzene with various diols (entries 4–6). To introduce ether functionality as in the case of polyaryletherketones (PAEKs), we used 4-acetylphenyl ether as a diketone feedstock. Remarkably, we were able to couple 4-acetylphenyl ether with various aromatic and aliphatic diols to make polyketones in moderate to excellent yields as described in Table 2, entries 7–12. Of particular significance is the use of *D*-Isosorbide as a diol (entry 12) that is a commercially available sugar derivative and can also be made from cellulose making the corresponding polyketone to be semirenewable.^58^ The polymers were characterized by IR spectroscopy and ^13^C CP/MAS solid-state NMR spectroscopy that showed signals corresponding to C=O, aromatic, and aliphatic groups (see SI, Section 1.5). Polyketone reported in entry 7, Table 2 (PAAK-7) was additionally analyzed with TGA-MS that showed the way this material decomposes. It was found that PAAK-7 starts decomposing at 320 °C with elimination of diketone-like components (*m*/*z* = 239 and 254 g/mol, Figure S151, SI), and xylene-derived components (*m*/*z* = 79, 91, 92, and 107 g/mol) become the major components of ion current at temperatures higher than 400 °C (Figure S150, SI). It confirms that both components of the reaction mixture are incorporated into the final product.

**Table 2:**
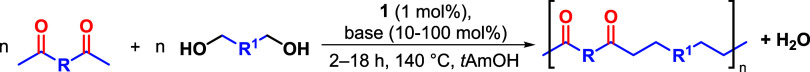

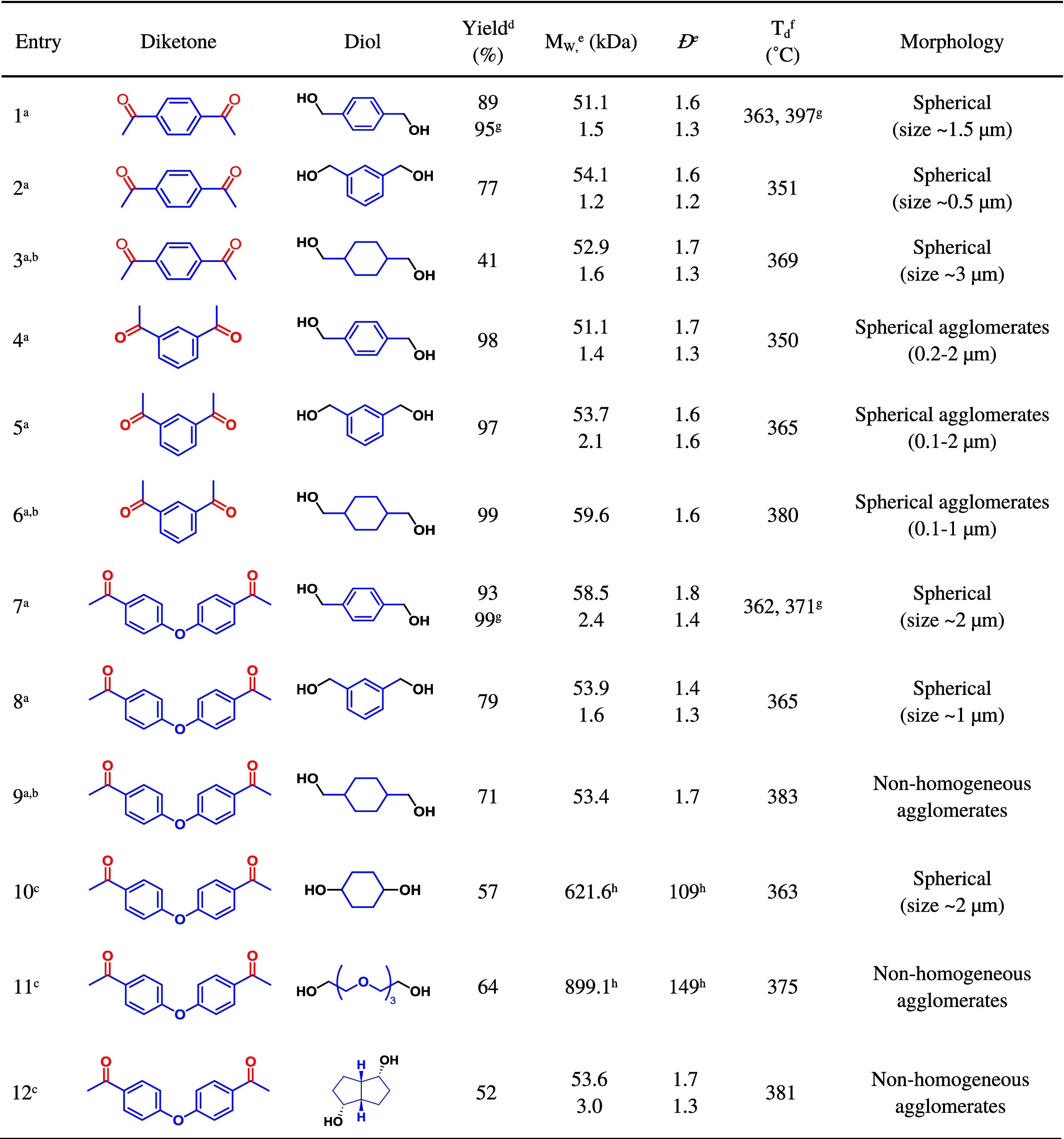
Substrate Scope for the Synthesis of Polyketones from Diketones and Diols

a Reaction conditions: diketone (0.5 mmol), diol (0.5 mmol), 1 (2.5 mg, 0.005 mmol), Cs_2_CO_3_ (16.5 mg, 0.05 mmol) in a 100 mL J-Young’s flask, temperature 140 °C, 2 h. All yields are isolated yields.

b 18 h.

c *t*BuOK (56 mg, 0.5 mmol), 18 h.

d All yields are isolated yields.

e Polymer samples were heated in Cl_2_CHCOOH at 120 °C overnight, filtrated, diluted with CHCl_3_, and then GPC analysis was performed in CHCl_3_/Cl_2_CHCOOH = 8/2 mixture at 35 °C.

f *T*_d_ corresponds to the temperature of 10% weight loss.

g Reaction is conducted under H_2_ atmosphere.

h GPC showed polymodal distribution.

As mentioned earlier, the isolated materials showed no solubility in all of the attempted solvents (toluene, water, methanol, THF, CHCl_3_, DCM, DMF, DMSO, TFA, and HFIP) at room temperature or on heating (100 °C) which made analysis of the polymers by gel permeation chromatography (GPC) very challenging. However, we managed to partially dissolve the material in dichloroacetic acid (upon heating overnight at 120 °C) and the soluble part was analyzed by GPC in dichloroacetic acid/CHCl_3_ solvent mixture. Most of the dissolved polymers showed bimodal distribution with a low-molecular-weight component of 1.2–3.0 kDa (*Đ* = 1.2–1.6) and a high-molecular-weight component of 51.1–58.5 kDa (*Đ* = 1.6–1.8) (Table 2). Extremely broad polydispersities were observed for polymers reported in cases of entries 10 and 11, Table 2. Interestingly, monomodal distribution was obtained in cases of entries 6 and 9 (Table 2) when 1,4-cyclohexyldimethanol was utilized as a diol with *M*_w_ = 59.6 kDa (*Đ* = 1.6) and 53.4 kDa (*Đ* = 1.7), respectively. The same method was used to analyze molecular masses of commercial polyketones (Table S3, SI), POK and PEKK being completely soluble in dichloroacetic acid and PEEK only partially soluble. GPC showed that all of these polymers are monomodal (*Đ* = 2.2–7.5) and have a higher molecular weight (63.3–126.8 kDa) than PAAK polymers reported herein.

The decomposition temperature (*T*_d_), calculated as a temperature of 10% weight loss from TGA (thermogravimetric analysis), was found to be in the range of 321–383 °C as described in Table 2. This was lower than what was found for commercial PEEK and PEKK samples (*T*_d_ = 581 and 557 °C, respectively, see SI, Table S3) and close to the thermostability of commercial POK (*T*_d_ = 387 °C, see SI, Table S3). The powder XRD studies showed that all of the polyketones reported herein are amorphous in nature with some polymers containing an unidentified component of crystallinity (see SI, Section 1.5 for full details). This is consistent with the absence of any melting temperature in DSC traces of these polyketones. Additionally, DSC traces of PAAKs do not demonstrate any glass transition temperature which could be a sign of cross-linking in the material. It is known that a certain degree of cross-linking can increase the glass transition temperature of the polymer above the decomposition temperature.^52^ In support of this, the traces of terephthalic aldehyde-derived cross-linking were observed in the high-resolution mass spectrum of crude reaction mixture of small-molecule model reaction shown in Scheme 1A (Figure S1, SI). Presumably, since the glass transition temperature of aromatic polyketones is usually higher than 100 °C, even one cross-link between two polymer chains could be enough to increase *T*_g_ above the decomposition temperature. The morphology of polyketones for most cases showed agglomerates of spherical particles in the range of 0.2–3 μm, as described in Table 2. In some cases, the particle sizes were more uniform (e.g., entry 1) than others (e.g., entry 4), whereas in some cases, nonhomogeneous agglomerates were observed (see SI, Section 1.18).

We envisioned that since the product precipitates out from the reaction medium whereas the catalyst is likely to remain soluble, this presents an opportunity to test the recyclability of the catalyst. After the reaction conducted as described in Table 2, entry 7 (coupling of 4-acetylphenyl ether (0.5 mmol) and 1,4-benzenedimethanol (0.5 mmol) that led to the isolation of polyketone in 89% yield), the mother liquor solution was transferred to another Young’s flask containing 4-acetylphenyl ether (0.5 mmol), 1,4-benzenedimethanol (0.5 mmol), and Cs_2_CO_3_ (10 mol %). The reaction mixture was then refluxed at 140 °C for 2 h, resulting in the isolation of PAAK-7 in 55% yield showing an IR spectrum identical to that of the polymer isolated in the first batch (see Section 1.9 in the SI). Interestingly, when the recycling study was performed without adding base in the second stage, no precipitate was observed, suggesting the involvement of base in steps other than generating the active species from the precatalyst **1**.

**Scheme 2:**
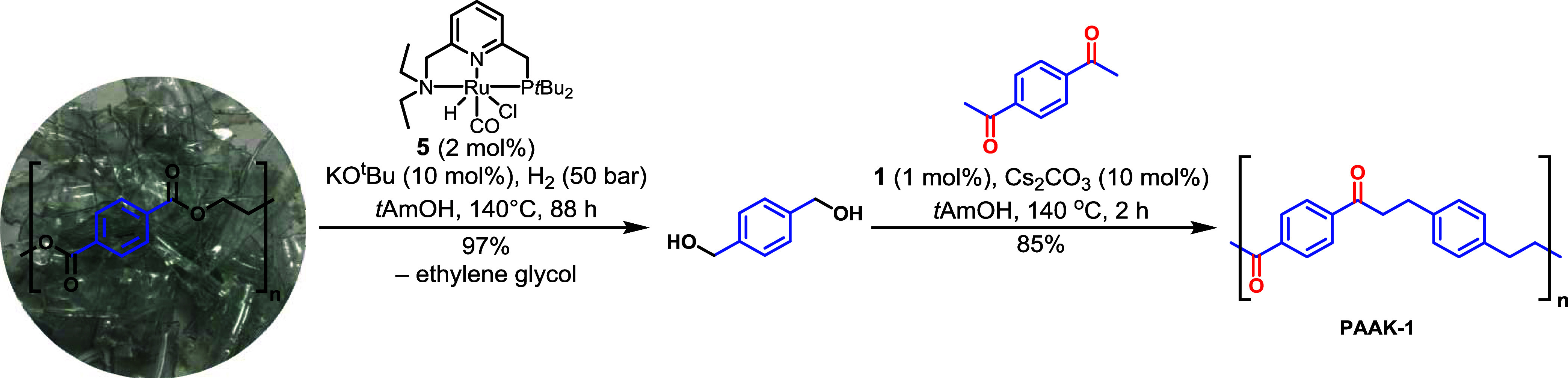
Synthesis of Polyketone PAAK-1 from 1,4-Benzenedimethanol Derived from the Waste Plastic Bottle

In pursuit of methods to make (semi)renewable plastics, we envisioned if a polyketone could be made from a diol sourced from the depolymerization of waste plastic such as poly(ethylene terephthalate) (PET). To achieve this, we carried out a two-step process where PET waste (sourced from plastic bottle) was first hydrogenatively depolymerized in a pressure reactor using the Milstein’s ruthenium PNN catalyst (**5**, 2 mol %, and KO*t*Bu, 10 mol %) in *t*AmOH solvent to form 1,4-benzenedimethanol and ethylene glycol in approximately quantitative yields as confirmed by the ^1^H NMR spectroscopy (see Section 1.11 in SI). Analogous reaction on the hydrogenative depolymerization of PET has been reported previously by Robertson^59^ and Klankermayer.^60^ The mixture of 1,4-benzenedimethanol and *t*AmOH was then separated from ethylene glycol by extraction in DCM/water to which 1,4-diacetylbenzene, manganese complex **1** (1 mol %), and Cs_2_CO_3_ (10 mol %) were added and the reaction mixture was heated for 2 h at 140 °C as described in Table 2. This led to the isolation of PAAK-1 in 85% yield (Scheme 2). The reaction in the case when 1,4-benzenedimethanol was not separated from ethylene glycol led to the formation of a polyketone in only 21% yield that contained hydroxyl groups and double bonds according to IR spectroscopy.

**Scheme 3:**
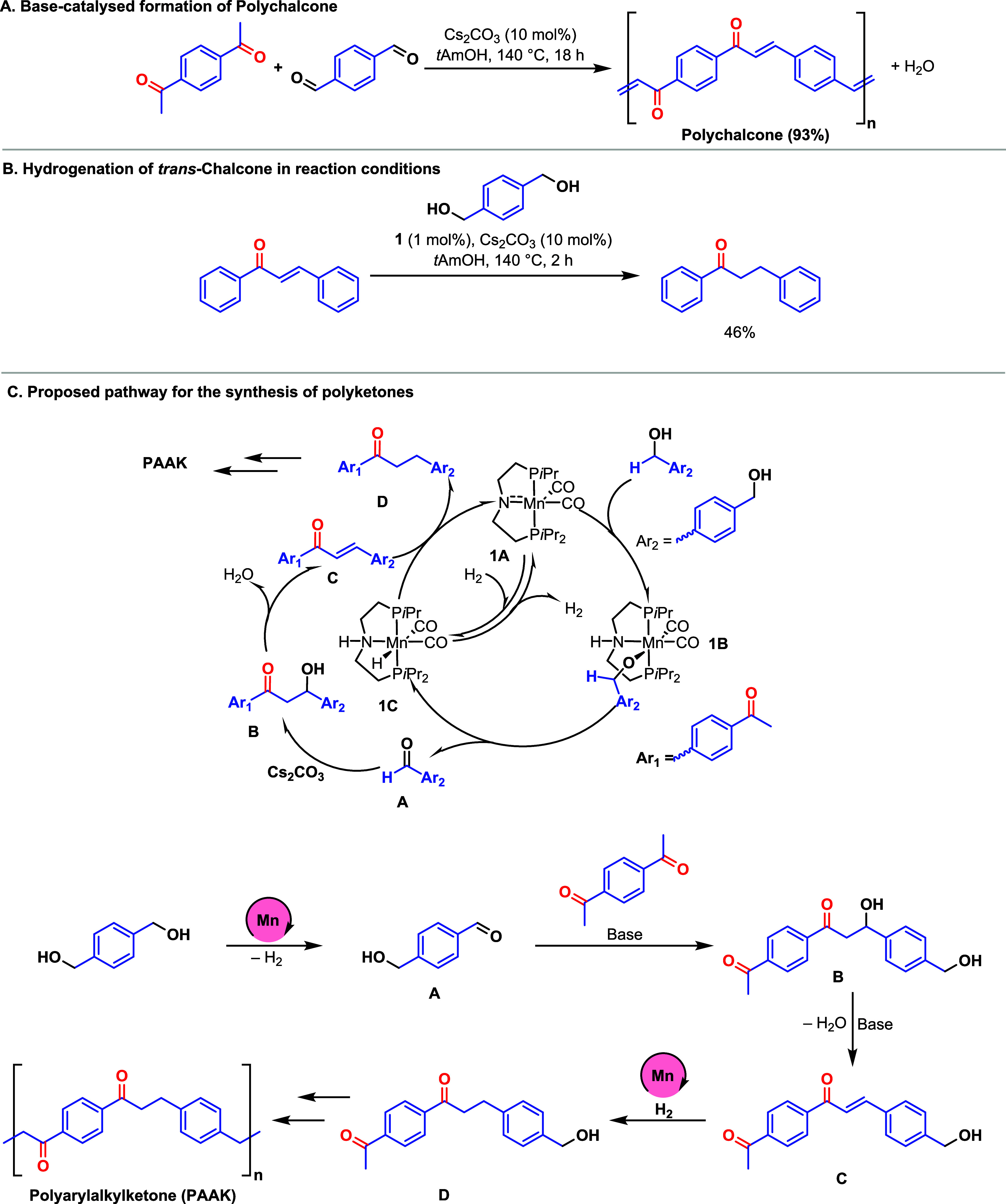
Control Experiments (A, B) and Proposed Pathway for the Formation of Polyketones (C)

Mechanisms for the coupling of ketone and alcohols to form alkylated ketones using analogous pincer complexes have been studied using both experiments and DFT computation.^61,62^ Based on the previous studies,^48^ we hypothesize that the reaction proceeds via a “hydrogen-borrowing” mechanism involving (i) metal-catalyzed dehydrogenation of the alcohol to aldehyde, (ii) base-catalyzed aldol condensation of the aldehyde with the ketone to form a chalcone-type derivative, and (iii) metal-catalyzed hydrogenation of the C=C bond to form an alkylated ketone (Scheme 3C). We conducted a few experiments to verify this proposal. First, performing a reaction of terephthaldehyde with 1,4-diacetylbenzene in the presence of 10 mol % Cs_2_CO_3_ led to the formation of polychalcone in 93% yield (Scheme 3A). This suggests our proposal that Cs_2_CO_3_ is sufficient to catalyze the aldol condensation steps, whereas manganese is needed for the catalytic (de)hydrogenation steps. As described in the mechanism (Scheme 3C), a stoichiometric evolution of hydrogen gas is not observed, as it gets consumed in the subsequent hydrogenation step. In most cases, we observe less than 5 mL of gas. Analysis of this gas by the GC (thermal conductivity detector) confirmed it to be H_2_ supporting our mechanistic proposal (see Section 1.10 in the SI). Furthermore, we also demonstrated that precatalyst **1** is capable of the hydrogenation of C=C in chalcone by transfer hydrogenation from diol under the optimized reaction conditions making dihydrochalcone in 46% yield (Scheme 3B, Section 1.12 in the SI).

We then hypothesized that conducting the catalytic reactions in the presence of a hydrogen atmosphere (1 bar) might ensure the hydrogenation of any remaining C=C bond in the polyketone chain and improve the yield and thermal properties of the polymers. Indeed, performing the synthesis of PAAK-1 in the presence of a hydrogen atmosphere (1 bar) resulted in a higher yield (95 vs 89%) and higher thermal stability (*T*_d_ = 397 vs 363 °C) as described in Table 2, entry 1 (Section 1.8, see the SI). A similar trend was obtained for PAAK-7 (Table 2, entry 7, and Section 1.8 in the SI).

Based on the control experiments described above and mechanistic studies reported in the literature,^48^ we have outlined a mechanism for the formation of polyketone (PAAK, Scheme 3C). The reaction starts with the dehydrogenation of 1,4-benzenedimethanol to a hydroxyaldehyde by the activated manganese complex 1A that converts to the manganese hydride complex 1C via an alkoxy complex 1B. Based on previous studies,^48^ it is likely that the dehydrogenation occurs through an “outer-sphere” mechanism. The formed hydroxyaldehyde can perform aldol condensation with 1,4-diacetylbenzene in the presence of base to form an alkene **C** via intermediate **B**. Alkene **C** can be hydrogenated by manganese hydride complex **1C** to form alkylated ketone. The continuation of this process would lead to the formation of polyketone (PAAK).

## Conclusions

In conclusion, we have demonstrated the synthesis of a new class of polyketones called polyarylalkylketones (PAAK) using a new methodology based on the hydrogen-borrowing concept that has not been used for the synthesis of polyketones before. Among the studied catalysts, the manganese pincer complex **1** was found to be the best catalyst for this process, affording high yields using 0.5–1.0 mol % catalytic loading and in the reaction time as low as 2 h. Using this methodology, 12 new polyketones were synthesized using various diketones and diols including a renewable diol and a diol obtained from the depolymerization of waste plastic bottles. The isolated polymers were characterized by IR and solid-state NMR spectroscopy, GPC, powder XRD, SEM, and TGA/DSC studies. The elasticity modulus and Vickers hardness of PAAK-1 (a polyketone reported herein) estimated using nanoindentation were found to be comparable with a commercial sample of polyketone. Based on previous studies and conducted experiments herein, we suggest that the polymerization occurs via the hydrogen-borrowing mechanism, as outlined in Scheme 3C.

## Data Availability

The raw research data supporting this publication can be accessed at https://doi.org/10.17630/3030ef3c-569c-45d9-ae6c-113de22d1db2.

## Supporting Information Available

Experimental details; characterization data; TGA, DSC curves; PXRD; and NMR spectra (PDF)
