# Supplementary material for: Manganese-Catalyzed Synthesis of Polyketones Using Hydrogen-Borrowing Approach
Source: ACS Catal. 2024 Jun 28;14(14):10624–34. doi: 10.1021/acscatal.4c03019 (PMC11264210; doi:10.1021/acscatal.4c03019)
Supplement: Supplementary file 1 — cs4c03019_si_001.pdf [file cs4c03019_si_001.pdf]

Manganese Catalysed Synthesis of Polyketones using Hydrogen Borrowing Approach

Pavel Kulyabin,[†] Oxana V. Magdysyuk,[†] Aaron B Naden,[†] Daniel M Dawson,[†] Ketan Pancholi,[δ] Matthew Walker,[γ] Massimo Vassalli,[χ] and Amit Kumar [†]*

[†]EaStCHEM, School of Chemistry, University of St Andrews, North Haugh, St Andrews, KY16 9ST, UK.
[δ] The Sir Ian Wood Building, Robert Gordon University, Garthdee Rd, Garthdee, Aberdeen, AB10 7GE, UK.
[γ]Centre for the Cellular Microenvironment, Advanced Research Centre, University of Glasgow, Glasgow, G116EW, UK.
[χ] James Watt School of Engineering, University of Glasgow, Glasgow G12 8QQ, UK.
E-mail: ak336@st-andrews.ac.uk

Contents

1. Experimental Details 2
- 1.1 General Considerations 2
- 1.2 Synthesis of 3,3'-(1,4-phenylene)bis(1-phenylpropan-1-one) as a model substrate. 4
- 1.3 Coupling of 1,4-diacetylbenzene and 1,4-benzenedimethanol. 6
- 1.4 NMR spectra of mother liquor from coupling reaction of 1,4-diacetylbenzene and 1,4-benzenedimethanol as described in Table S1. 29
- 1.5 Synthesis of polyketones from diketones and diols. 30
- 1.6 Syntheses of polychalcones from diketones and dialdehydes 69
- 1.7 Reaction 4-acetylphenyl ether and potassium *tert*-butoxide. 73
- 1.8 Syntheses of polyketones in the presence of hydrogen atmosphere. 75
- 1.9 Catalyst reuse experiments. 79
- 1.10 Headspace gas analysis from the synthesis of polyketone PAAK-7. 80
- 1.11 Hydrogenative depolymerization of polyethyleneterephtalate (PET). 81
- 1.12 Hydrogenation of trans-Chalcone by transfer hydrogenation. 82
- 1.13 Mass spectroscopy investigation of mother liquor from reaction mixtures. 83
- 1.14 TGA and DSC analysis combined with mass-spectrometry. 85
- 1.15 Infrared spectra of starting materials and commercially available compounds. 86
- 1.16 Thermal properties and GPC analysis of commercial polyketones. 90
- 1.17 Powder XRD patterns of starting materials. 94
- 1.18 Scanning Electron Microscopy 96
- 1.19 Powder XRD and Scanning Electron Microscopy analysis 101

2. Mechanical properties 102

3. References 105

1. Experimental Details

1.1 General Considerations

All manipulations, unless otherwise stated, were performed under an argon atmosphere using standard Schlenk line and glove-box techniques. Glassware was oven-dried and flamed under vacuum prior to use. THF and toluene were dried using a Grubbs-type solvent purification system (Innovative Technologies SPS) equipped with a degasser. Pre-catalyst **1**[1] was prepared in accordance with the literature procedure. Cs_2CO_3 (anhydrous) were stored at 80 °C and dried before use. Precatalysts **2–4**, $Mn(CO)_5Br$, triphenylphosphine, NMR solvents and iPr-PN^{H}P (10 wt% solution in THF) were purchased from Strem Chemicals and used as received. Polyolefinketone (POK), polyetherketoneketone (PEKK) and polyetheretherketone (PEEK) samples were purchased from Goodfellow Cambridge Limited and used as received.

Solution state NMR spectra were recorded on a Bruker AVIII-HD 400 MHz NMR spectrometer at 298 K unless otherwise specified. Residual protons of solvent were used as a reference for ^{1}H NMR spectra in deuterated solvent samples. All chemical shifts (δ) are quoted in ppm and coupling constants (*J*) in Hz.

Solid-state ^{13}C NMR spectra were recorded using a Bruker Avance III spectrometer, equipped with a 9.4 T wide-bore superconducting magnet (^{1}H and ^{13}C Larmor frequencies of 400.1 and 100.6 MHz, respectively). Some samples were submerged in liquid nitrogen and then ground to a powder. The remaining nitrogen and any condensed water were allowed to evaporate. Samples were packed into 4 mm zirconia magic angle spinning (MAS) rotors and rotated at a MAS rate of 12.5 kHz. ^{13}C NMR spectra were recorded with cross polarisation (CP) from ^{1}H using a contact pulse (ramped for ^{1}H) of between 0.5 and 2 ms. Signal averaging was carried out for 2048 transients (NM118(II)) with a recycle interval of 2 s. High power ($\nu 1 \approx 100$ kHz) TPPM-15 decoupling of ^{1}H was applied during acquisition. Chemical shift is reported in ppm relative to $(CH_3)_4Si$ using the CH_3 signal of L-alanine (δ = 20.5 ppm) as a secondary solid reference.

Solid state ^{1}H MAS NMR spectra were recorded at 600.26 MHz using a Bruker Avance III spectrometer equipped with a 14.1 T wide-bore superconducting magnet. Samples were packed into a 1.9 mm zirconia rotors and rotated at the magic angle at a rate of 40 kHz. A rotor-synchronised spin echo pulse sequence (τ = 25 μs) was used to remove background signal. Signal averaging was carried out for 48 transients with a recycle interval of 5 s. Chemical shifts are reported in ppm relative to $(CH_3)_4Si$ using the NH_3 signal of L-alanine (δ = 8.5 ppm) as a secondary solid reference.

Infrared spectra (ATR-FTIR) were collected using a Shimadzu IRAffinity-1.

Thermogravimetric Analysis (TGA) was performed using Stanton Redcroft STA-780 Series Thermal Analyser between 30–900 °C at a heating rate of 10 °C/min under a flow of nitrogen gas (25 mL/min). Decomposition temperature (T_d, °C) was estimated as the temperature of 10% weight loss.

Differential Scanning Calorimetry (DSC) analyses were performed using a Netzsch DSC204 or Queens STA449 DSC217C between –80 or –10 to 250, 300 or 600 °C at a heating rate of 10 °C/min under a flow of nitrogen gas (20 mL/min) after an initial heat/cool cycle (25–120 °C at 10 °C/min with a 20-minute isothermal at 120 °C) to remove the thermal history of the sample.

Differential Scanning Calorimetry with Mass Spectroscopy (DSC-MS) analyses were performed using a Netzsch STA 449F5 30–600 °C at a heating rate of 10 °C/min under a flow of argon gas (20 mL/min).

Gel permeation chromatography (GPC) was performed on an Agilent 1260 InfinityLab II GPC fitted with a refractive index (RI) detector (35 °C). Two plus guard column Agilent PLgel-M 10 μm MIXED-B columns setup was contained within an oven (35 °C). Mixture of chloroform (80%) and dichloroacetic acid (20%) was used as the eluent at a flow rate of 1.0 mL min^{-1}. The samples (30 mg) were heated in 4 mL of dichloroacetic acid at 120 °C overnight. Next, the samples were diluted to the required concentration of 0.2% (w/v) with chloroform and injected into a system running at temperature after filtration to remove

undissolved material. The calibration was conducted using a series of polystyrene (M_n = 1,000 – 243,000 g mol^{-1}) standards obtained from Agilent Technologies.

Scanning electron microscopy (SEM) was performed on an FEI Scios dualbeam instrument operated at 3 kV, samples were prepared by dispersion onto adhesive carbon tape (Labtech International Ltd.) and sputter coated with a thin layer of gold to dissipate the charge.

Powder X-ray diffraction data was collecteds using Bruker AXS D8 Advance diffractometer with a Vantec detector, using Cu Kα radiation. The data from powder samples were collected in the range of $2° < 2\theta < 70$ or 90°. Some samples were measured on a PTFE pad which led to appearance of additional peaks (originated from PTFE) at high 2θ. Broad peak at $2\theta = 26°$ in several samples originates from the polyester film that was used to cover the powder.

GC-MS data were collected as solutions in HPLC grade DCM using an Agilent 8860 GC system coupled to an Agilent 5977B EI instrument. EI spectra were collected as solutions in acetonitrile using a Micromass LCT spectrometer.

Headspace analysis was performed using an Agilent GC8860 with TCD. Gas separation is performed using dual columns (Agilent porous Polymer and Agilent Mol sieve). Gas sample (2.5 mL) is introduced to the columns using a gas tight syringe through a sample loop (0.25 mL).

1.2 Synthesis of 3,3'-(1,4-phenylene)bis(1-phenylpropan-1-one) as a model substrate.

A 15 mL pressure vessel was charged with pre-catalyst **1** (10 mg, 0.02 mmol, 2 mol%), 1,4-benzenedimethanol (138 mg, 1.0 mmol), acetophenone (240 mg, 2.0 mmol) and Cs_2CO_3 (33 mg, 0.10 mmol, 10 mol%). *tert*-Amyl alcohol (5 mL) was added, and the flask was sealed under a nitrogen atmosphere in a glove box before heating to 140 °C for 18 hours with stirring. After this period, the reaction vessel was allowed to cool to room temperature. Next, the crude was directly purified by flash chromatography on silica gel to afford, after concentration and high-vacuum drying, 284 mg (83%) of the product as a white solid. ^{1}H NMR (400 MHz, $CDCl_3$): δ 7.96 (m, 4H), 7.56 (m, 2H), 7.47 (m, 4H), 7.20 (s, 4H), 3.30 (m, 4H), 3.05 (m, 4H). ^{13}C NMR (100 MHz, $CDCl_3$): δ 199.2, 139.0, 136.8, 133.0, 128.6, 128.0, 40.4, 29.9. IR (ATR-FTIR, cm^{-1}): ν 2920w (C-H), 1680s (C=O), 1593w, 1516w, 1447m, 1204m, 972m, 745s, 691s, 548m. $[M+Na]^+$ Calcd for $C_{24}H_{22}NaO_2^+$, 365.1512; found: 365.1510.

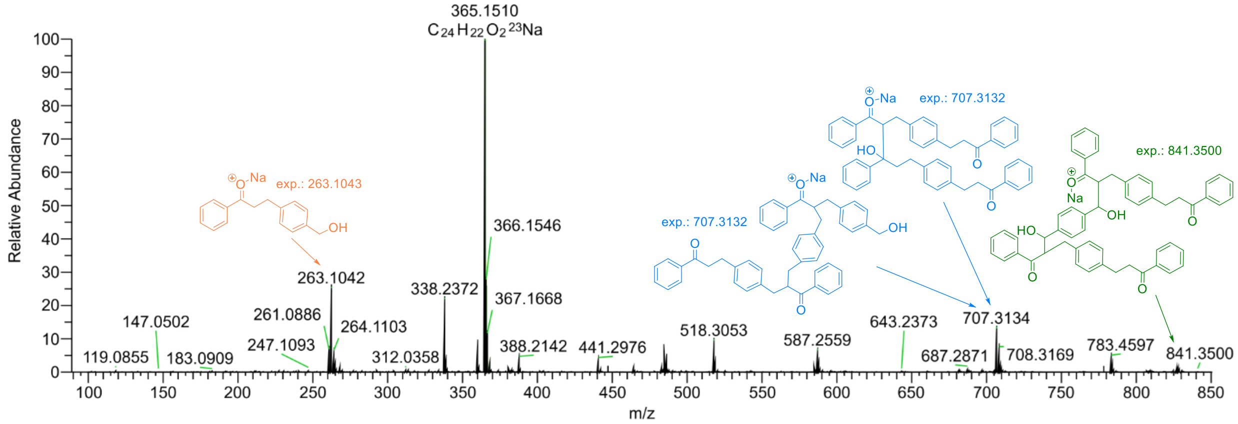

Figure S1. High Resolution Mass Spectrum (HRMS, ESI, MeCN) of crude reaction mixture after synthesis of 3,3'-(1,4-phenylene)bis(1-phenylpropan-1-one).

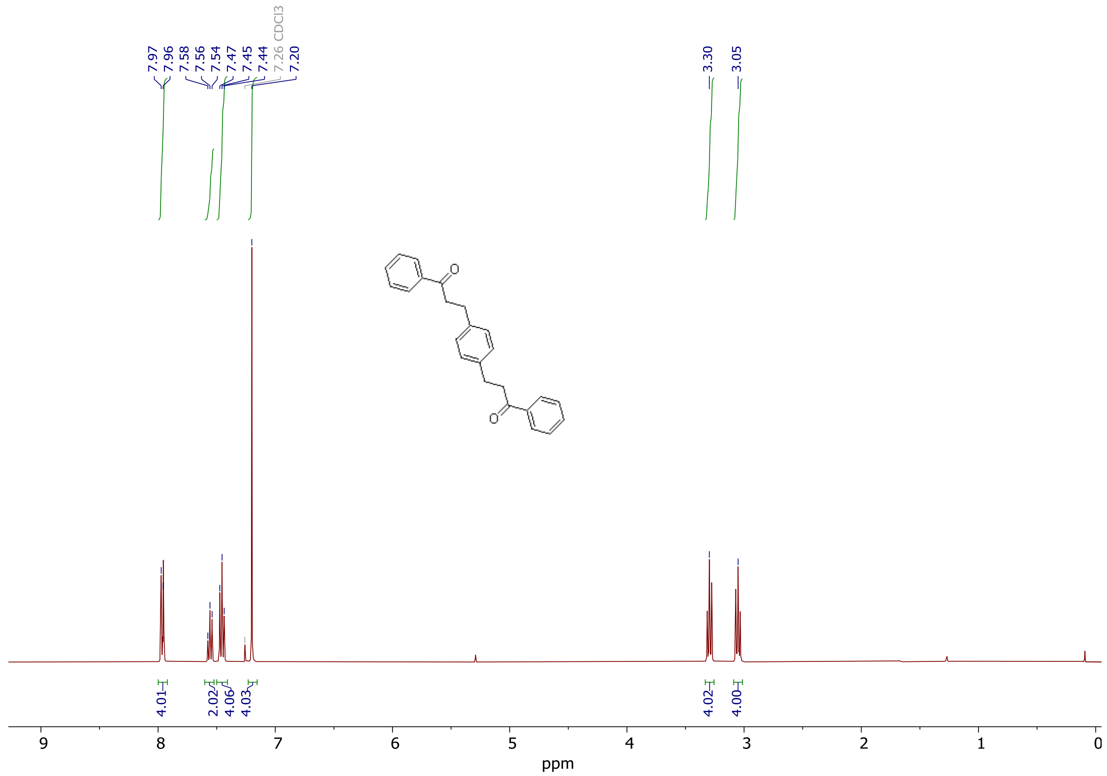

Figure S2. ^{1}H NMR ($CDCl_3$, 298 K) spectrum of 3,3'-(1,4-phenylene)bis(1-phenylpropan-1-one).

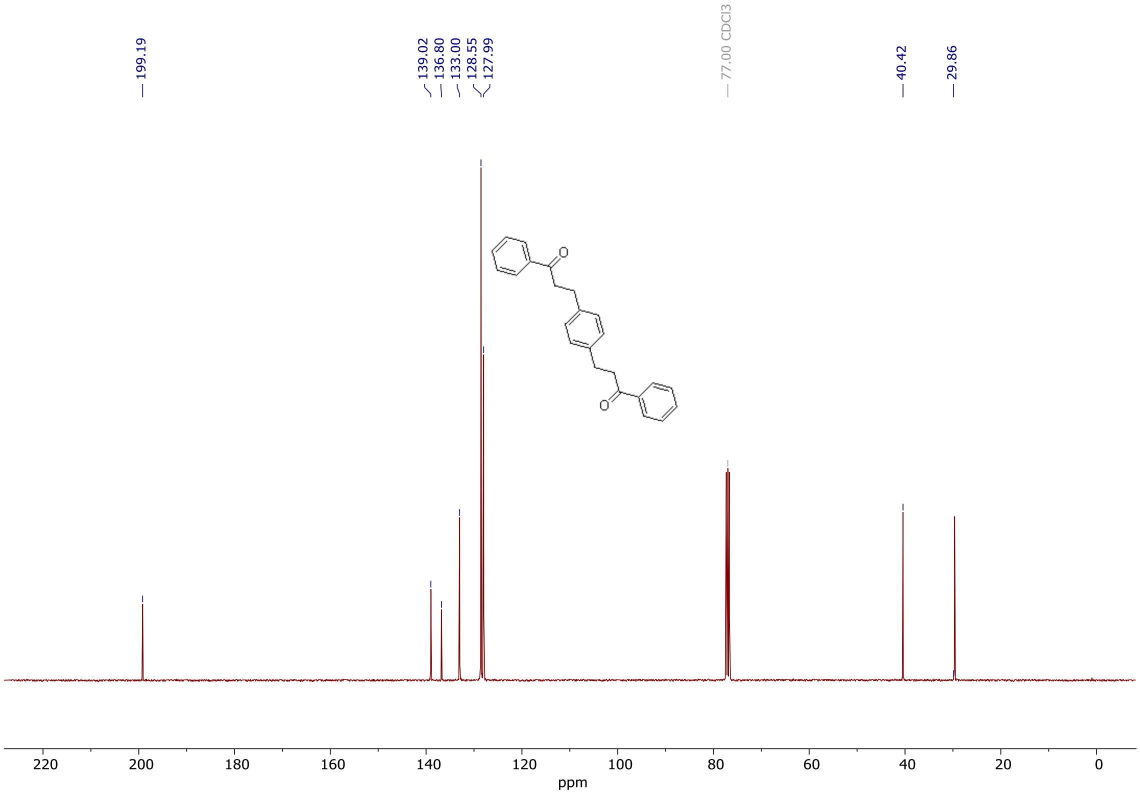

Figure S3. $^{13}C\{^1H\}$ NMR ($CDCl_3$, 298 K) spectrum of 3,3'-(1,4-phenylene)bis(1-phenylpropan-1-one).

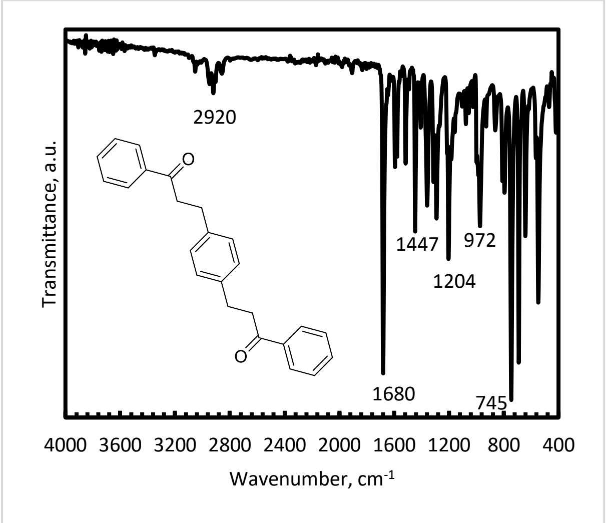

Figure S4. Infrared spectrum (ATR-FTIR) of 3,3'-(1,4-phenylene)bis(1-phenylpropan-1-one).

1.3 Coupling of 1,4-diacetylbenzene and 1,4-benzenedimethanol.

A 100 mL ampoule equipped with a J-Young's valve or a 15 mL pressure vessel was charged with pre-catalyst (*e.g.* **1**; 10 mg, 0.02 mmol, 2 mol%), 1,4-benzenedimethanol (138 mg, 1.0 mmol), 1,4-diacetylketone (162 mg, 1.0 mmol) and Cs_2CO_3 (*e.g.* 33 mg, 0.10 mmol, 10 mol%). *Tert*-amyl alcohol or toluene (*e.g.* 5 mL) was added and the flask was sealed under an argon atmosphere before heating to the desired temperature (*e.g.* 140 °C) for the desired length of time (*e.g.* 18 hours) with stirring. After this period, the reaction vessel was allowed to cool to room temperature and any gas evolved (presumably H_2) during the reaction measured using inverted cylinder in water method. To the resulting mixture, 5 mL of 1 M HCl was added and the flask had been heated at 90 °C for 1 h. The yellow precipitate was filtered and dried under reduced pressure at 120 °C.

Table S1. Polyketone synthesis from 1.4-diacetylbenzene and 1,4-benzenedimethanol.[a]

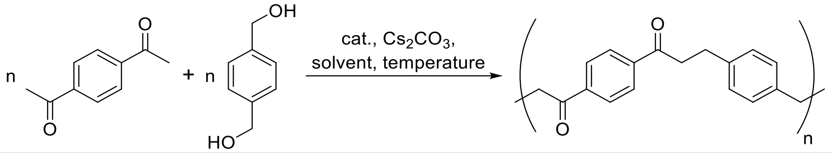

Entry	Complex	mol%	Conc.	Base (mol%)	Time (h)	Isolated Yield (%)	T_d[j] (°C)
1[b,c]	**1**	2	0.2 M	10%	18	58[d]	325/266
2[b]	**1**	2	0.2 M	10%	18	89	338
3[b]	**2**	2	0.2 M	10%	18	< 5	n.d.
4[b]	**3**	2	0.2 M	10%	18	51	353
5[b]	**4**	2	0.2 M	10%	18	17	319
6[b]	**1**	1	0.2 M	10%	18	87	356
7[b]	**1**	0.5	0.2 M	10%	18	70	348
8	**1**	1	0.1 M	10%	18	85	373
9[e]	**1**	1	0.1 M	10%	18	73	342
10	**1**	1	0.1 M	20%	18	90	369
11	**1**	1	0.1 M	3%	18	80	335
12[f]	**1**	1	0.1 M	10%	18	67	328
13[g]	**1**	1	0.25 M	10%	18	94	352
14[h]	**1**	1	0.05 M	10%	18	86	342
15	**1**	1	0.1 M	10%	2	89	363
16	**1**	1	0.1 M	10%	1	81	343
17	**1**	1	0.1 M	3%	2	81	344
18	**none**	none	0.1 M	10%	18	8	396
19	**1**	1	0.1 M	none	18	none	—
20[i]	**1**	1	0.1 M	1%[i]	18	none	—
21[i]	**1**	1	0.1 M	2%[i]	18	< 5	—
22	**5**	1	0.1 M	10%	18	< 5	n.d.
23	**6**	1	0.1 M	10%	18	< 5	n.d.

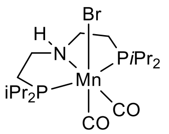

1

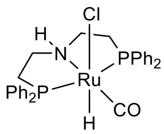

2

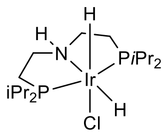

3

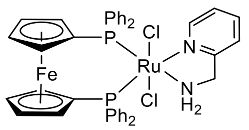

4

Mn(CO)5Br
5

Mn(CO)5Br
+
3 eq. PPh3
6

[a]General reaction conditions: 1,4-diacetylbenzene (0.5 mmol), 1,4-benzenedimethanol (0.5 mmol), Cs_2CO_3, metal-complex, and 5 mL of *t*AmOH were placed into 100 mL ampule with J-Young's valve under an argon atmosphere and heated at 140 °C for 18 h. [b]1 mmol of diol and diketone were used. [c]Toluene was used instead of *t*AmOH. [d]The reaction mixture consisted of two kinds of solid material: red chunk in the bottom (38%) and yellow brittle film on the edge of solution (20%) which were separated mechanically. [e]Reaction was carried out in 15 mL pressure vessel. [f]Reaction at 110°C. [g]2 ml of *t*AmOH were used. [h]10 mL of *t*AmOH were used. [i]KO*t*Bu was used for catalyst activation. [j]Temperature of degradation. Calculated from TGA as a temperature of 10% weight loss.

Table S1, Entry 1
Yellow solid (21%):
IR (ATR-FTIR, cm^{-1}): ν 3470w (O-H), 3049w (C-H), 2922w (C-H), 1674s (C=O), 1601m (C=C), 1506w, 1406m, 1265s, 1213s, 982s, 818s, 731s, 544m.
TGA: T_d = 325 °C.
Red solid (38%):
3348m (O-H), 2924w (C-H), 1674s (C=O), 1605m (C-H), 1510w, 1373m, 1217m, 1213m, 1011m, 816s, 517s.
TGA: T_d = 266 °C.

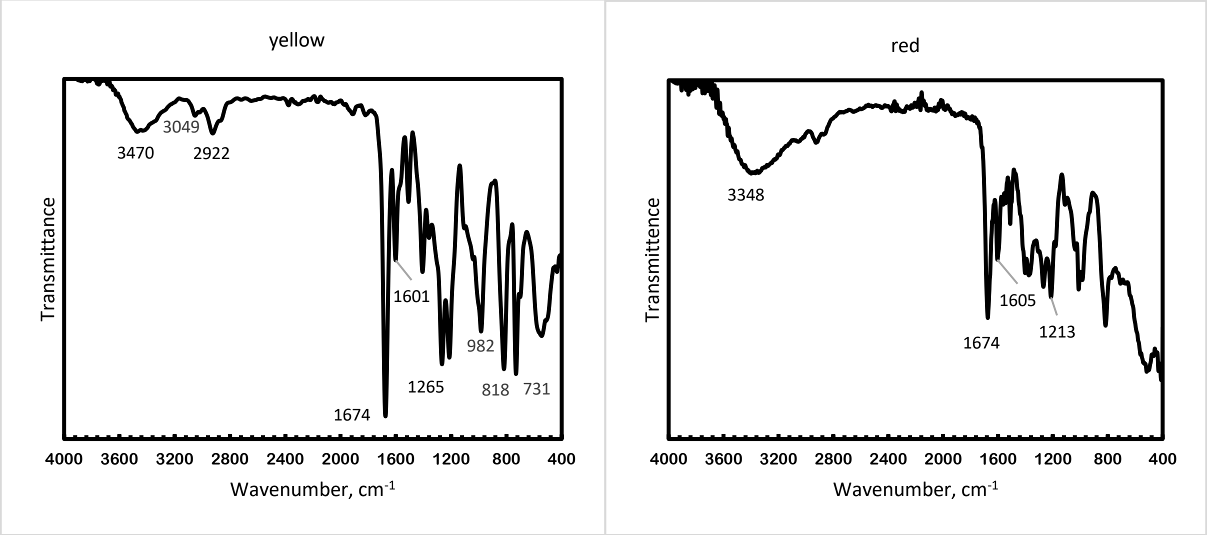

Figure S5. Infrared spectrum (ATR-FTIR) of the samples corresponding to Entry 1 in Table S1.

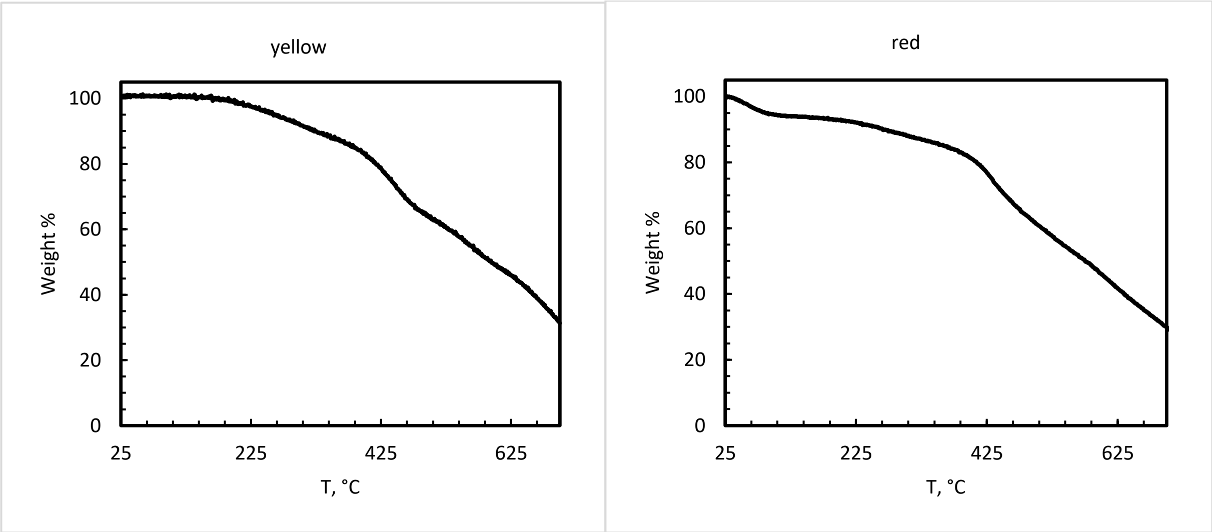

Figure S6. Mass loss as a function of temperature for samples corresponding to Entry 1 in Table S1.

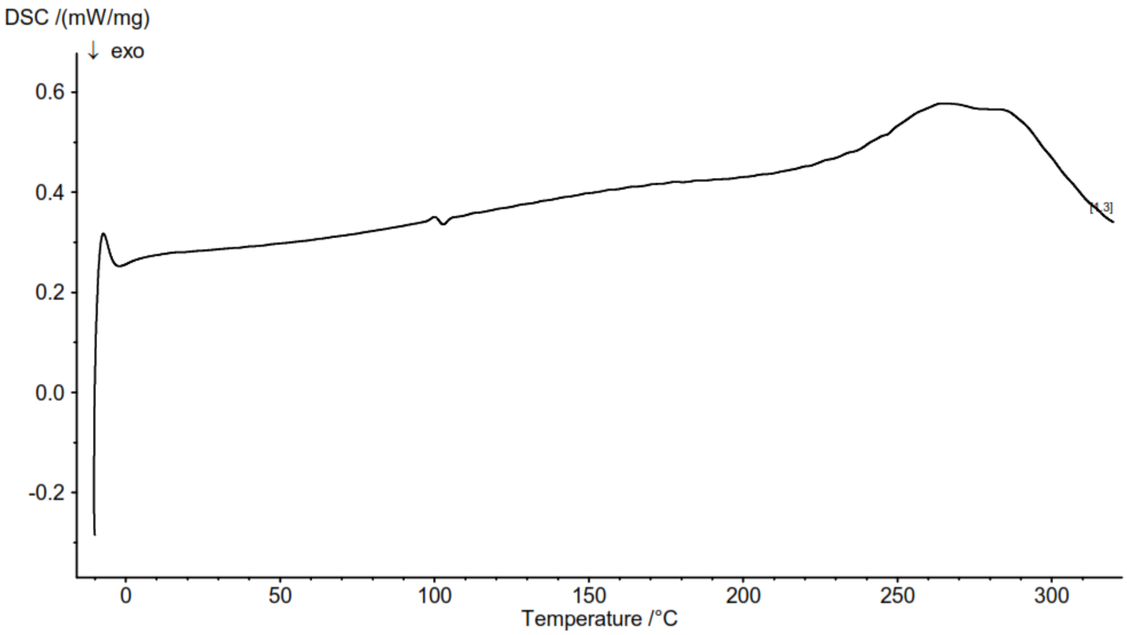

Figure S7. DSC trace corresponding to Entry 1 in Table S1, yellow solid.

Table S1, Entry 2
^{13}C CP MAS NMR (100.6 MHz): δ 199.5, 151.6, 139.9, 128.4, 75.7, 70.2, 64.4, 42.2, 29.3.
IR (ATR-FTIR, cm^{-1}): ν 3460w (O-H), 2926w (C-H), 1674s (C=O), 1603m (C=C), 1508w, 1406m, 1225s, 1211s, 982s, 818s, 546m.
TGA: T_d = 338 °C

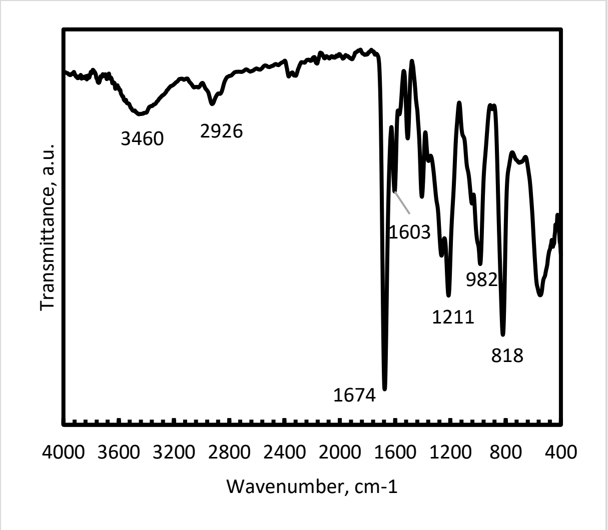

Figure S8. Infrared spectrum (ATR-FTIR) of the sample corresponding to Entry 2 in Table S1.

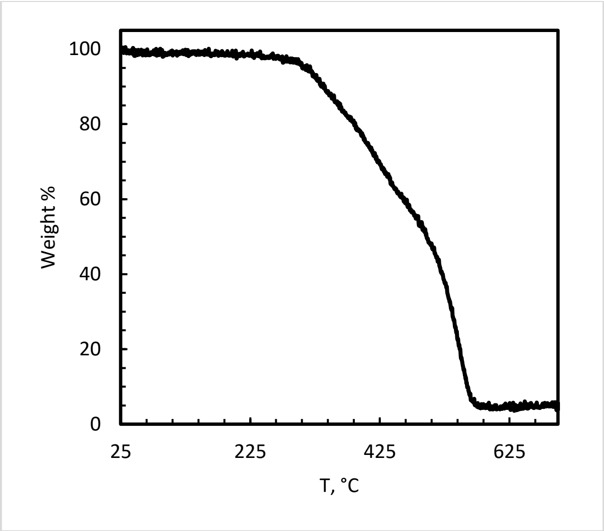

Figure S9. Mass loss as a function of temperature for sample corresponding to Entry 2 in Table S1.

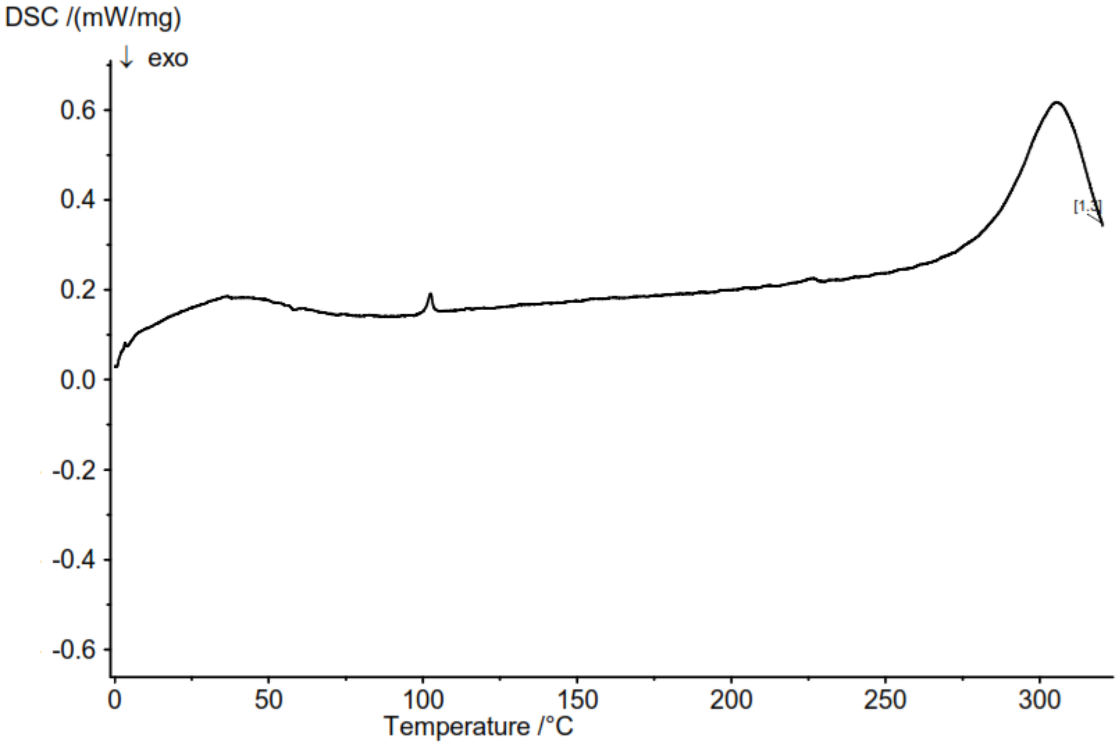

Figure S10. DSC trace corresponding to Entry 2 in Table S1.

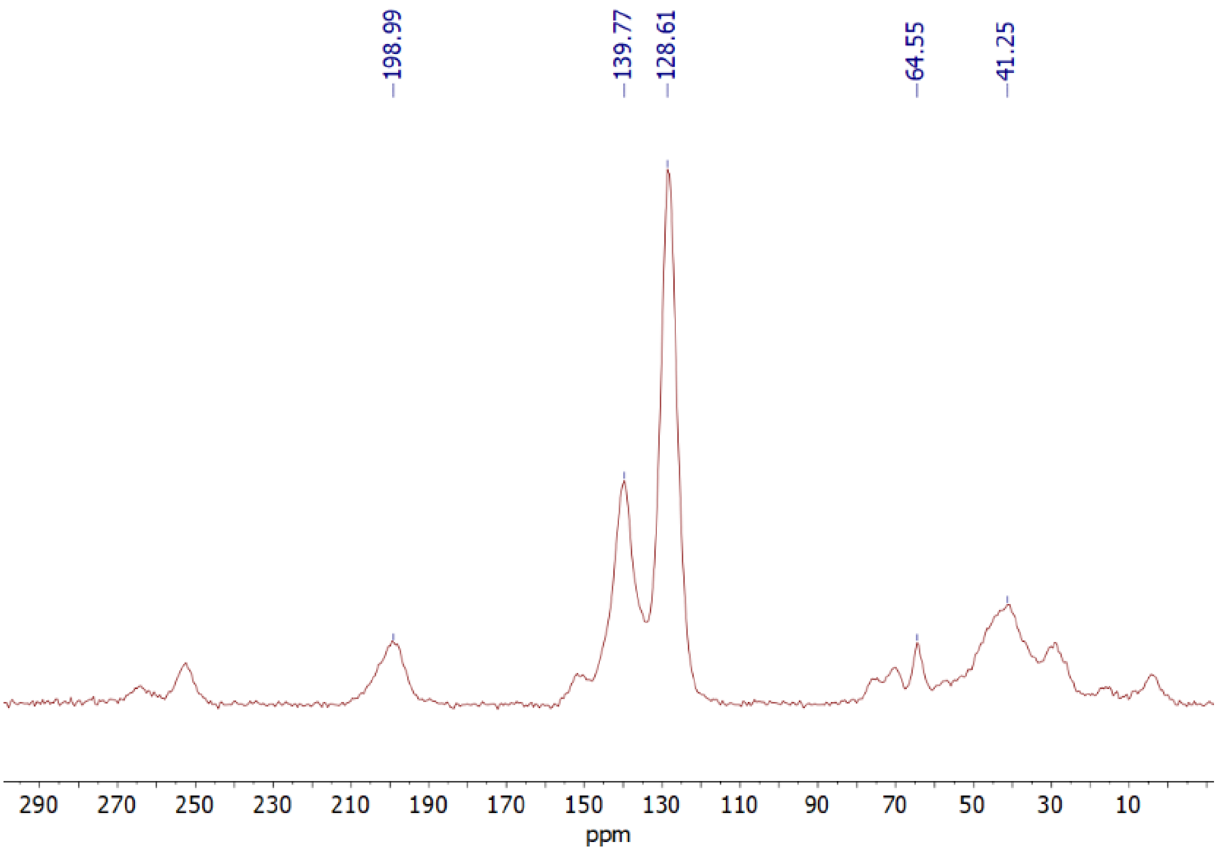

Figure S11. ^{13}C CP MAS NMR spectrum of the polymer corresponding to Entry 2 in Table S1.

<u>Table S1, Entry 3</u>
The isolated amount was not enough to perform analysis.

<u>Table S1, Entry 4</u>
IR (ATR-FTIR, cm^{-1}): ν 3368w (O-H), 2920w (C-H), 1690w, 1672s (C=O), 1607w (C=C), 1510w, 1400m, 1267s, 978m, 814s.
TGA: T_d = 353 °C
DSC: T_m= 215 °C

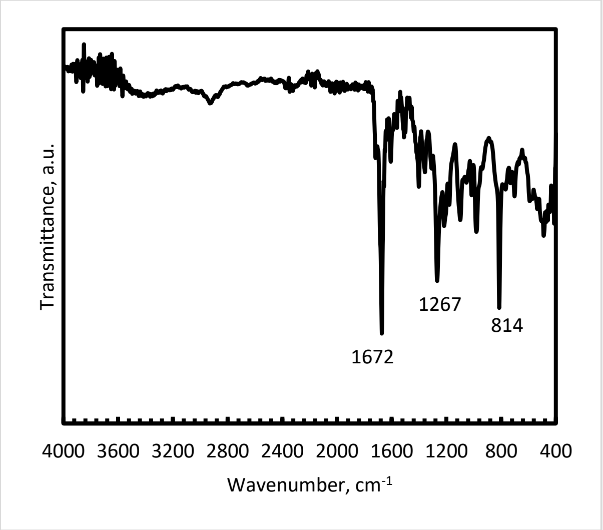

Figure S12. Infrared spectrum (ATR-FTIR) of the sample corresponding to Entry 4 in Table S1.

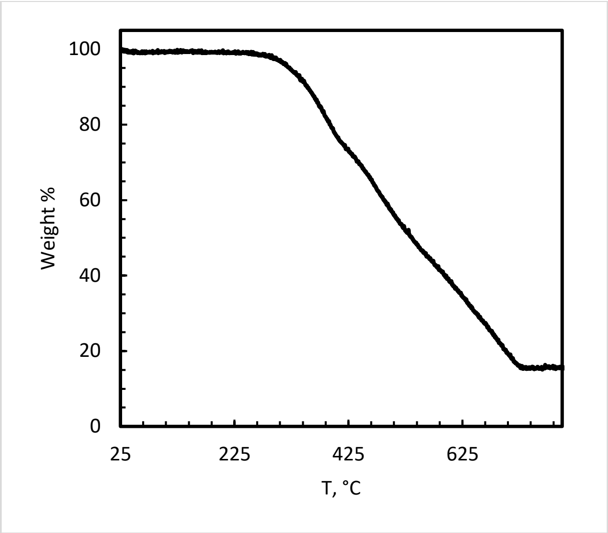

Figure S13. Mass loss as a function of temperature for sample corresponding to Entry 4 in Table S1.

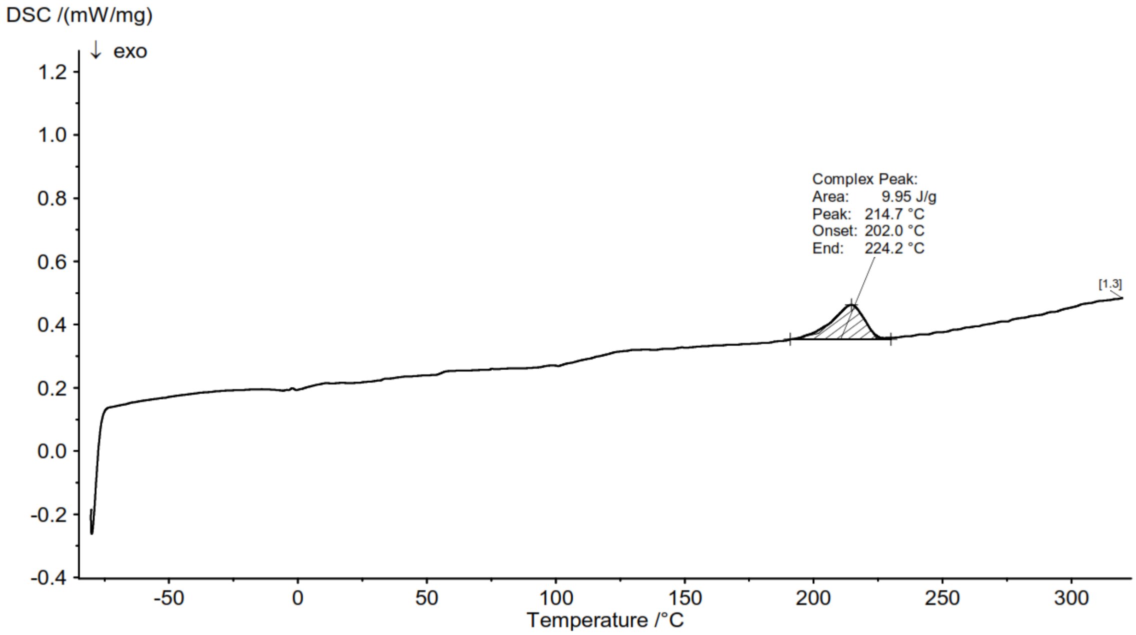

Figure S14. DSC trace corresponding to Entry 4 in Table S1.

Table S1, Entry 5
IR (ATR-FTIR, cm^{-1}): ν 3416w (O-H), 2911w (C-H), 1676s (C=O), 1605m (C=C), 1506s, 1402m, 1265s, 1213s, 988s, 820s.
TGA: T_d = 319 °C

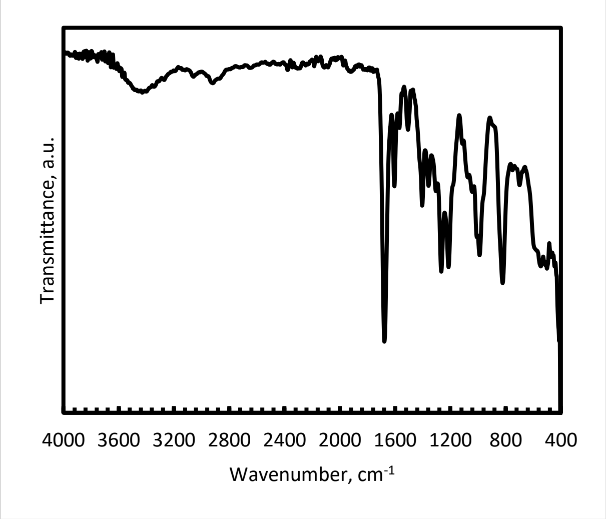

Figure S15. Infrared spectrum (ATR-FTIR) of the sample corresponding to Entry 5 in Table S1.

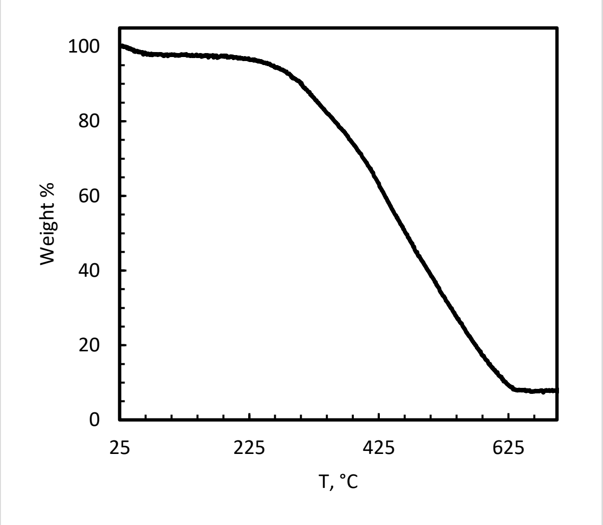

Figure S16. Mass loss as a function of temperature for sample corresponding to Entry 5 in Table S1.

Table S1, Entry 6
IR (ATR-FTIR, cm^{-1}): ν 3424w (O-H), 2924w (C-H), 1676s (C=O), 1605m (C=C), 1510s, 1402m, 1225m, 1213s, 984m, 818s, 542m.
TGA: T_d = 356 °C

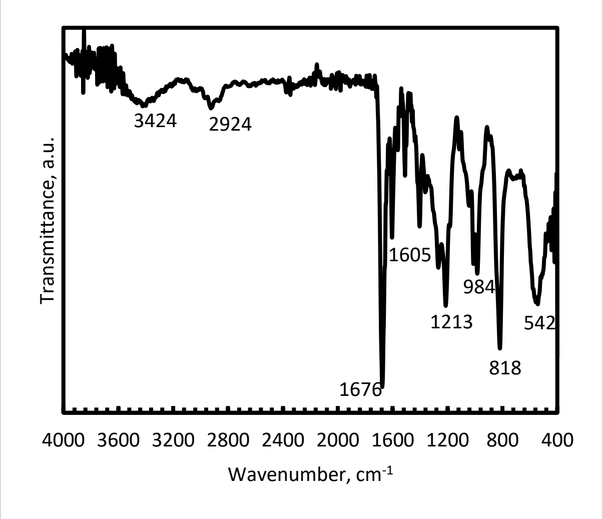

Figure S17. Infrared spectrum (ATR-FTIR) of the sample corresponding to Entry 6 in Table S1.

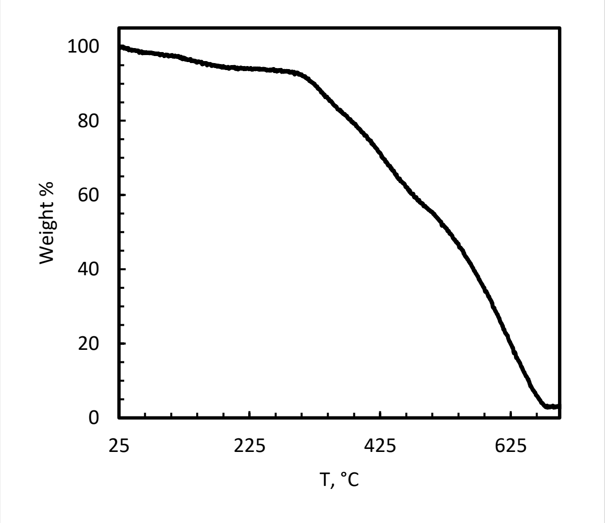

Figure S18. Mass loss as a function of temperature for sample corresponding to Entry 6 in Table S1.

Table S1, Entry 7
IR (ATR-FTIR, cm^{-1}): ν 3420w (O-H), 2918w (C-H), 1674s (C=O), 1603m (C=C), 1422w, 1402m, 1265m, 1211s, 982s, 818s, 550m.
TGA: T_d = 348 °C

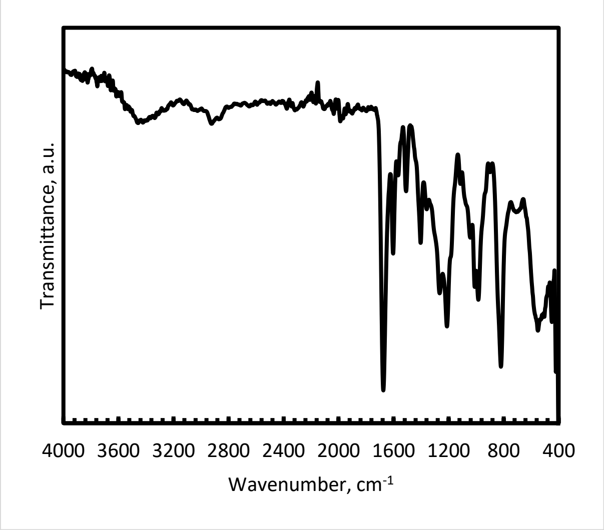

Figure S19. Infrared spectrum (ATR-FTIR) of the sample corresponding to Entry 7 in Table S1.

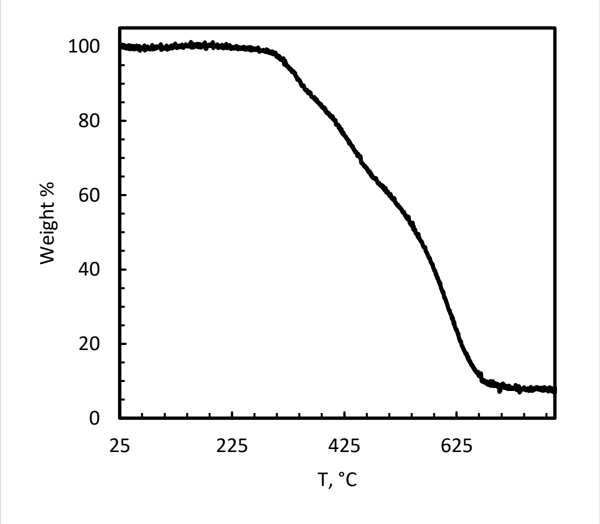

Figure S20. Mass loss as a function of temperature for sample corresponding to Entry 7 in Table S1.

Table S1, Entry 8
IR (ATR-FTIR, cm^{-1}): ν 3402w (O-H), 2914w (C-H), 1674s (C=O), 1603m (C=C), 1510w, 1402m, 1225m, 1211s, 982m, 818s, 550m.
TGA: T_d = 373 °C

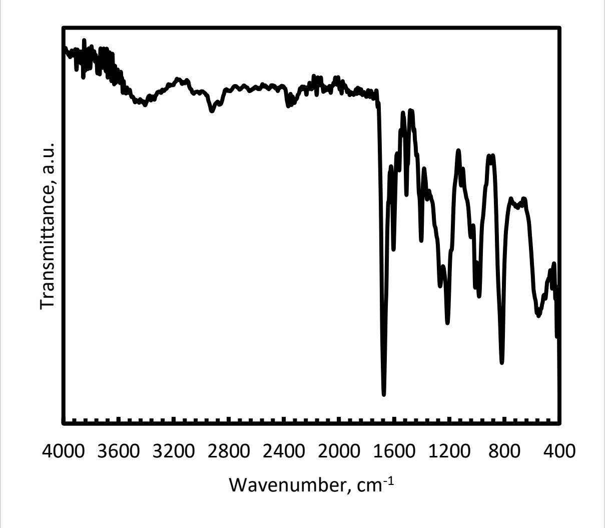

Figure S21. Infrared spectrum (ATR-FTIR) of the sample corresponding to Entry 8 in Table S1.

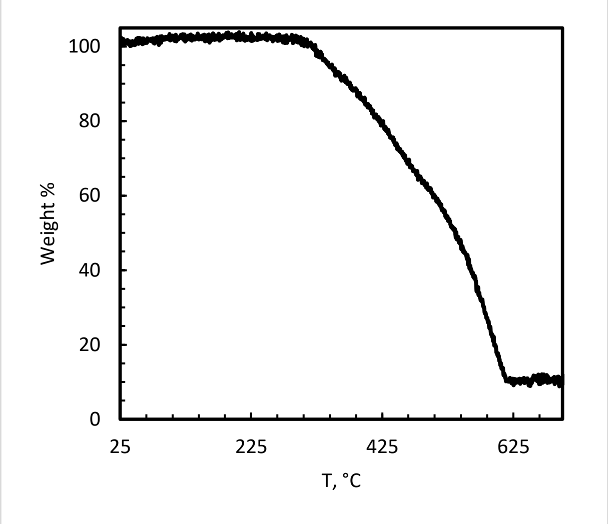

Figure S22. Mass loss as a function of temperature for sample corresponding to Entry 8 in Table S1.

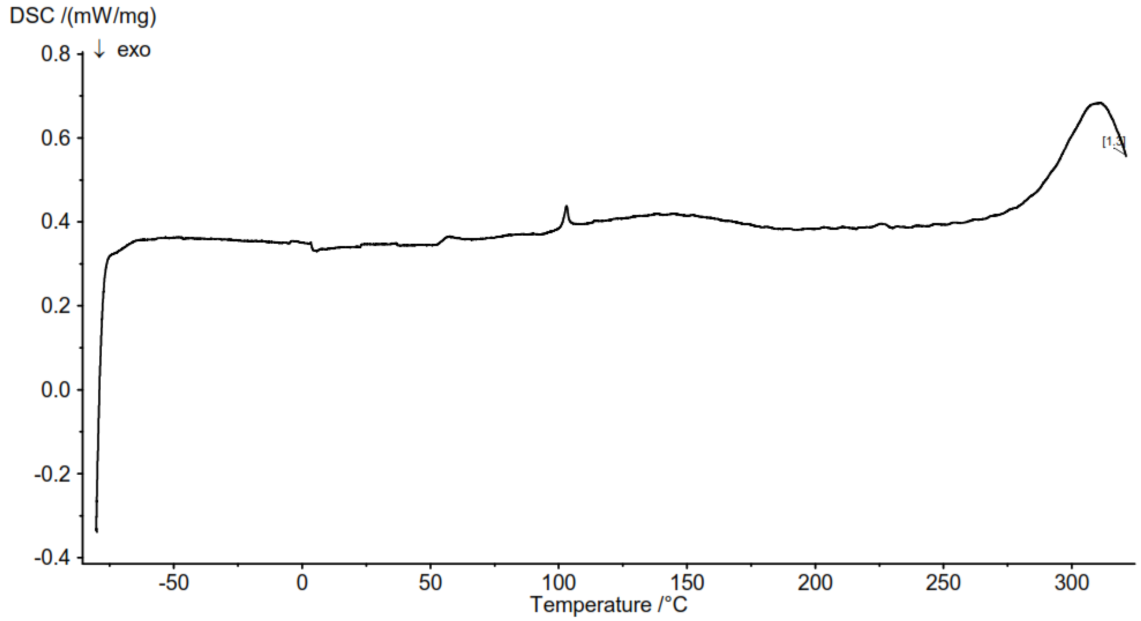

Figure S23. DSC trace corresponding to Entry 8 in Table S1.

<u>Table S1, Entry 9</u>
IR (ATR-FTIR, cm^{-1}): ν 3449w (O-H), 2911w (C-H), 1676s (C=O), 1605m (C=C), 1510w, 1402m, 1221m, 1211s, 982s, 818s.
TGA: T_d = 342 °C
DSC: T_g = 247 °C

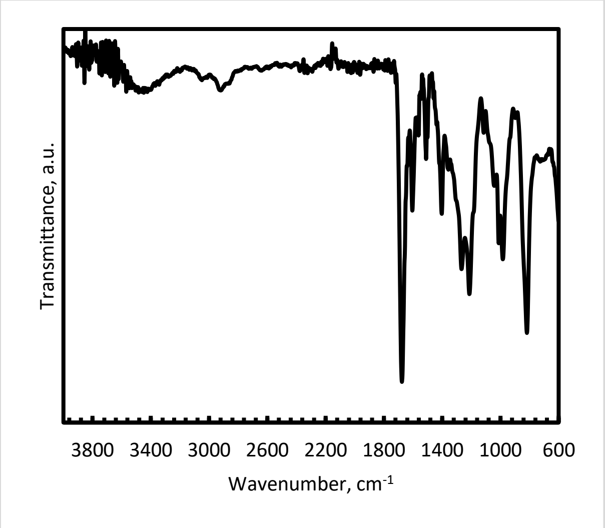

Figure S24. Infrared spectrum (ATR-FTIR) of the sample corresponding to Entry 9 in Table S1.

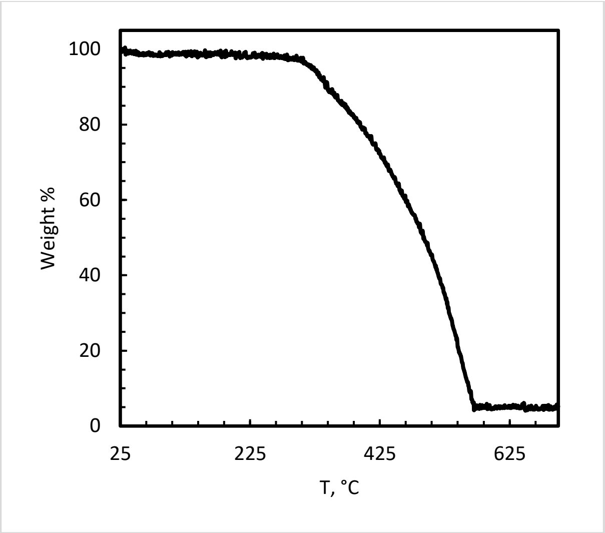

Figure S25. Mass loss as a function of temperature for sample corresponding to Entry 9 in Table S1.

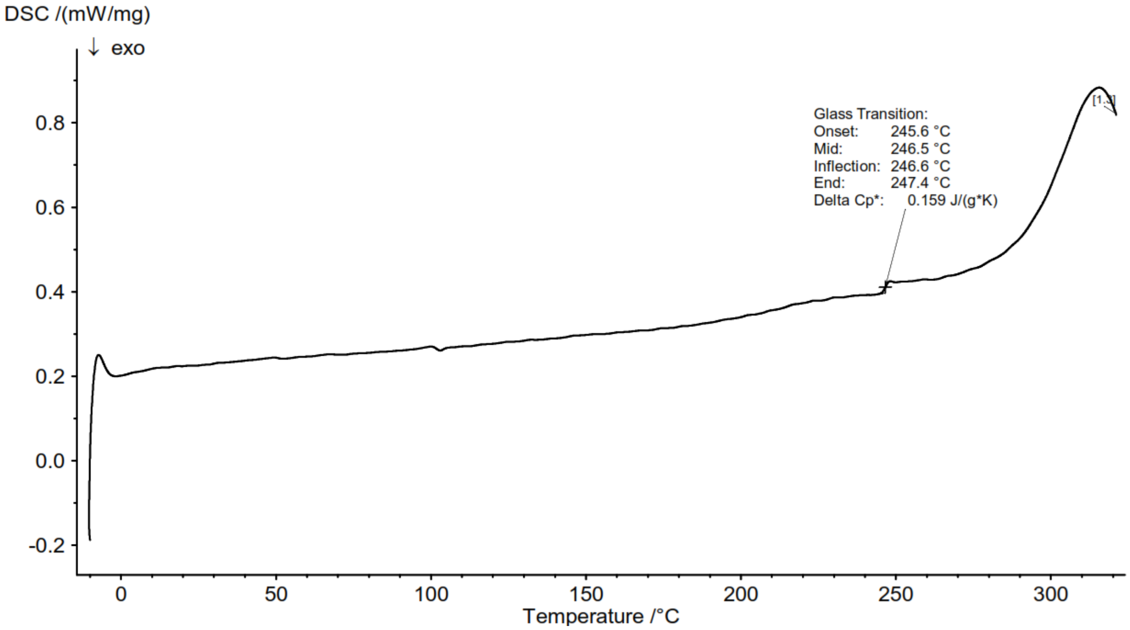

Figure S26. DSC trace corresponding to Table S1; Entry 9.

Table S1, Entry 10
IR (ATR-FTIR, cm^{-1}): ν 3464w (O-H), 2922w (C-H), 1676s (C=O), 1605m (C=C), 1418w, 1404m, 1220m, 1213s, 984m, 818s.
TGA: T_d = 369 °C

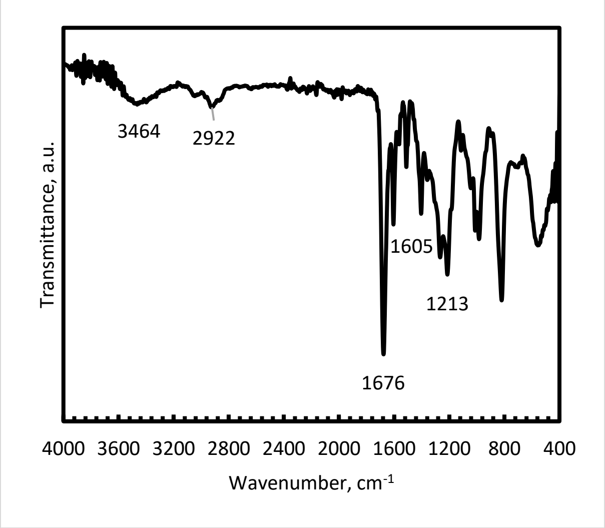

Figure S27. Infrared spectrum (ATR-FTIR) of the sample corresponding to Entry 10 in Table S1.

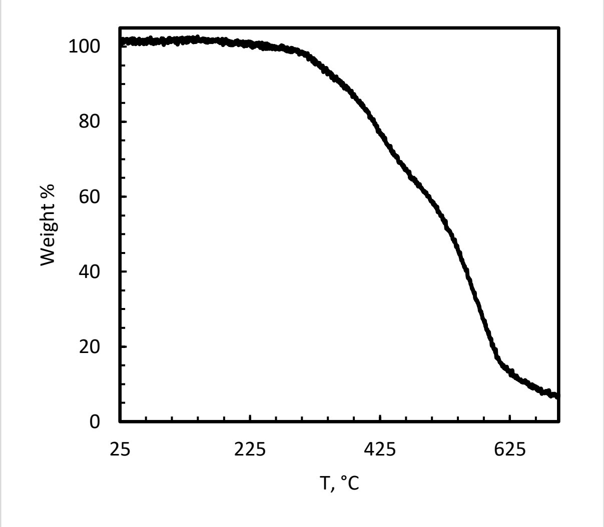

Figure S28. Mass loss as a function of temperature for sample corresponding to Entry 10 in Table S1.

Table S1, Entry 11
IR (ATR-FTIR, cm^{-1}): ν 3400w (O-H), 2924w (C-H), 1676s (C=O), 1604m (C=C), 1510w, 1402m, 1222m, 1213s, 983m, 818s.
TGA: T_d = 335 °C

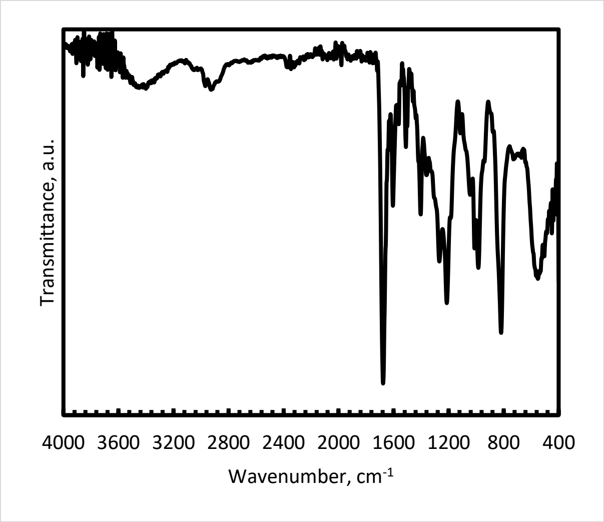

Figure S29. Infrared spectrum (ATR-FTIR) of the sample corresponding to Entry 11 in Table S1.

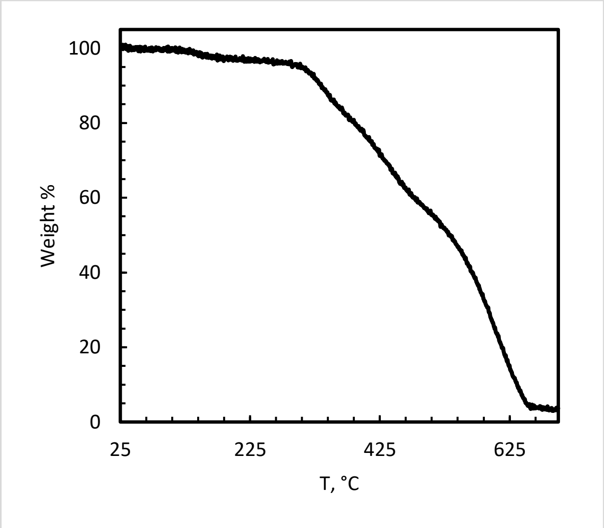

Figure S30. Mass loss as a function of temperature for sample corresponding to Entry 11 in Table S1.

Table S1, Entry 12
IR (ATR-FTIR, cm^{-1}): ν 3389w (O-H), 2907w (C-H), 1676s (C=O), 1604m (C=C), 1508w, 1402m, 1225m, 1211s, 983m, 818s.
TGA: T_d = 328 °C

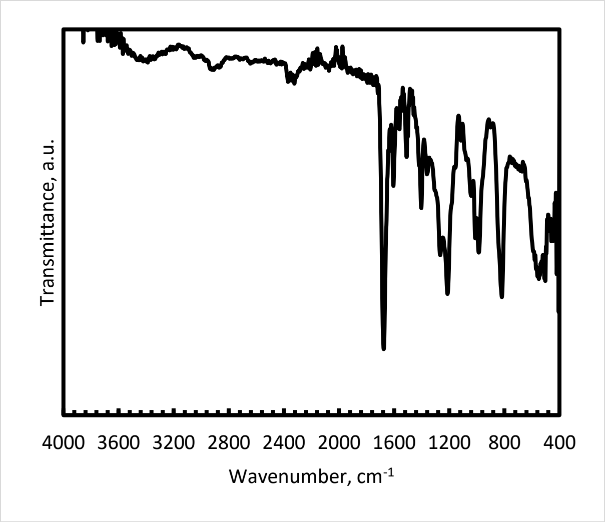

Figure S31. Infrared spectrum (ATR-FTIR) of the sample corresponding to Entry 12 in Table S1.

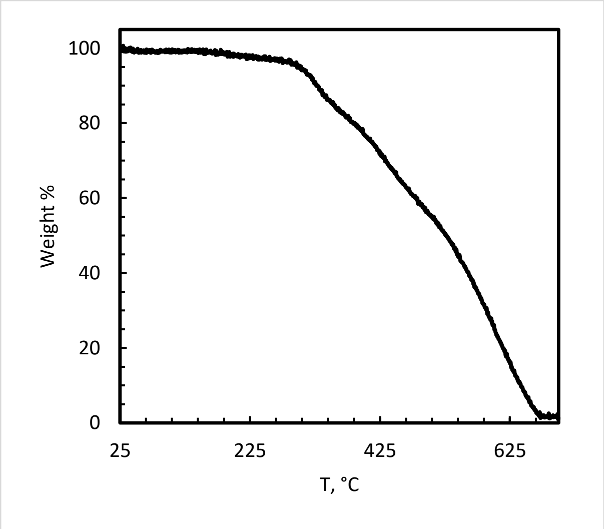

Figure S32. Mass loss as a function of temperature for sample corresponding to Entry 12 in Table S1.

Table S1, Entry 13
IR (ATR-FTIR, cm^{-1}): ν 3401w (O-H), 2926w (C-H), 1676s (C=O), 1605m (C=C), 1510w, 1404m, 1225m, 1213s, 982s, 818s, 555w.
TGA: T_d = 352 °C

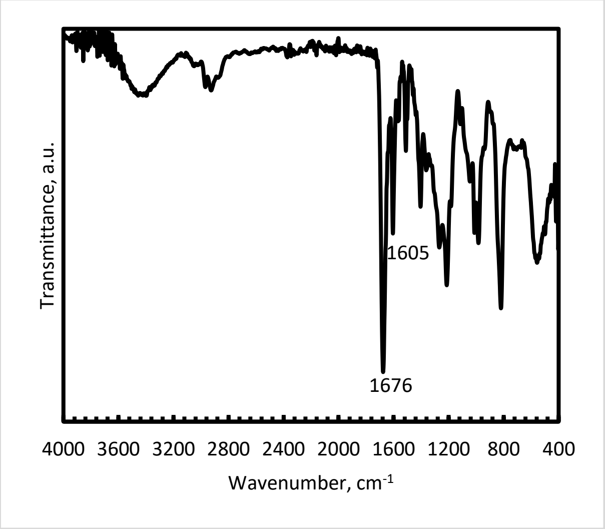

Figure S33. Infrared spectrum (ATR-FTIR) of the sample corresponding to Entry 13 in Table S1.

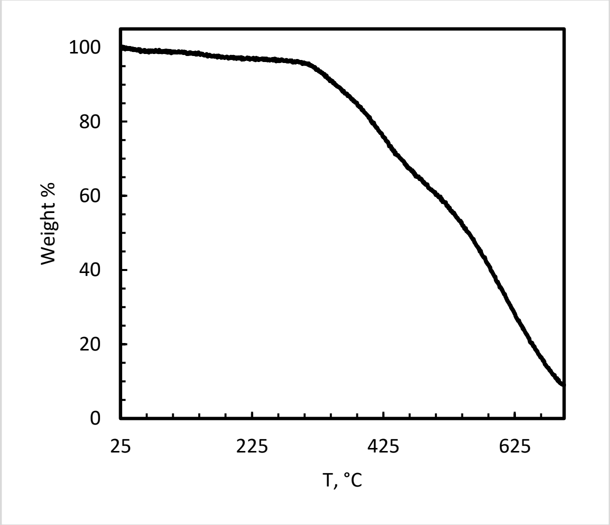

Figure S34. Mass loss as a function of temperature for sample corresponding to Entry 13 in Table S1.

Table S1, Entry 14
IR (ATR-FTIR, cm^{-1}): ν 3427w (O-H), 2918w (C-H), 1676s (C=O), 1601m (C=C), 1506w, 1404m, 1228s, 1211s, 982m, 820s, 542m.
TGA: T_d = 342 °C

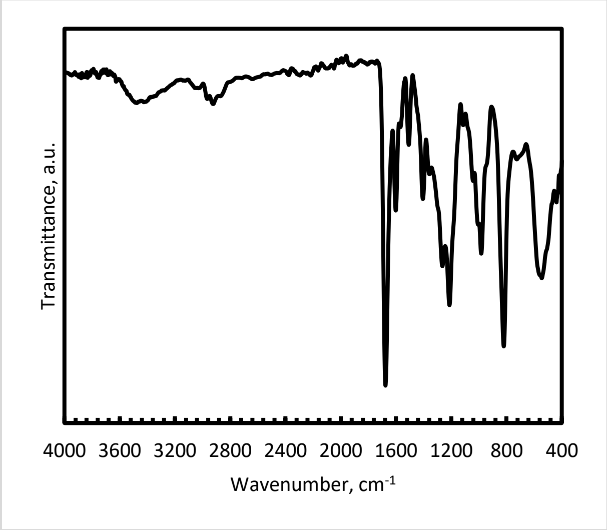

Figure S35. Infrared spectrum (ATR-FTIR) of the sample corresponding to Entry 14 in Table S1.

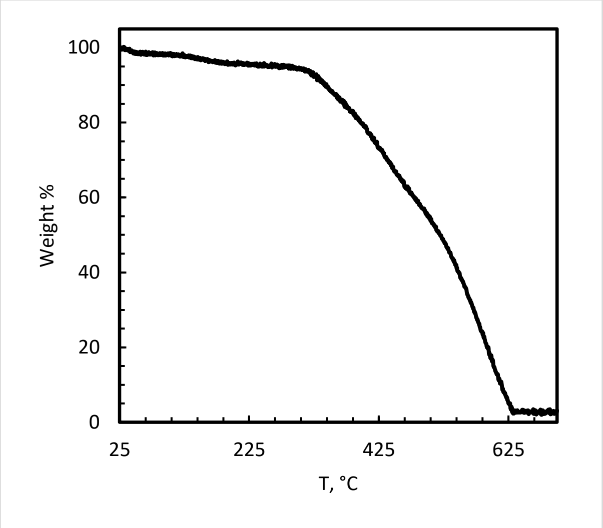

Figure S36. Mass loss as a function of temperature for sample corresponding to Entry 14 in Table S1.

Table S1, Entry 15
IR (ATR-FTIR, cm^{-1}): ν 3445w (O-H), 2918w (C-H), 1678s (C=O), 1605m (C=C), 1510w, 1402m, 1263m, 1213s, 981s, 818s, 550m.
TGA: T_d = 363 °C

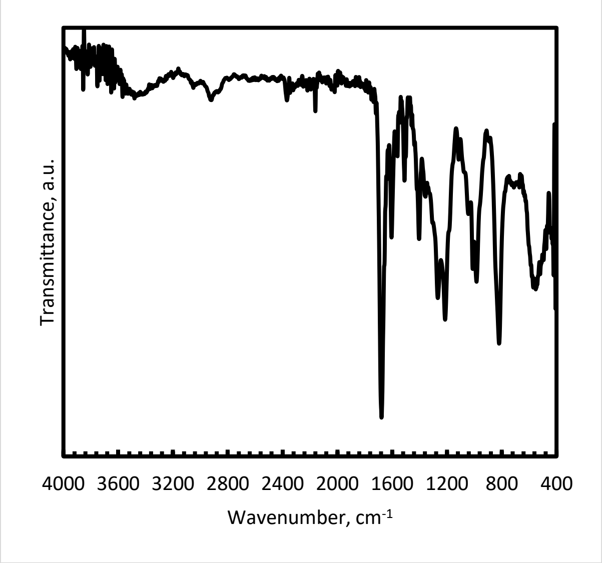

Figure S37. Infrared spectrum (ATR-FTIR) of the sample corresponding to Entry 15 in Table S1.

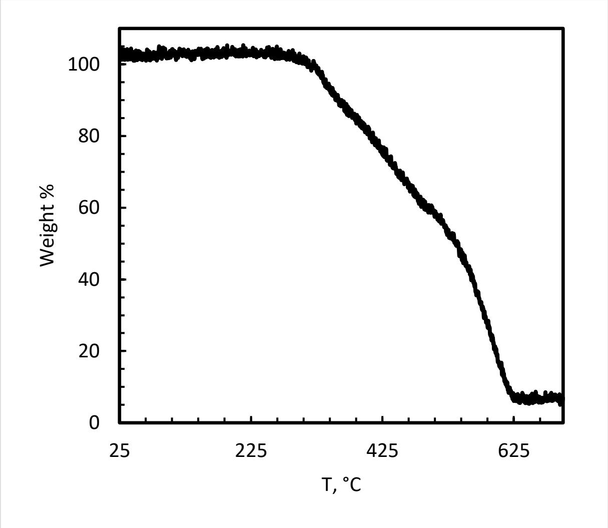

Figure S38. Mass loss as a function of temperature for sample corresponding to Entry 15 in Table S1.

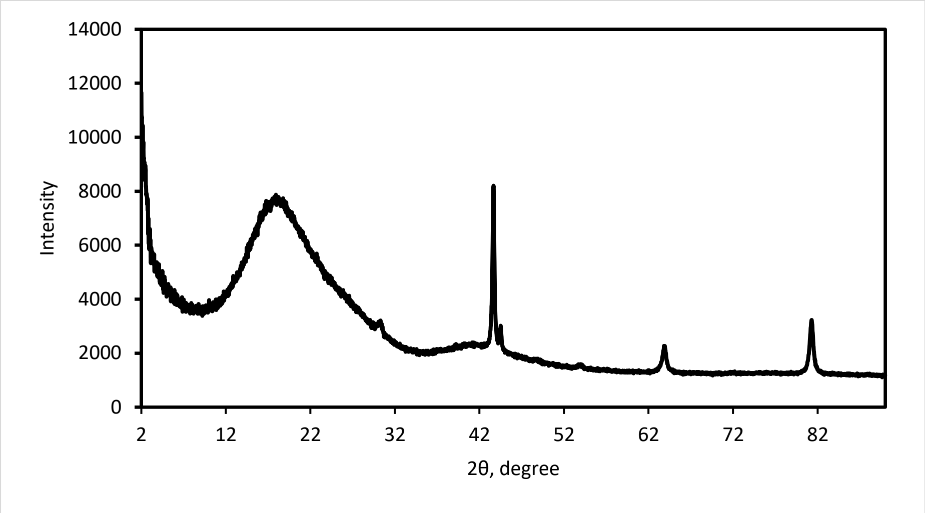

Figure S39. Experimental powder XRD patterns of the sample corresponding to Entry 15 in Table S1. Crystalline peaks are from Teflon substrate.

<u>Table S1, Entry 16</u>
IR (ATR-FTIR, cm^{-1}): ν 3404w (O-H), 2911w (C-H), 1676s (C=O), 1603m (C=C), 1508w, 1402m, 1267s, 1211s, 984m, 818s, 555m.
TGA: T_d = 343 °C

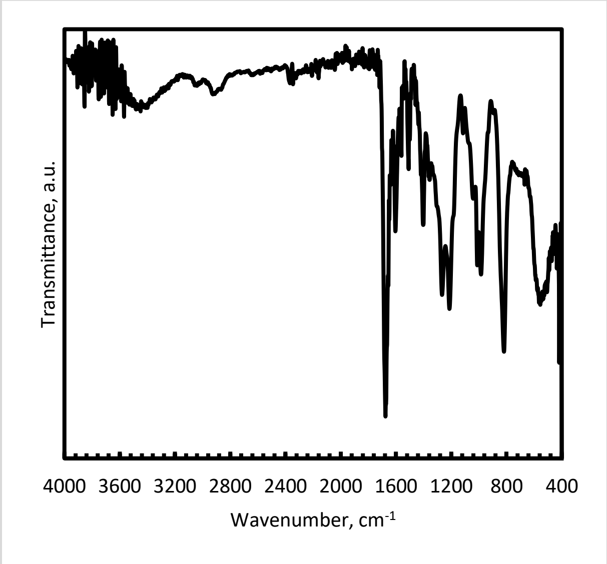

Figure S40. Infrared spectrum (ATR-FTIR) of the sample corresponding to Entry 16 in Table S1.

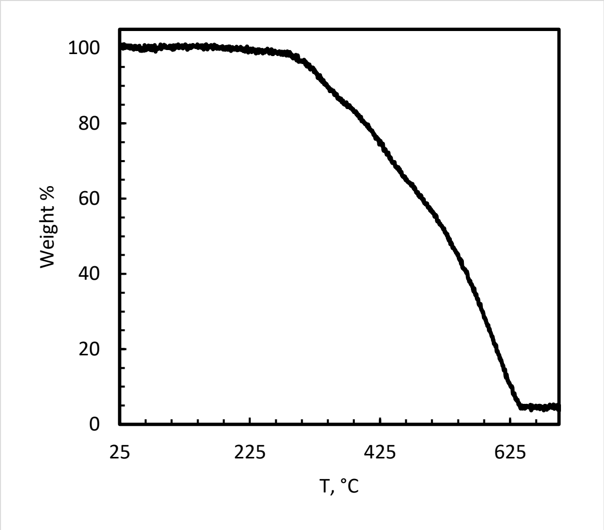

Figure S41. Mass loss as a function of temperature for sample corresponding to Entry 16 in Table S1.

Table S1, Entry 17
IR (ATR-FTIR, cm^{-1}): ν 3449w (O-H), 2914w (C-H), 1676s (C=O), 1604m (C=C), 1510w, 1402m, 1267s, 1213s, 983m, 818s, 548m.
TGA: T_d = 344 °C

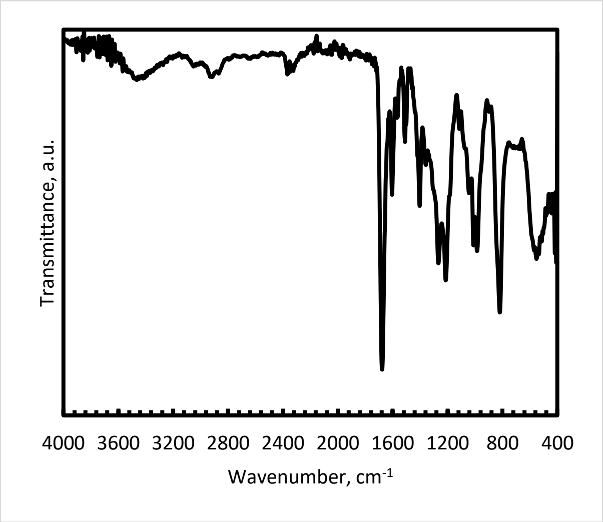

Figure S42. Infrared spectrum (ATR-FTIR) of the sample corresponding to Entry 17 in Table S1.

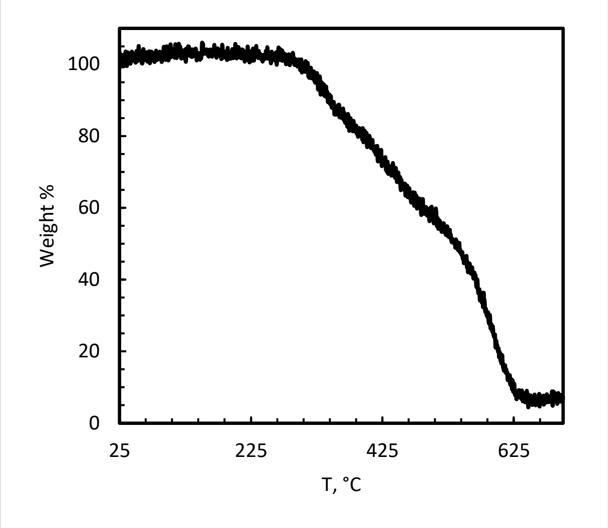

Figure S43. Mass loss as a function of temperature for sample corresponding to Entry 17 in Table S1.

Table S1, Entry 18

IR (ATR-FTIR, cm^{-1}): ν 3424w (O-H), 2924w (C-H), 1672s (C=O), 1599s (C=C), 1287m, 1267s, 1015m, 824s.

TGA: T_d = 396 °C

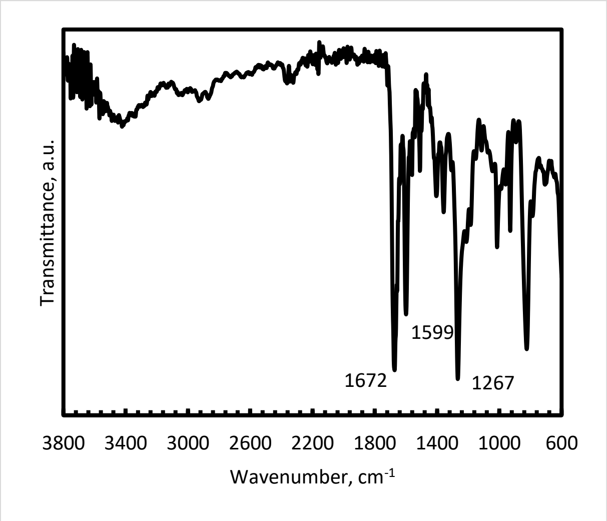

Figure S44. Infrared spectrum (ATR-FTIR) of the sample corresponding to Entry 18 in Table S1.

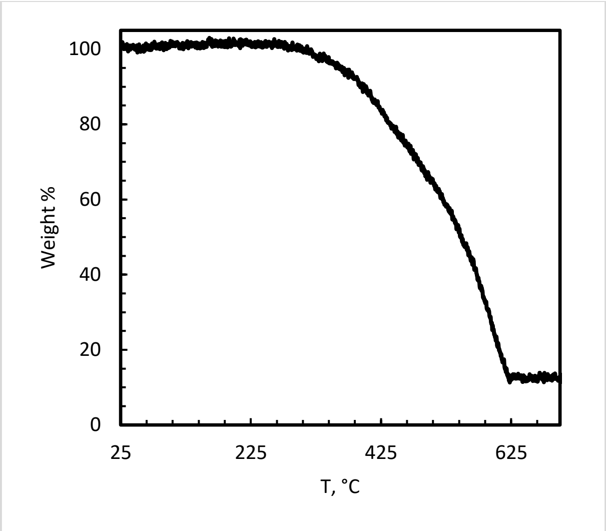

Figure S45. Mass loss as a function of temperature for sample corresponding to Entry 18 in Table S1.

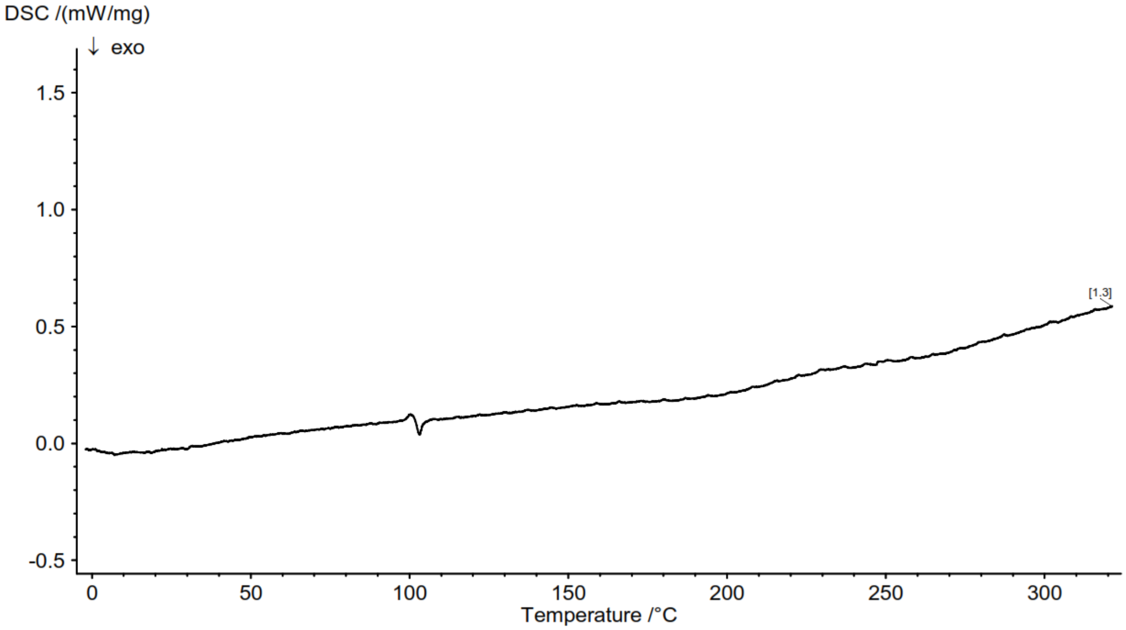

Figure S46. DSC trace corresponding to Entry 18 in Table S1.

<u>Table S1, Entry 19</u>

A 100 mL ampoule equipped with a J-Young's valve was refilled wit argon, charged with pre-catalyst **1** (2.5 mg, 0.005 mmol, 1 mol%), 5 mL of dry THF and solution of KO*t*Bu (0.5 mL of 0.01 M in THF, 1 mol%). The mixture was stirred at room temperature for 1 h and then the volatiles were evaporated. To the resulting mixture 1,4-benzenedimethanol (69 mg, 0.5 mmol) and 1,4-diacetylketone (81 mg, 0.5 mmol) were added. *tert*-Amyl alcohol was added and the flask was sealed under an argon atmosphere before heating to 140 °C for 18 hours. To the resulting mixture, 5 mL of 1 M HCl was added and the flask had been heated at 90 °C for 1 h.

The isolated amount was not enough to perform analysis.

<u>Table S1, Entry 20</u>

A 100 mL ampoule equipped with a J-Young's valve was refilled wit argon, charged with pre-catalyst **1** (2.5 mg, 0.005 mmol, 1 mol%), 5 mL of dry THF and solution of KO*t*Bu (1.0 mL of 0.01 M in THF, 2 mol%).

The mixture was stirred at room temperature for 1 h and then the volatiles were evaporated. To the resulting mixture 1,4-benzenedimethanol (69 mg, 0.5 mmol) and 1,4-diacetylketone (81 mg, 0.5 mmol) were added. *tert*-Amyl alcohol was added and the flask was sealed under an argon atmosphere before heating to 140 °C for 18 hours. To the resulting mixture, 5 mL of 1 M HCl was added and the flask had been heated at 90 °C for 1 h.
The isolated amount was not enough to perform analysis.

<u>Table S1, Entry 21</u>
The isolated amount was not enough to perform analysis.

<u>Table S1, Entry 22</u>
The isolated amount was not enough to perform analysis.

1.4 NMR spectra of mother liquor from coupling reaction of 1,4-diacetylbenzene and 1,4-benzenedimethanol as described in Table S1.

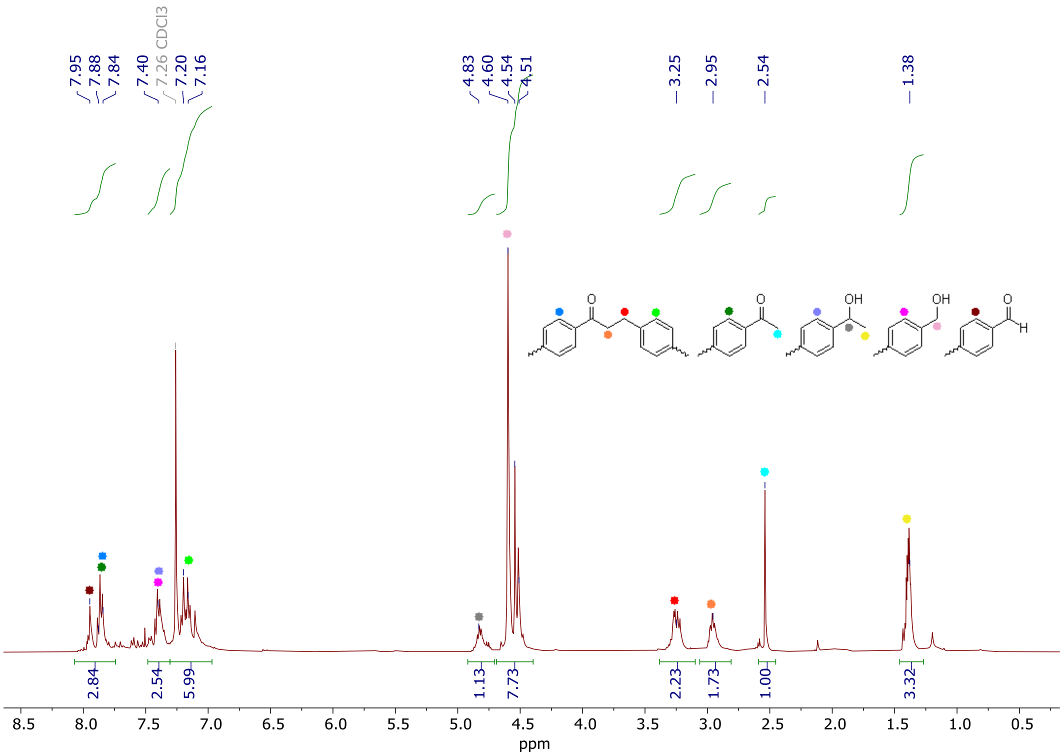

Figure S47. ^{1}H NMR spectrum of mother liquor after polymer precipitation from the reaction Table S1; Entry 7 in $CDCl_3/CD_3OD$ = 3/1 at room temperature.

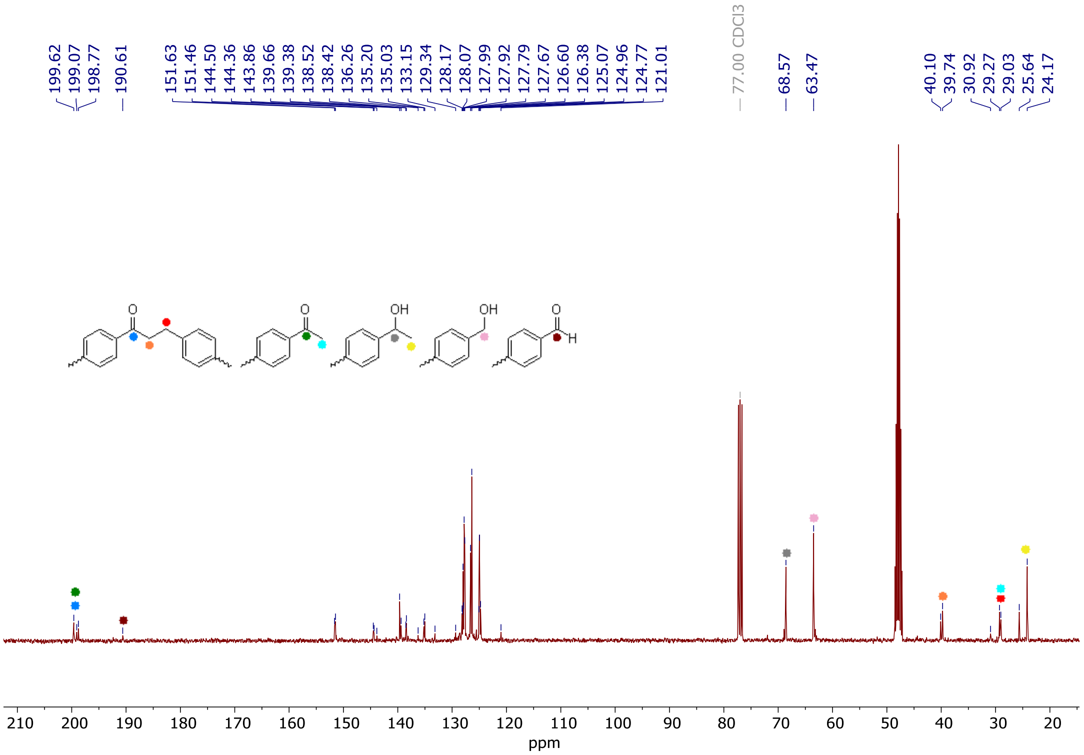

Figure S48. $^{13}C\{^{1}H\}$ NMR spectrum of mother liquor after polymer precipitation from the reaction Entry 7 in Table S1 in $CDCl_3/CD_3OD$ = 3/1 at room temperature.

1.5 Synthesis of polyketones from diketones and diols.

General method for the coupling of diketones and 1,4-benzenedimethanol, 1,3-benzenedimethanol and 1,4-cyclohexanedimethanol:
A 100 mL ampoule equipped with a J-Young's valve was charged with pre-catalyst **1** (2.5 mg, 0.005 mmol, 1 mol%) and Cs_2CO_3 (16.5 mg, 0.05 mmol, 10 mol%) or KO*t*Bu (56 mg, 0.5 mmol, 100 mol%), diol (0.5 mmol) and diketone (0.5 mmol). The flask was sealed under an argon atmosphere and *tert*-amyl alcohol (5 mL) was added before heating to 140 °C for 2 or 18 h with stirring. After this period, the reaction vessel was allowed to cool to room temperature and any gas evolved (presumably H_2) during the reaction was measured when possible. To the resulting mixture 5 mL of 1 M HCl were added and the flask had been heated at 90 °C for 1 h. The precipitate was filtered and dried under reduced pressure at 120 °C.

PAAK-1 (corresponds to Table S1, entry 15)

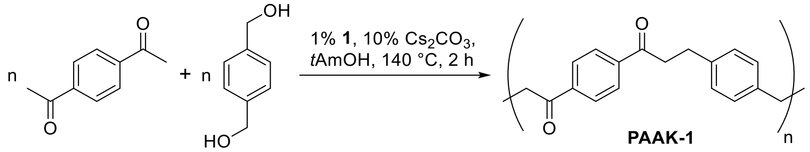

1,4-Dimethanolbenzene (69 mg, 0.5 mmol) and 1,4-diacetylbenzene (81 mg, 0.5 mmol) were used. The polymer was obtained in 89% yield (117 mg) as a yellow solid.
IR (ATR-FTIR, cm^{-1}): ν 3445w (O-H), 2918w (C-H), 1678s (C=O), 1605m (C=C), 1510w, 1402m, 1263m, 1213s, 981s, 818s, 550m.
TGA: T_d = 363 °C
GPC: bimodal: MW(1) = 1.5 kDa, PDI = 1.3; MW(2) = 51.1 kDa, PDI = 1.6.

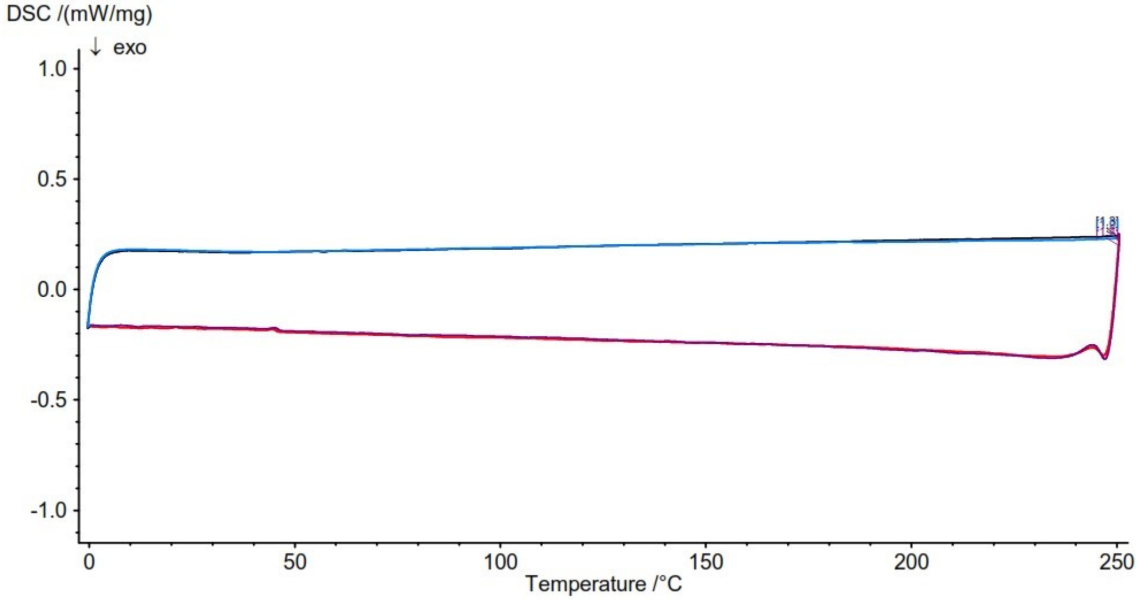

Figure S49. DSC trace corresponding to **PAAK-1**.

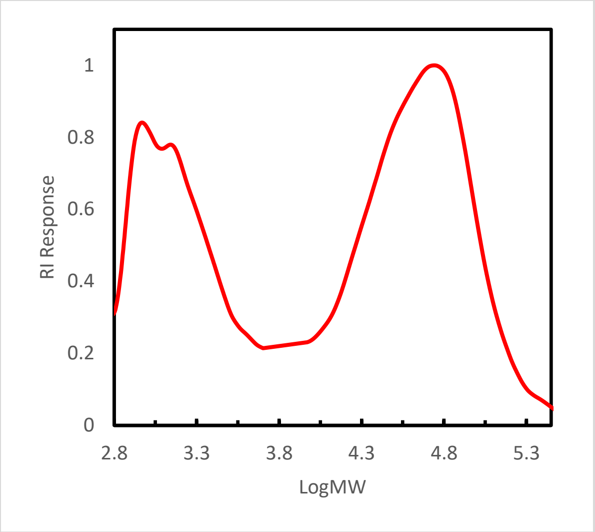

Figure S50. GPC chromatograph corresponding to **PAAK-1**.

PAAK-2

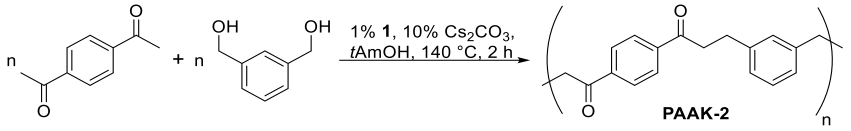

1,3-benznenedimethanol (69 mg, 0.5 mmol) and 1,4-diacetylbenzene (81 mg, 0.5 mmol) were used. The polymer was obtained with 77% yield (101 mg) as a yellow solid.
^{13}C CP MAS NMR (100.6 MHz): δ 199.4, 151.3, 141.1, 128.2, 70.8, 64.5, 41.1, 36.2, 28.8.
IR (ATR-FTIR, cm^{-1}): ν 3422w (O-H), 2918w (C-H), 1678s (C=O), 1605m (C=C), 1449w, 1402m, 1265m, 1211s, 984s, 793m, 704s.
TGA: T_d = 351 °C
GPC: bimodal: MW(1) = 1.2 kDa, PDI = 1.2; MW(2) = 54.1 kDa, PDI = 1.6.

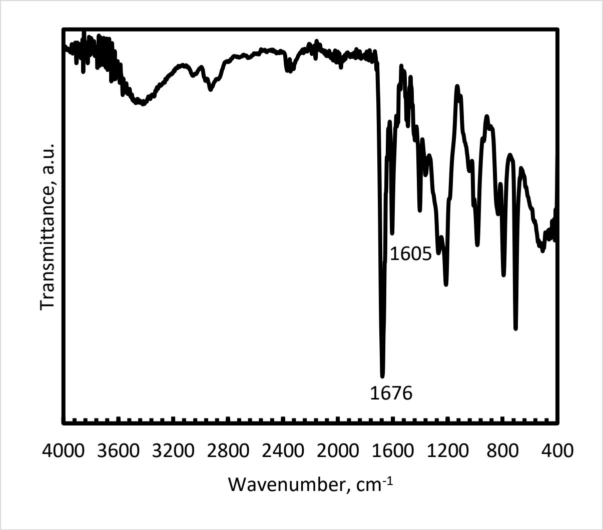

Figure S51. Infrared spectrum (ATR-FTIR) of polyketone **PAAK-2**.

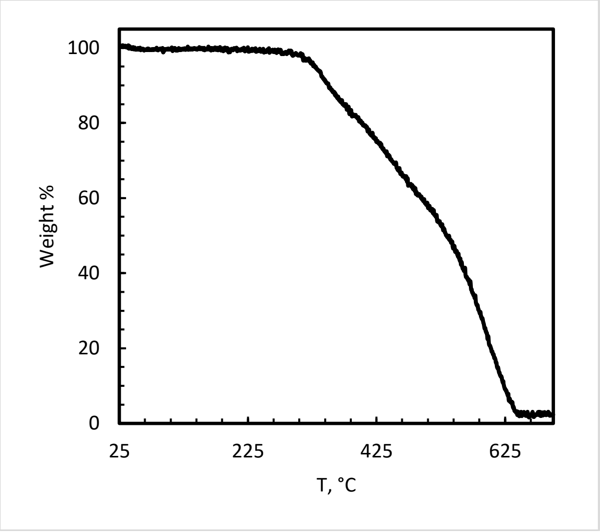

Figure S52. Mass loss as a function of temperature for polyketone **PAAK-2**.

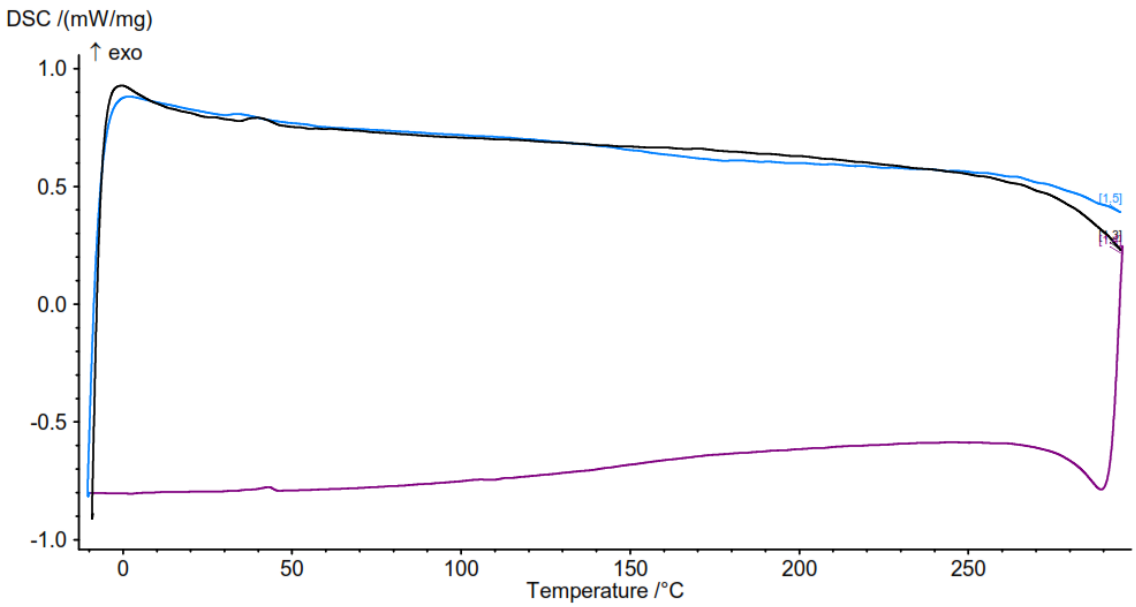

Figure S53. DSC trace corresponding to **PAAK-2**.

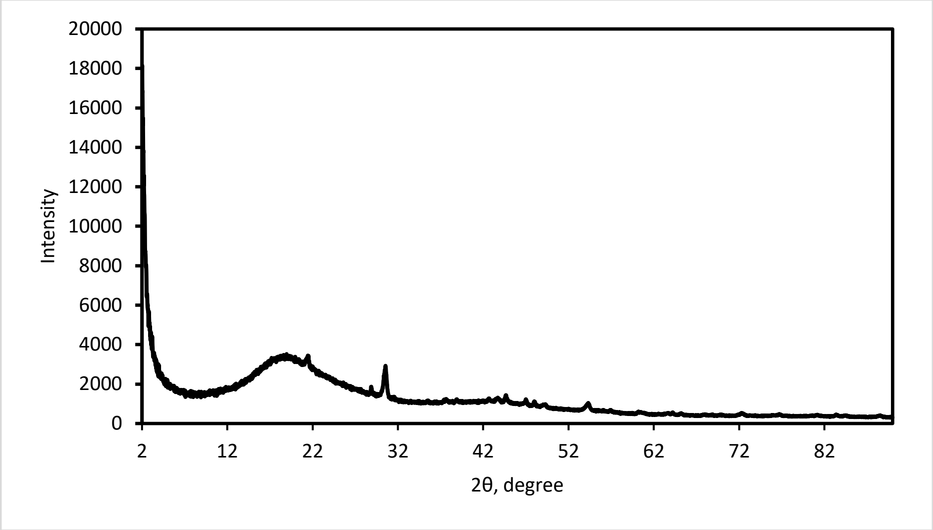

Figure S54. Experimental powder XRD patterns of **PAAK-2**. Crystalline peaks were indexed in the small-volume unit cell which remains unidentified.

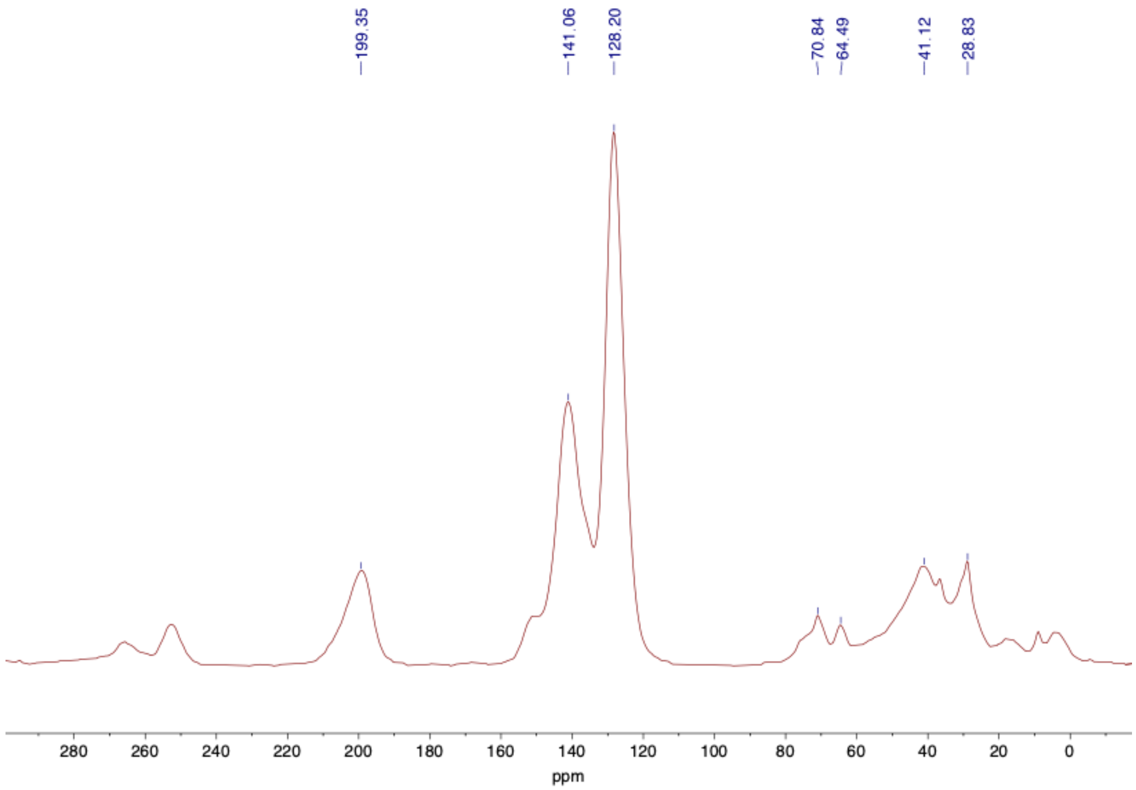

Figure S55. ^{13}C CP MAS NMR spectrum of **PAAK-2**.

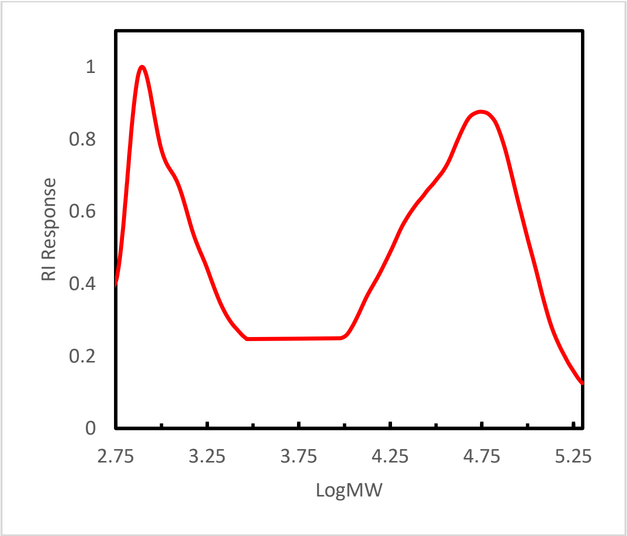

Figure S56. GPC chromatograph corresponding to **PAAK-2**.

PAAK-3

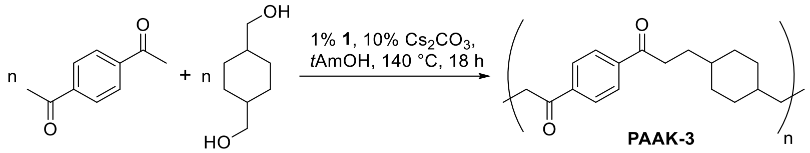

1,4-Cyclohexanedimethanol (72 mg, 0.5 mmol) and 1,4-diacetylbenzene (81 mg, 0.5 mmol) were used. The polymer was obtained in 41% yield (55 mg) as a yellow solid.

^{13}C CP MAS NMR (100.6 MHz): δ 199.9, 148.1, 140.2, 128.4, 75.8, 68.4, 48.5, 36.7, 41.1, 30.7.

IR (ATR-FTIR, cm^{-1}): ν 3482w (O-H), 2918m (C-H), 2853m (C-H), 1676s (C=O), 1605m (C=C), 1402m, 1269m, 1217m, 1011m, 829m, 503m.

TGA: T_d = 369 °C

GPC: bimodal: MW(1) = 1.6 kDa, PDI = 1.3; MW(2) = 52.9 kDa, PDI = 1.7.

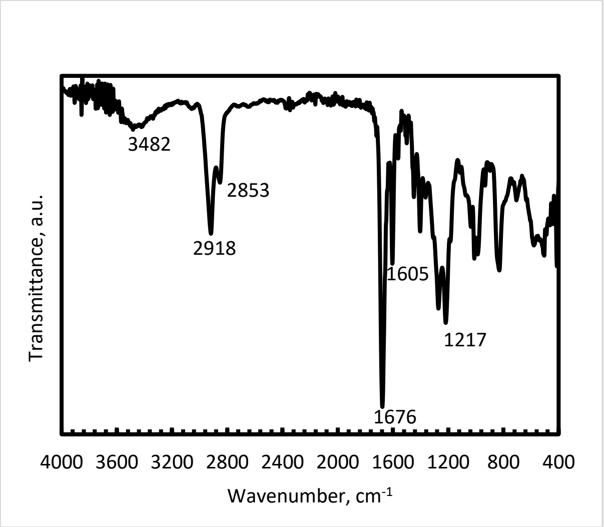

Figure S57. Infrared spectrum (ATR-FTIR) of polyketone **PAAK-3**.

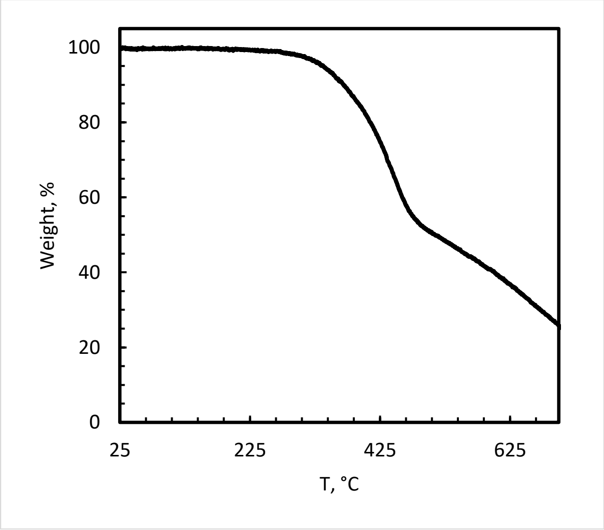

Figure S58. Mass loss as a function of temperature for polyketone **PAAK-3.**

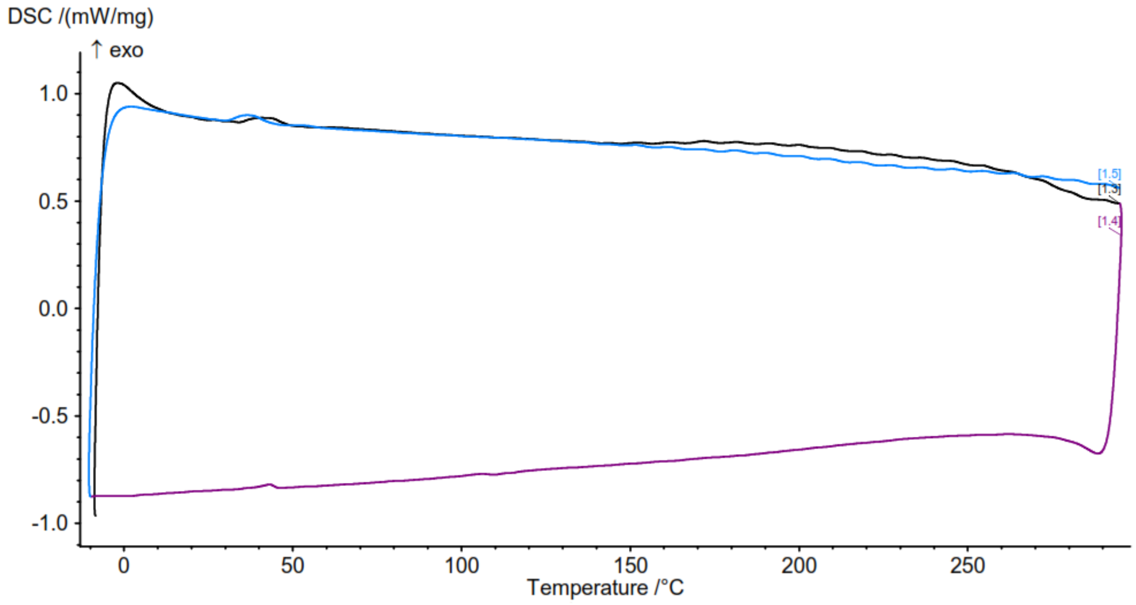

Figure S59. DSC trace corresponding to **PAAK-3**.

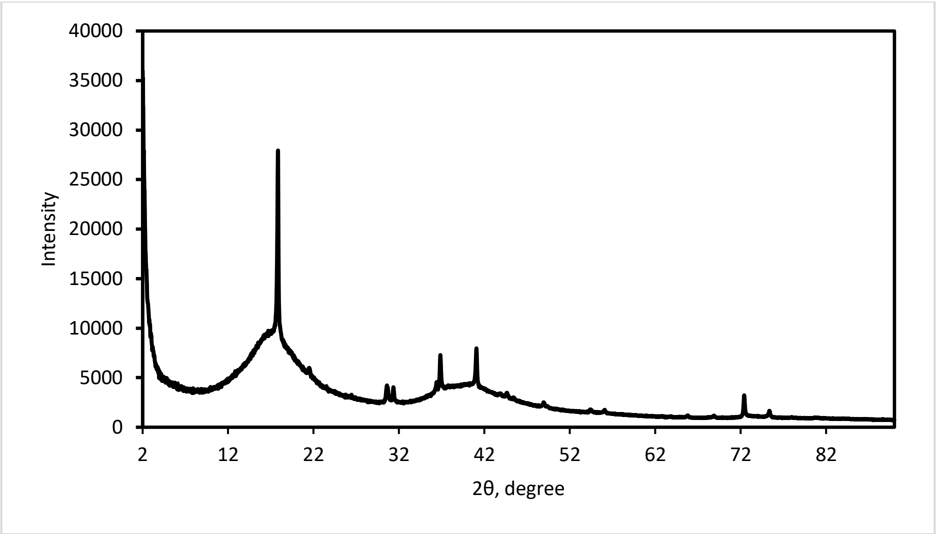

Figure S60. Experimental powder XRD patterns of **PAAK-3**. Unidentifiable crystalline phases are present.

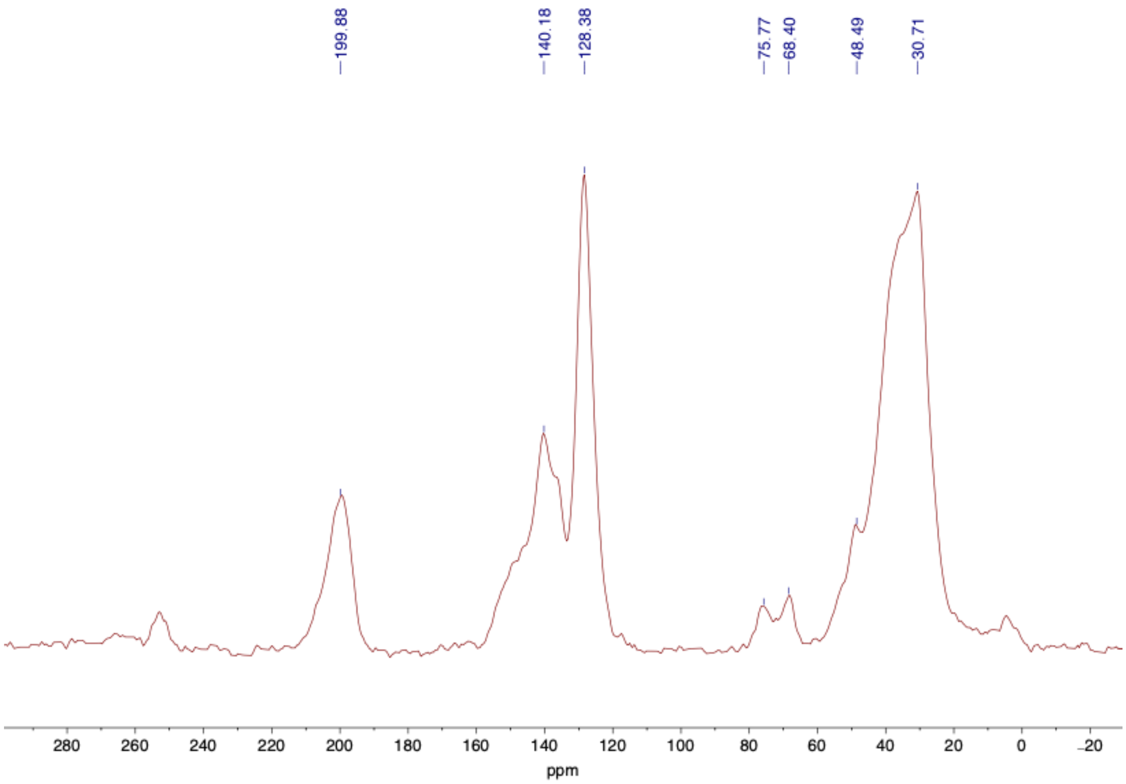

Figure S61. ^{13}C CP MAS NMR spectrum of **PAAK-3**.

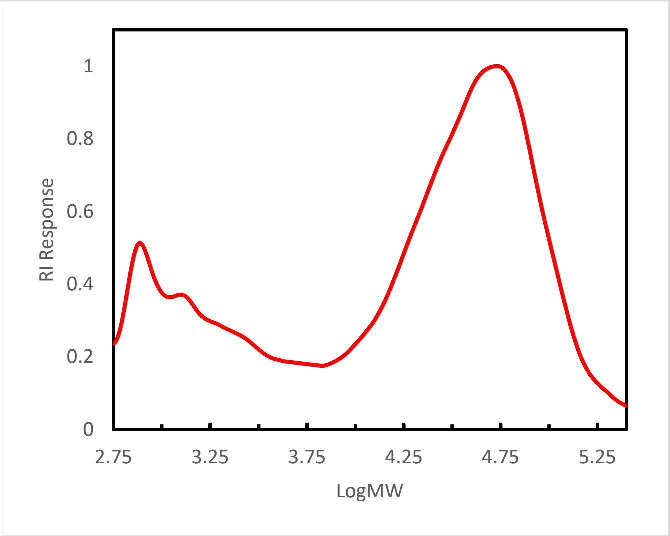

Figure S62. GPC chromatograph corresponding to **PAAK-3**.

PAAK-4

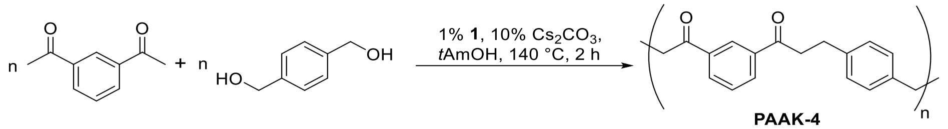

1,4-Benzenedimethanol (69 mg, 0.5 mmol) and 1,3-diacetylbenzene (81 mg, 0.5 mmol) were used. The polymer was obtained in 98 % yield (129 mg) as a yellow solid.
^{13}C CP MAS NMR (100.6 MHz): δ 199.5, 141.0, 137.9, 128.6, 75.1, 71.0, 64.5, 41.2, 37.2, 29.1.
IR (ATR-FTIR, cm^{-1}): ν 3472w (O-H), 3059w, 2920w (C-H), 1680s (C=O), 1597m, 1171m, 999w, 795m, 704m.
TGA: T_d = 350 °C
GPC: bimodal: MW(1) = 1.4 kDa, PDI = 1.3; MW(2) = 51.1 kDa, PDI = 1.7.

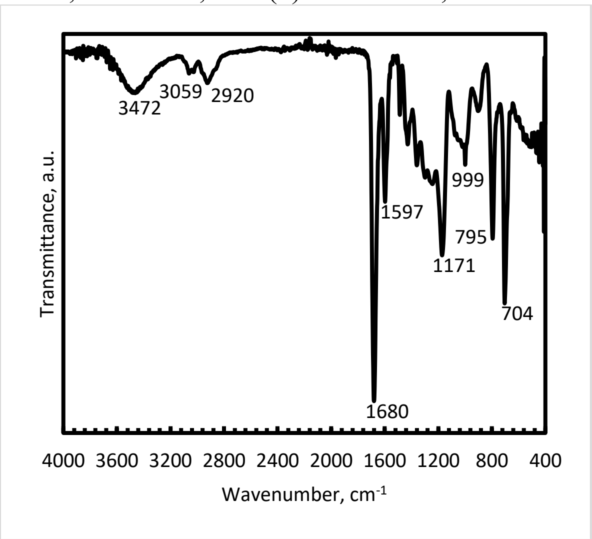

Figure S63. Infrared spectrum (ATR-FTIR) of polyketone **PAAK-4**.

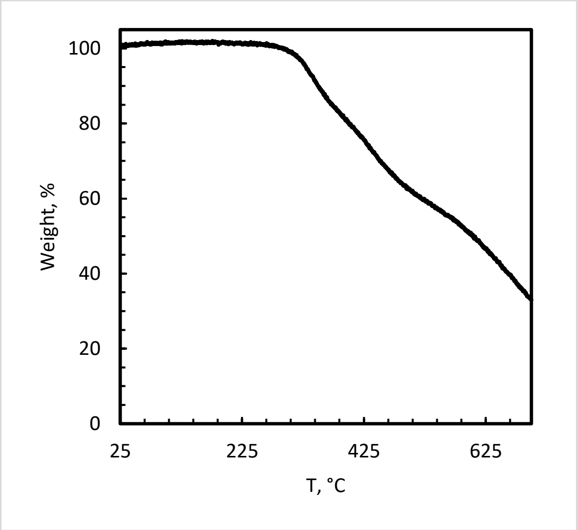

Figure S64. Mass loss as a function of temperature for polyketone **PAAK-4**.

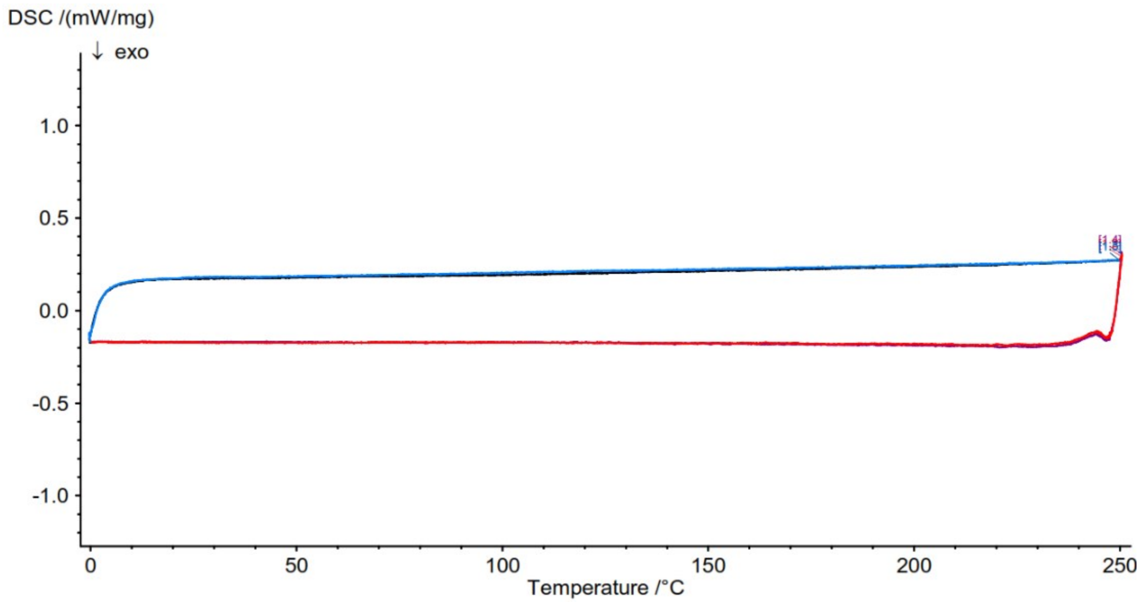

Figure S65. DSC trace corresponding to **PAAK-4**.

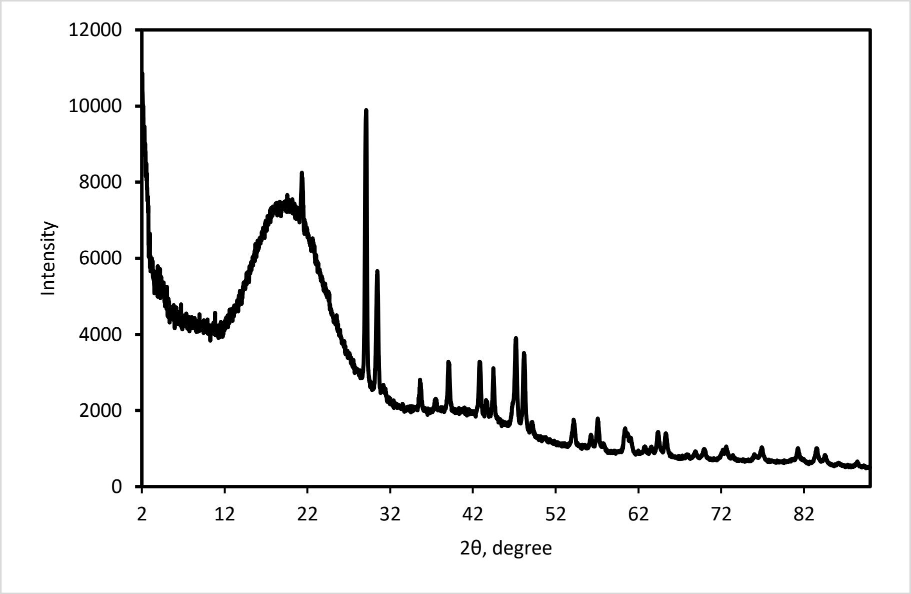

Figure S66. Experimental powder XRD patterns of **PAAK-4**. Unidentifiable crystalline phase present.

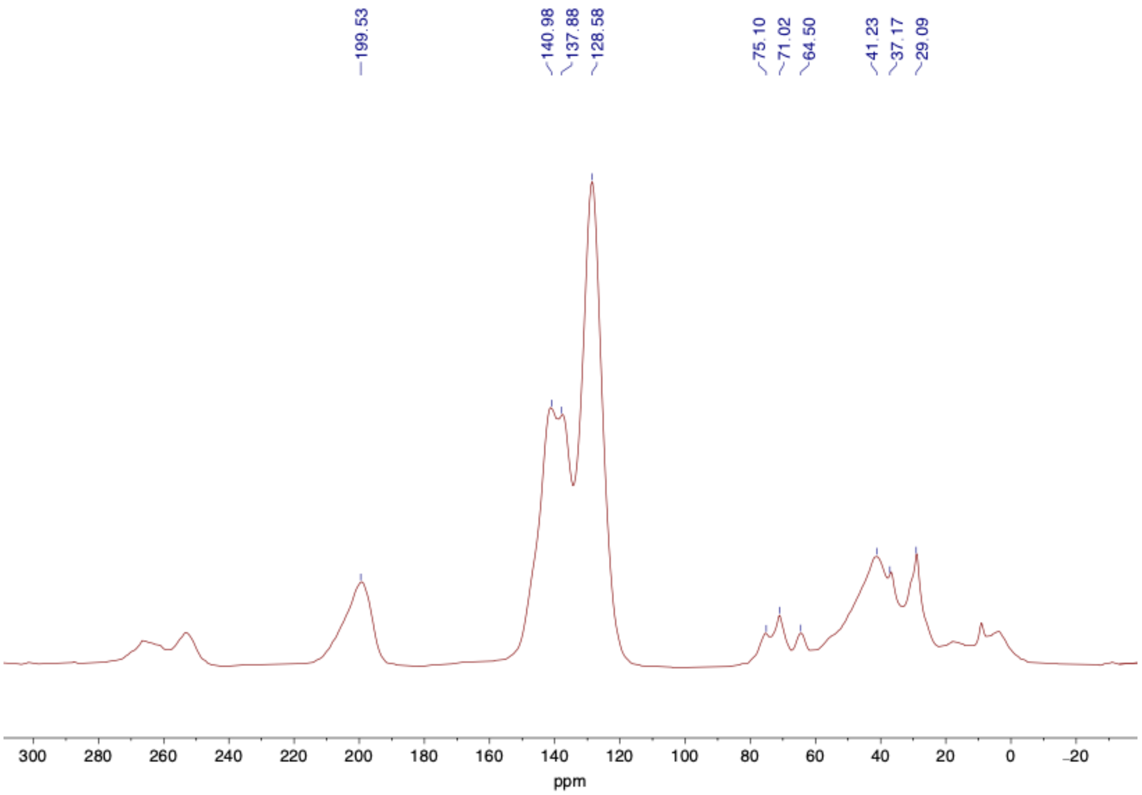

Figure S67. ^{13}C CP MAS NMR spectrum of **PAAK-4**.

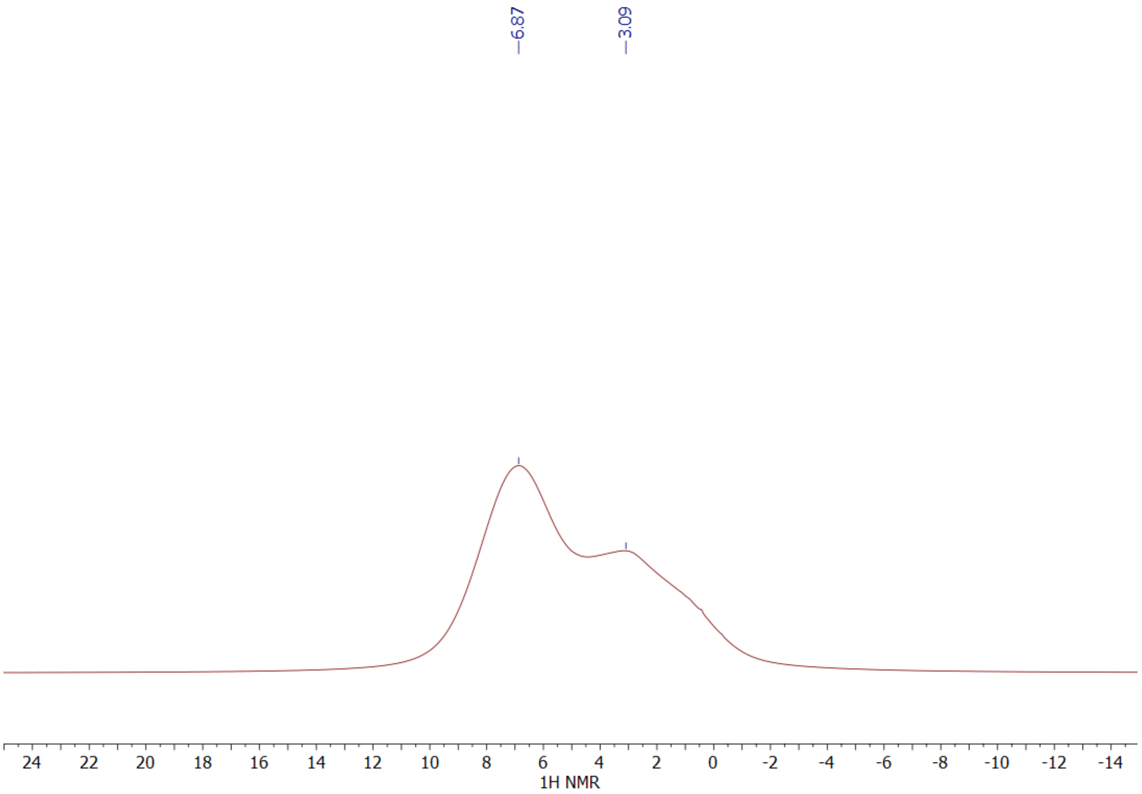

Figure S68. ^{1}H CP MAS NMR spectrum of **PAAK-4**.

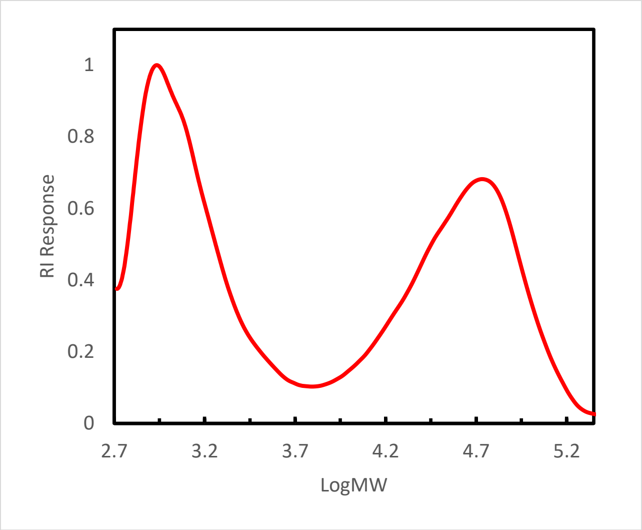

Figure S69. GPC chromatograph corresponding to **PAAK-4**.

PAAK-5

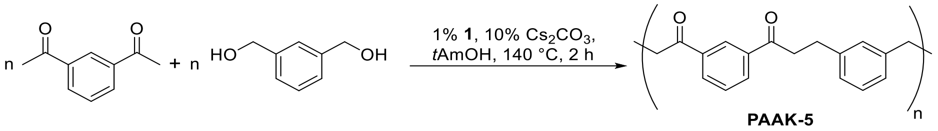

1,3-Benzenedimethanol (69 mg, 0.5 mmol) and 1,3-diacetylbenzene (81 mg, 0.5 mmol) were used. The polymer was obtained in 97 % yield (128 mg) as a yellow solid.
^{13}C CP MAS NMR (100.6 MHz): δ 199.5, 138.3, 128.9, 75.1, 70.9, 64.2, 41.4, 29.0.
IR (ATR-FTIR, cm^{-1}): ν 3422w (O-H), 3049w, 2914w (C-H), 1678s (C=O), 1597m, 1171m, 1016w, 804m, 696w, 554m.
TGA: T_d = 365 °C
GPC: bimodal: MW(1) = 2.1 kDa, PDI = 1.6; MW(2) = 53.7 kDa, PDI = 1.6.

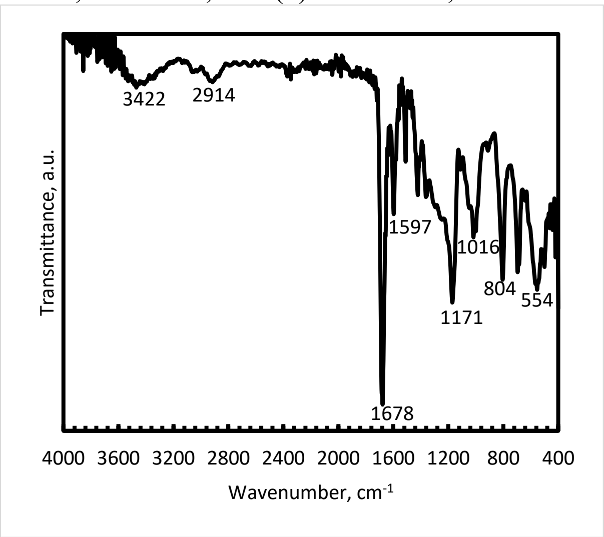

Figure S70. Infrared spectrum (ATR-FTIR) of polyketone **PAAK-5**.

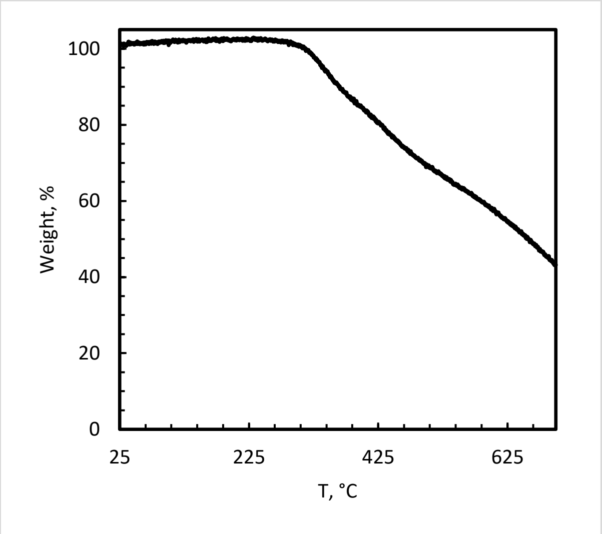

Figure S71. Mass loss as a function of temperature for polyketone **PAAK-5**.

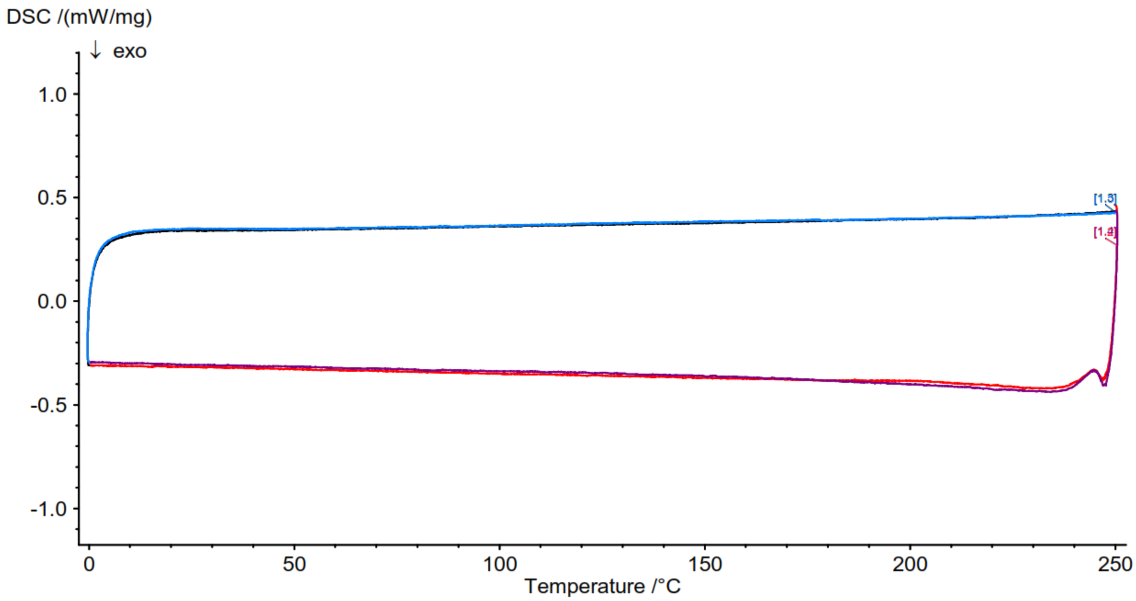

Figure S72. DSC trace corresponding to **PAAK-5**.

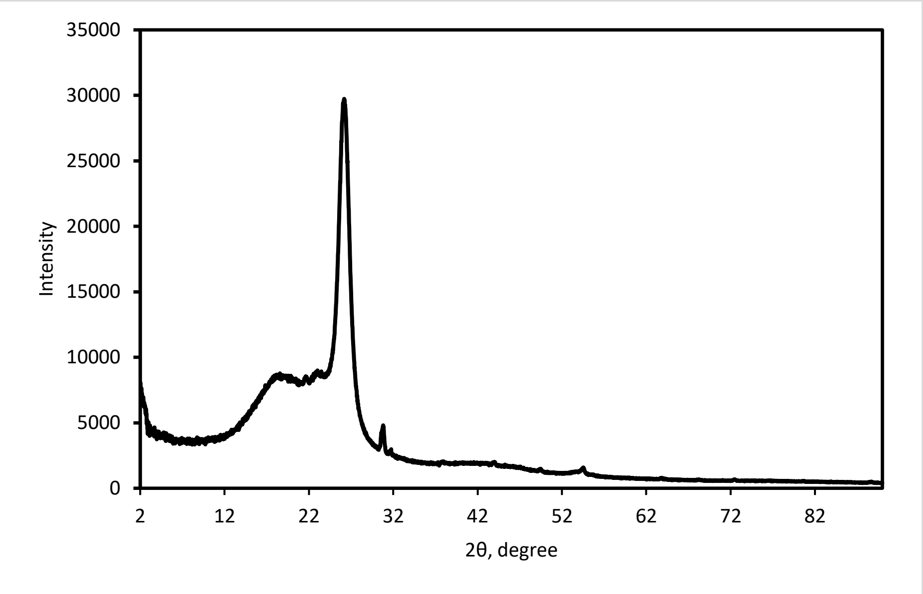

Figure S73. Experimental powder XRD patterns of **PAAK-5**. Crystalline peaks were indexed in the small-volume unit cell which remains unidentified. Tall thin amorphous halo is from polyester films used for measurements of small amount of sample.

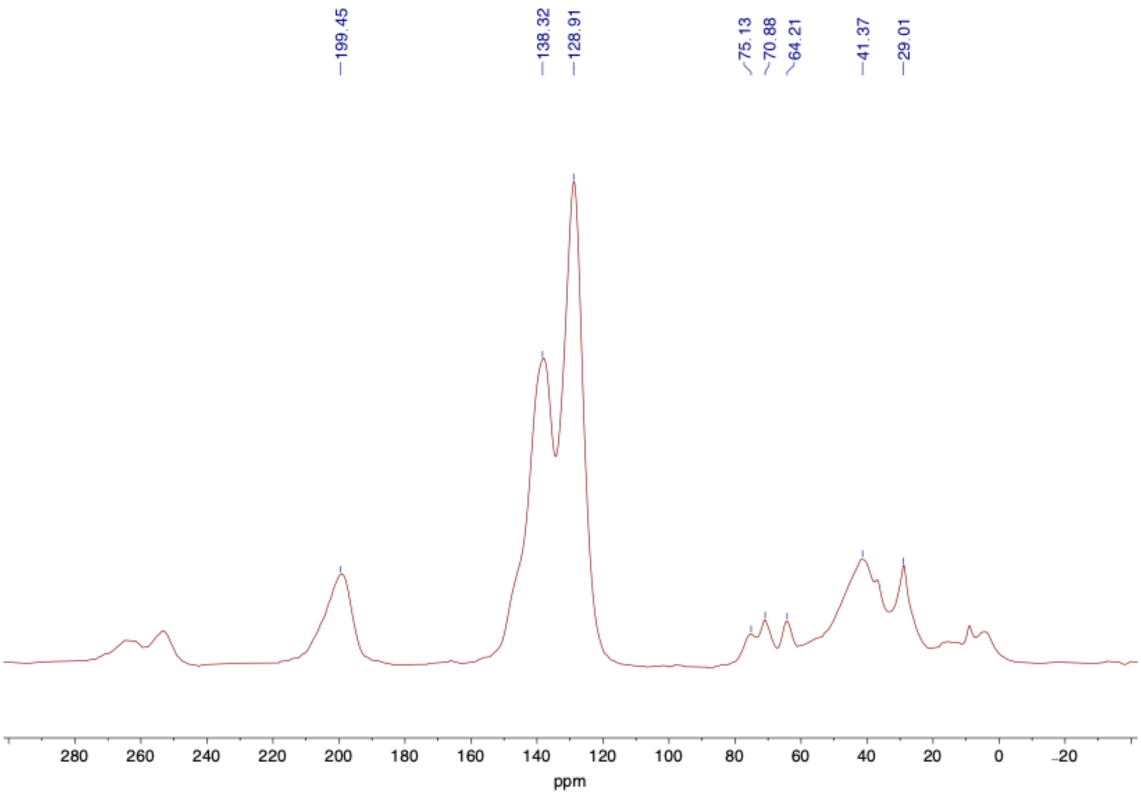

Figure S74. ^{13}C CP MAS NMR spectrum of **PAAK-5**.

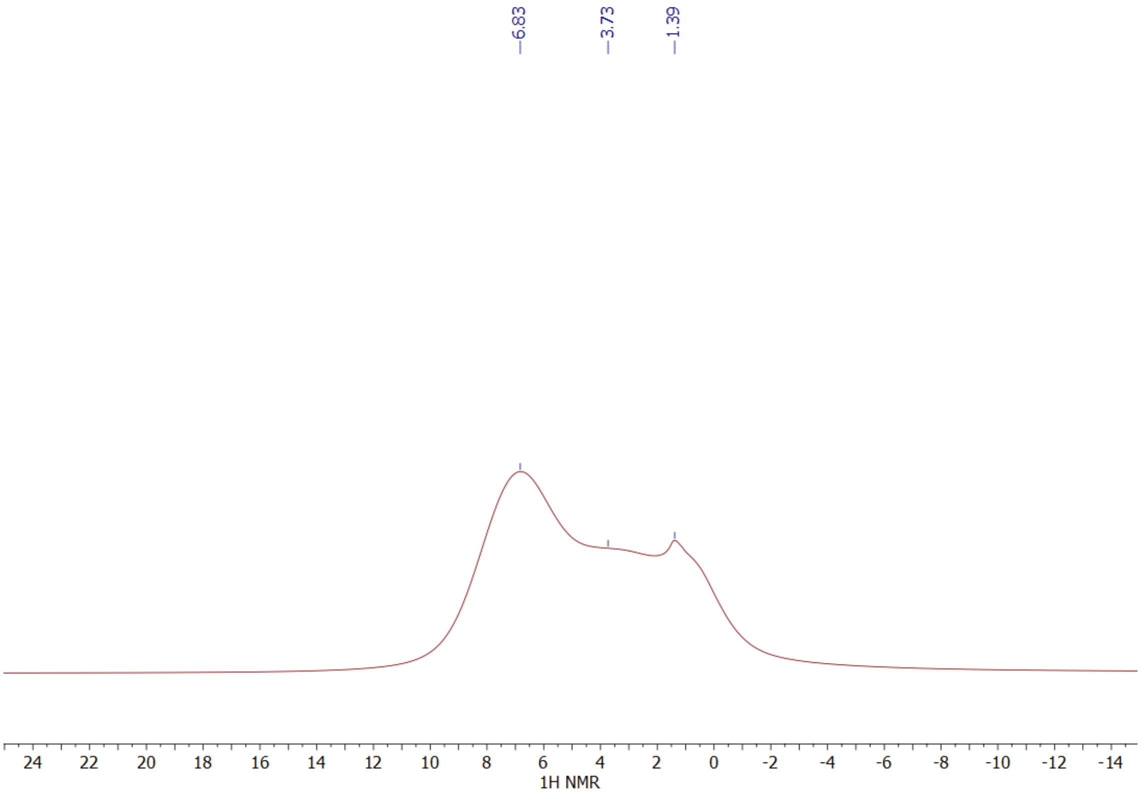

Figure S75. ^{1}H CP MAS NMR spectrum of **PAAK-5**.

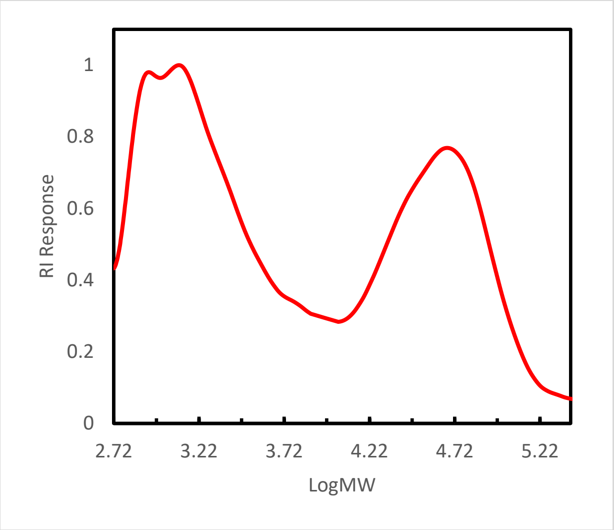

Figure S76. GPC chromatograph corresponding to **PAAK-5**.

PAAK-6

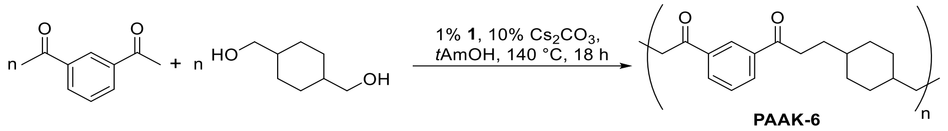

1,4-Cyclohexanedimethanol (72 mg, 0.5 mmol) and 1,3-diacetylbenzene (81 mg, 0.5 mmol) were used. The polymer was obtained in 99 % yield (134 mg) as a yellowish solid.
^{13}C CP MAS NMR (100.6 MHz): δ 199.9, 146.9, 137.5, 128.8, 74.4, 71.0, 47.9, 36.4, 34.0, 29.3.
IR (ATR-FTIR, cm^{-1}): ν 3485w (O-H), 2926s (C-H), 2851m (C-H), 1678s (C=O), 1597m, 1449m, 1269m, 1167s, 799m, 696s.
TGA: T_d = 380 °C
GPC: monomodal: MW(1) = 59.6 kDa, PDI = 1.6.

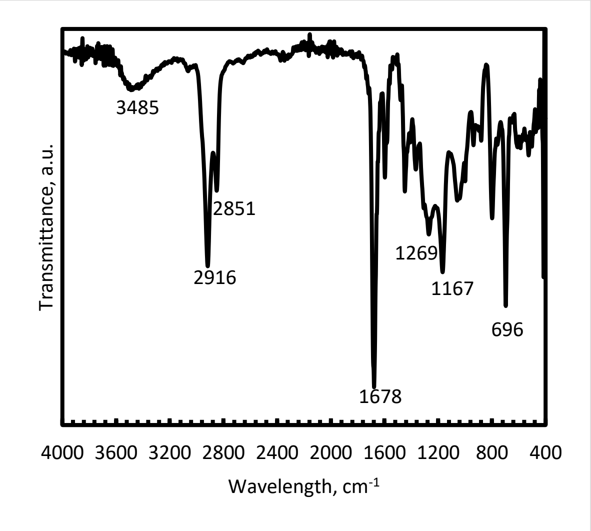

Figure S77. Infrared spectrum (ATR-FTIR) of polyketone **PAAK-6**.

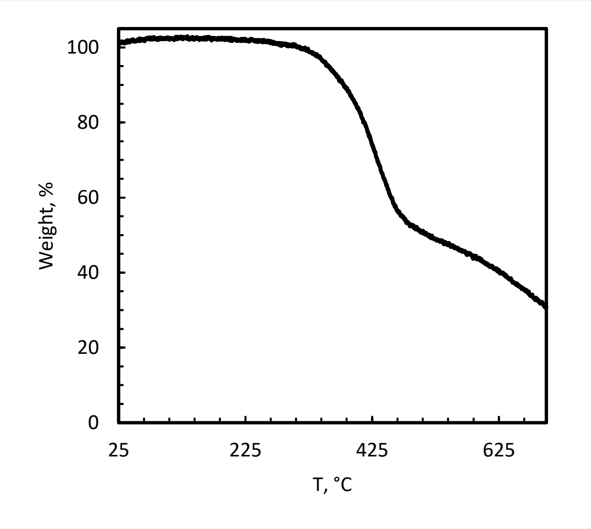

Figure S78. Mass loss as a function of temperature for polyketone **PAAK-6**.

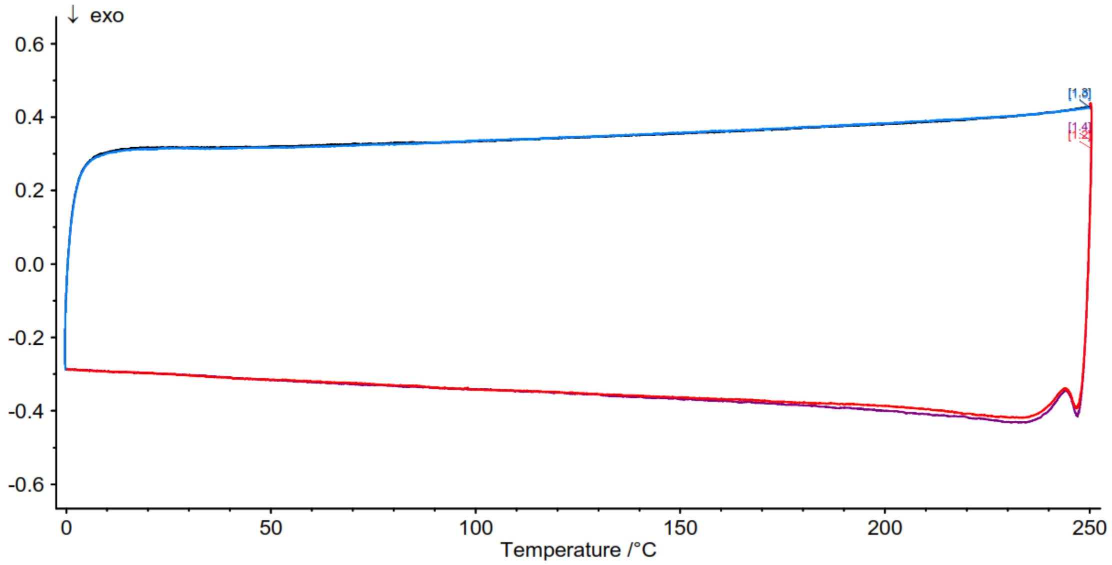

Figure S79. DSC trace corresponding to **PAAK-6**.

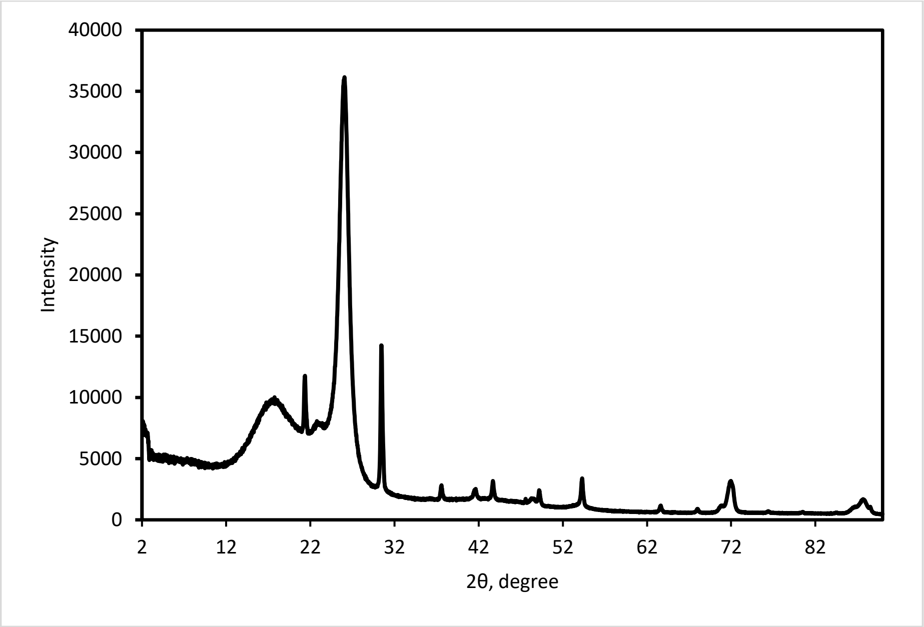

Figure S80. Experimental powder XRD patterns of **PAAK-6**. Crystalline peaks were indexed in the small-volume unit cell which remains unidentified. Tall thin amorphous halo is from polyester films used for measurements of small amount of sample.

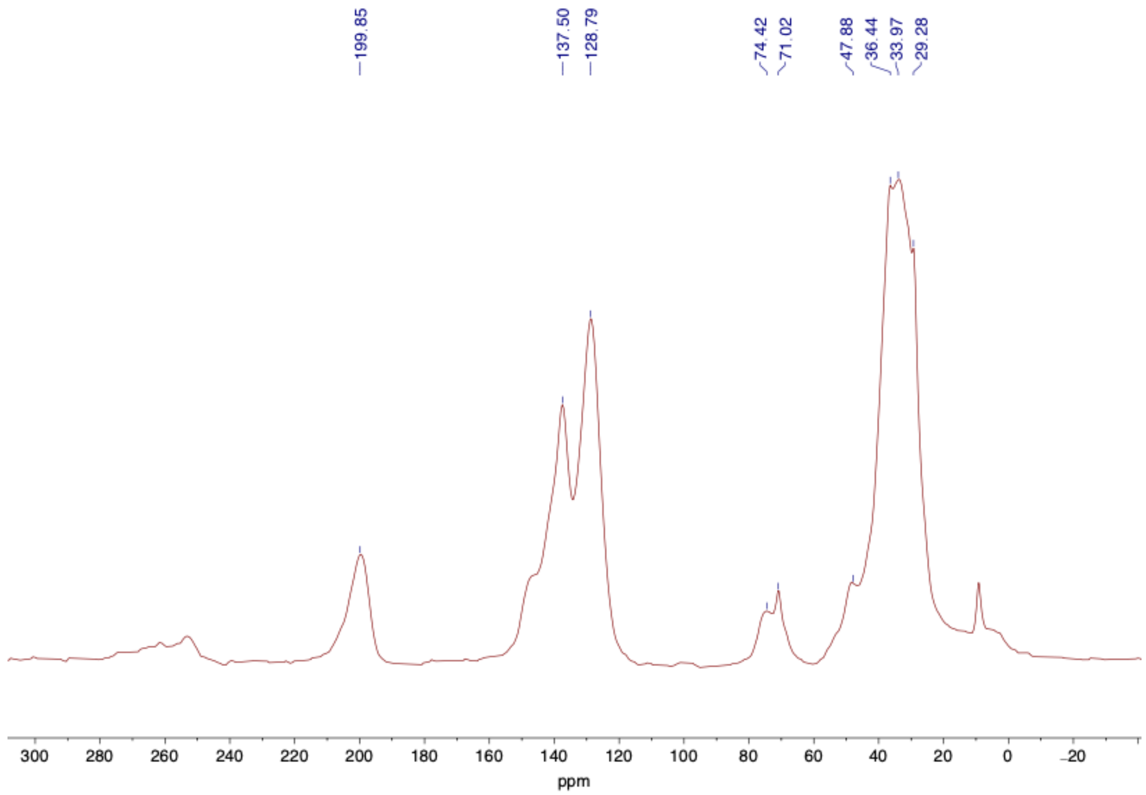

Figure S81. ^{13}C CP MAS NMR spectrum of **PAAK-6**.

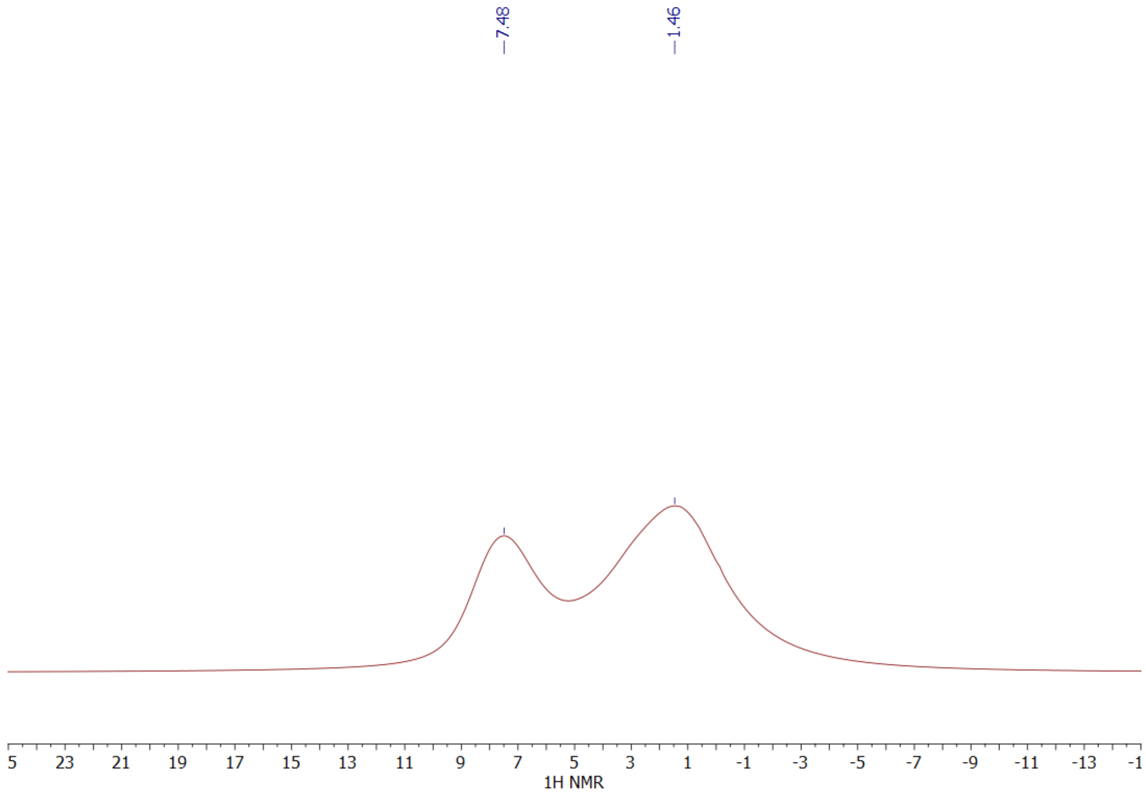

Figure S82. ^{1}H CP MAS NMR spectrum of **PAAK-6**.

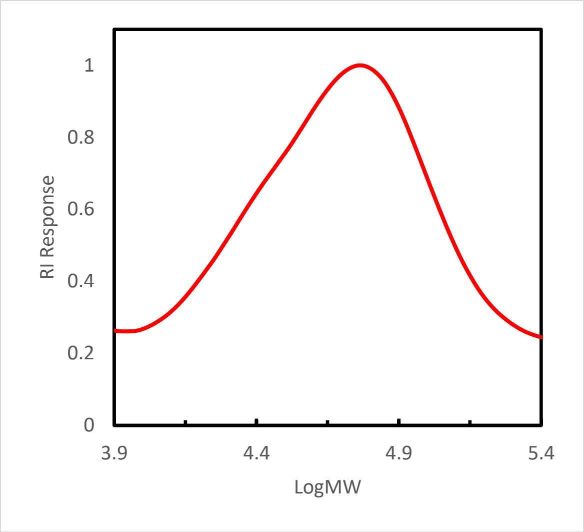

Figure S83. GPC chromatograph corresponding to **PAAK-6**.

PAAK-7

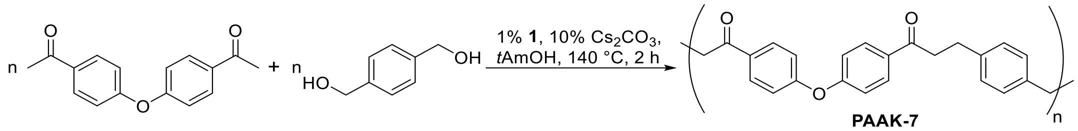

1,4-Dimethanolbenzene (69 mg, 0.5 mmol) and 4-acetylphenylether (127 mg, 0.5 mmol) were used. The polymer was obtained in 93% yield (165 mg) as a white solid.

^{13}C CP MAS NMR (100.6 MHz): δ 198.1, 160.1, 140.3, 130.8, 121.0, 75.3, 64.4, 42.7.

IR (ATR-FTIR, cm^{-1}): ν 3466w (O-H), 3055w, 2922w (C-H), 1672s (C=O), 1587s (C=C), 1497s, 1411w, 1233s (C-O), 1163s, 983m, 833s, 554m.

TGA: T_d = 362 °C

GPC: bimodal: MW(1) = 2.4 kDa, PDI = 1.4, MW(2) = 58.5 kDa, PDI = 1.8.

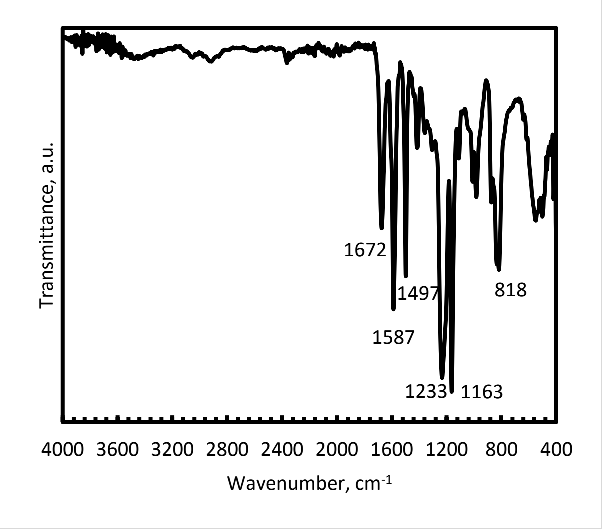

Figure S84. Infrared spectrum (ATR-FTIR) of polyketone **PAAK-7**.

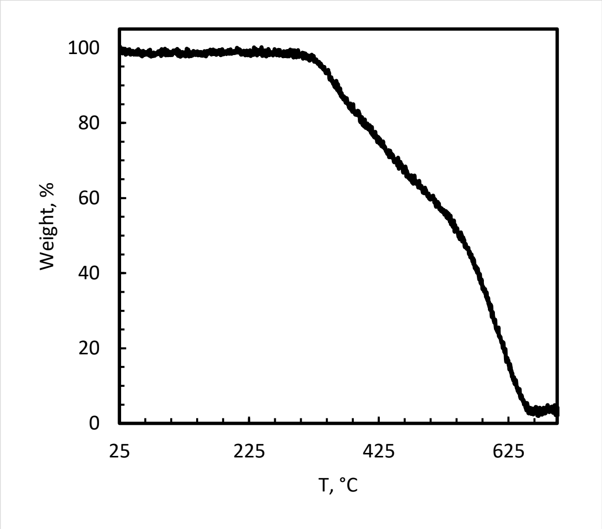

Figure S85. Mass loss as a function of temperature for polyketone **PAAK-7.**

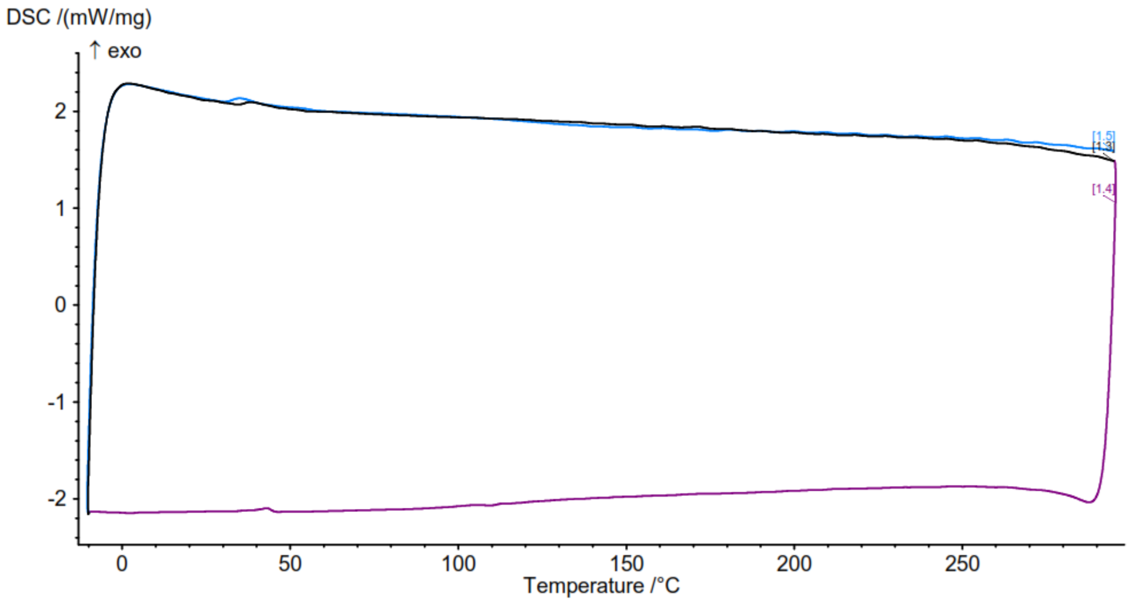

Figure S86. DSC trace corresponding to **PAAK-7**.

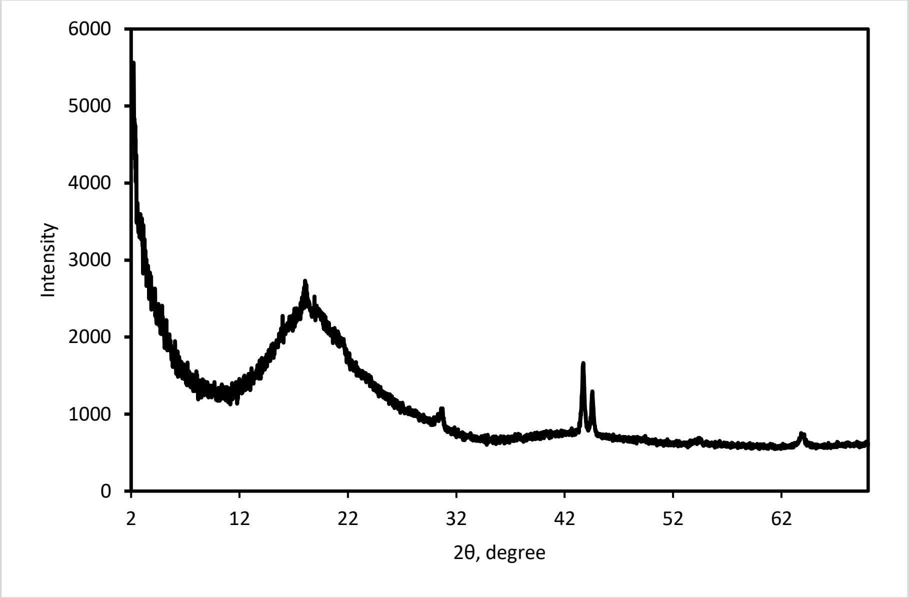

Figure S87. Experimental powder XRD patterns of **PAAK-7**. Unidentifiable crystalline phase presents. Peaks at high angles are from crystalline Teflon substrate.

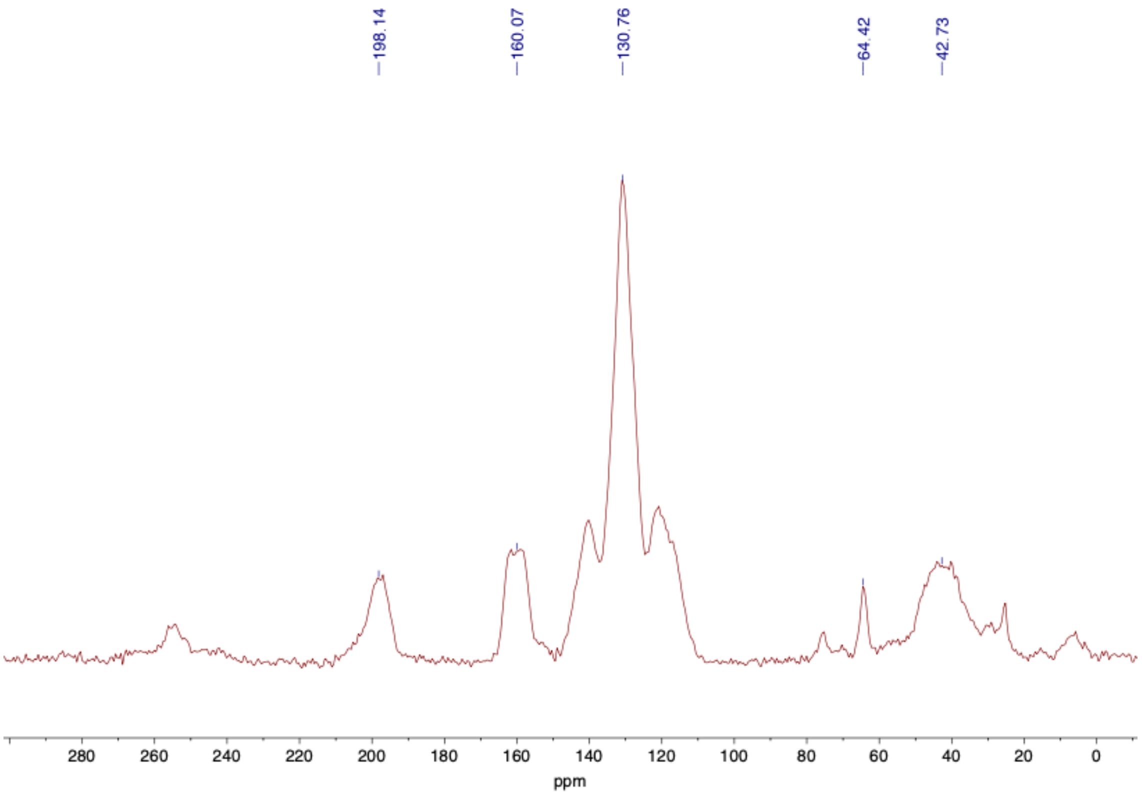

Figure S88. ^{13}C CP MAS NMR spectrum of **PAAK-7**.

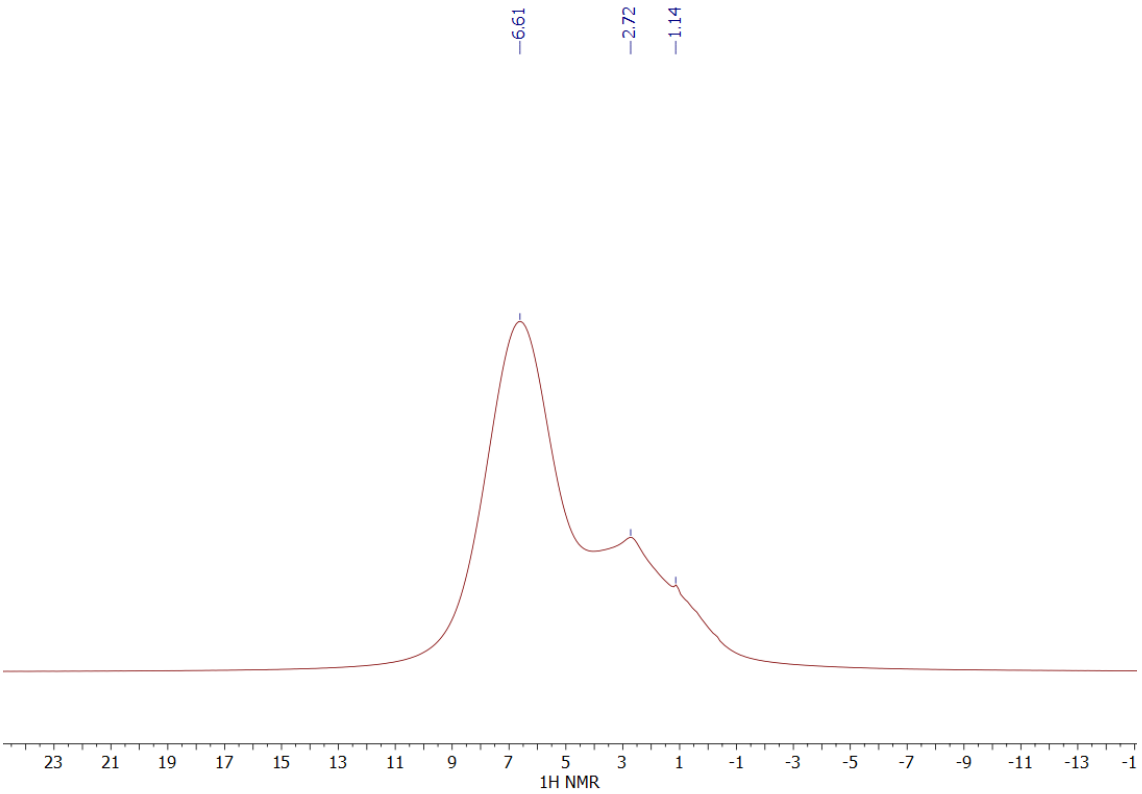

Figure S89. ^{1}H CP MAS NMR spectrum of **PAAK-7**.

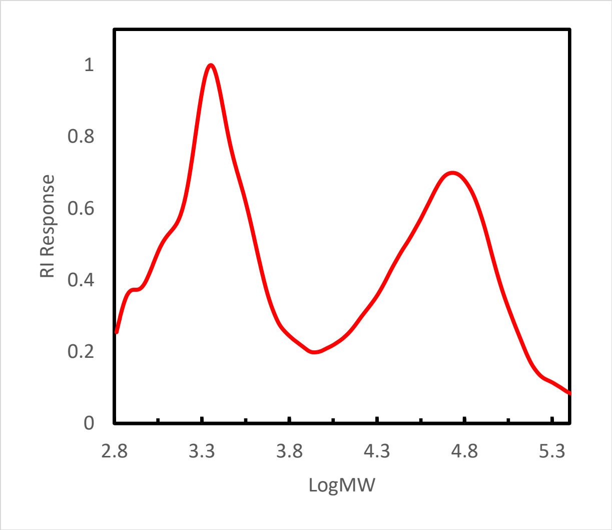

Figure S90. GPC chromatograph corresponding to **PAAK-7**.

PAAK-8

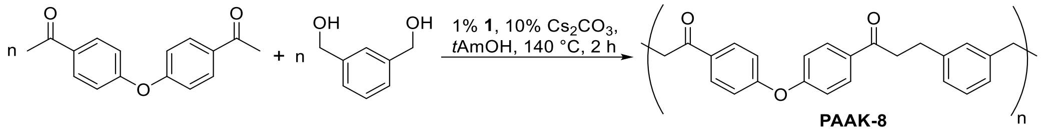

1,3-Dimethanolbenzene (69 mg, 0.5 mmol) and 4-acetylphenylether (127 mg, 0.5 mmol) were used. The polymer was obtained in 79% yield (140 mg) as a white solid.
^{13}C CP MAS NMR (100.6 MHz): δ 198.0, 162.1, 158.9, 142.2, 131.0, 121.6, 75.3, 64.5, 41.7.
IR (ATR-FTIR, cm^{-1}): ν 3066w (C-H), 2904w (C-H) 1674m (C=O), 1587s (C=C), 1499s, 1410w, 1234s, 1163s, 984w, 835m, 704w, 501w.
TGA: T_d = 365 °C
GPC: bimodal: MW(1) = 1.6 kDa, PDI = 1.3, MW(2) = 53.9 kDa, PDI = 1.4.

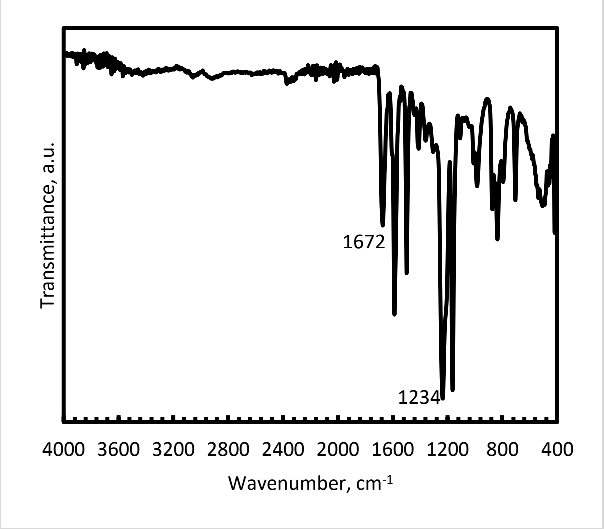

Figure S91. Infrared spectrum (ATR-FTIR) of polyketone **PAAK-8**.

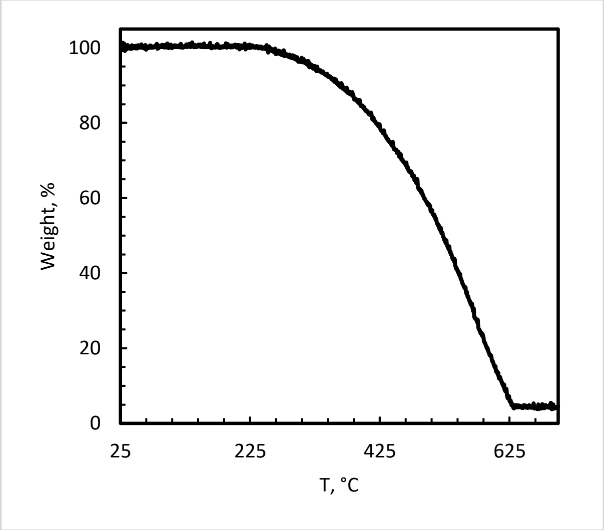

Figure S92. Mass loss as a function of temperature for polyketone **PAAK-8**.

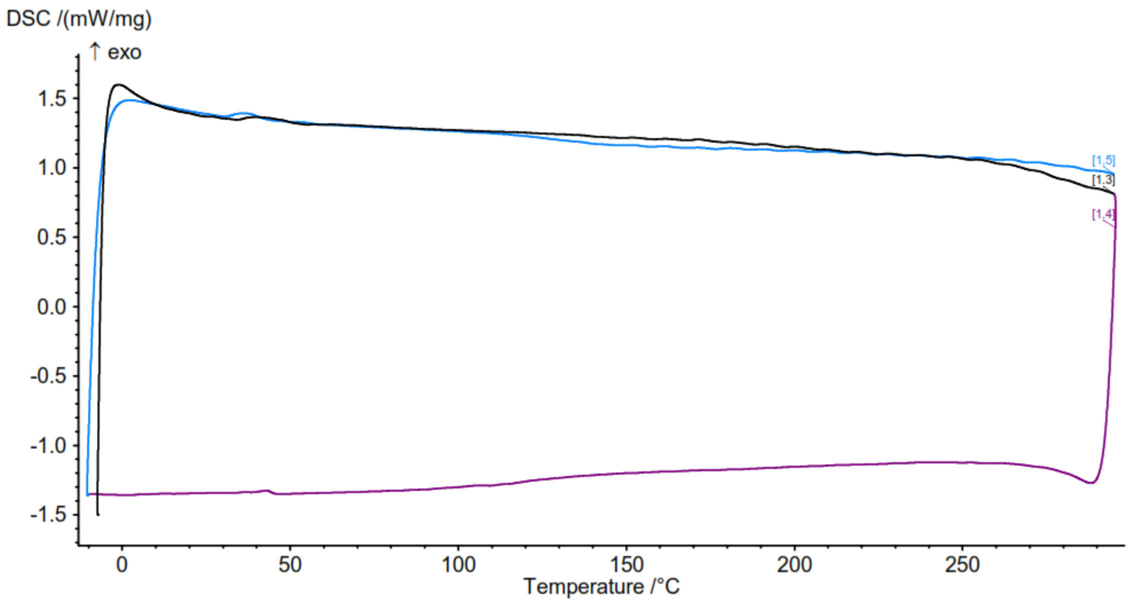

Figure S93. DSC trace corresponding to **PAAK-8**.

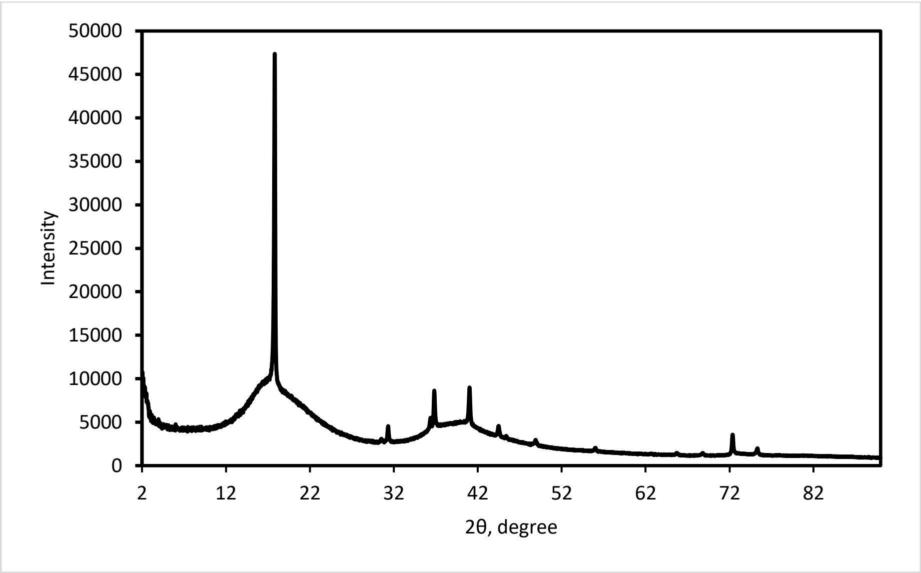

Figure S94. Experimental powder XRD patterns of **PAAK-8**. Unidentifiable crystalline phase present.

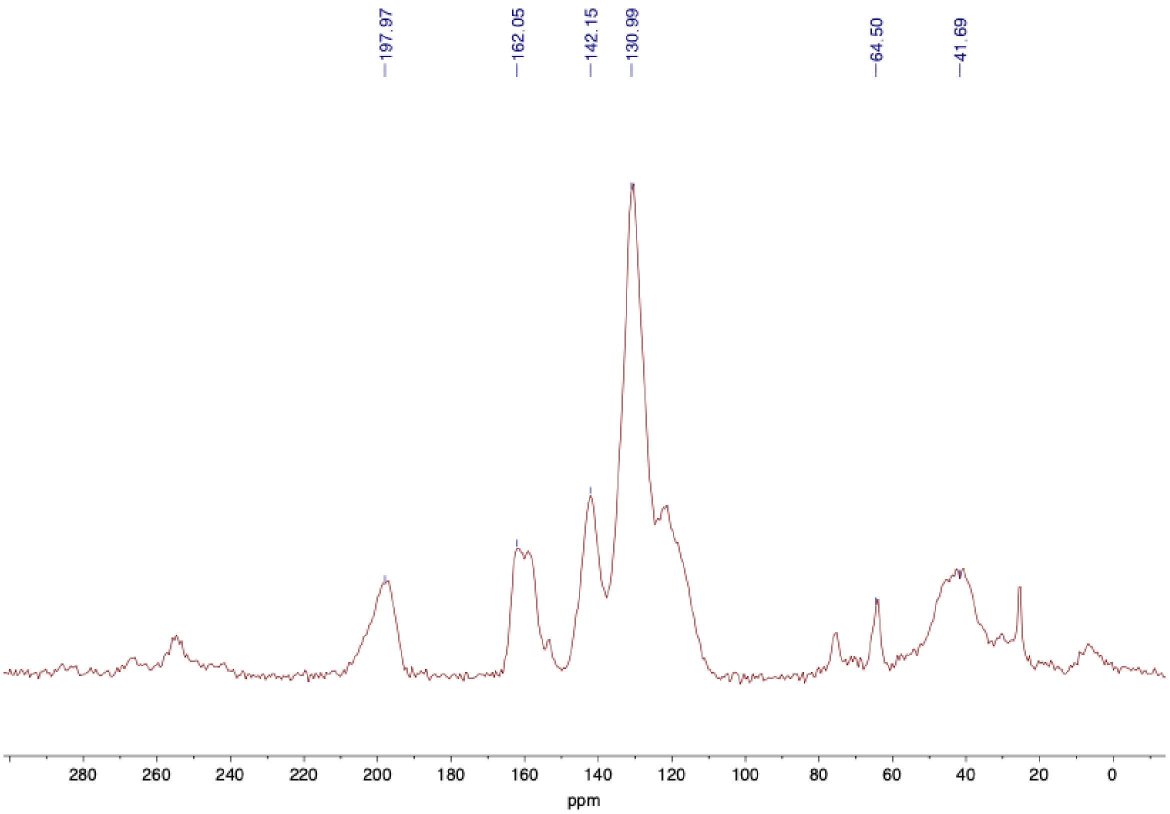

Figure S95. ^{13}C CP MAS NMR spectrum of **PAAK-8**.

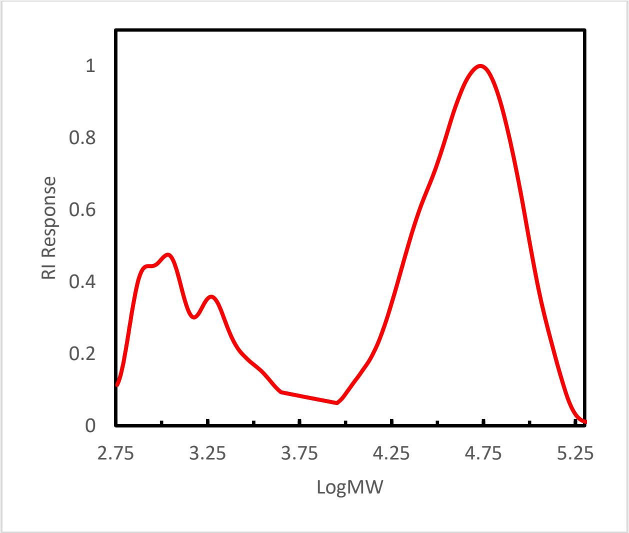

Figure S96. GPC chromatograph corresponding to **PAAK-8**.

PAAK-9

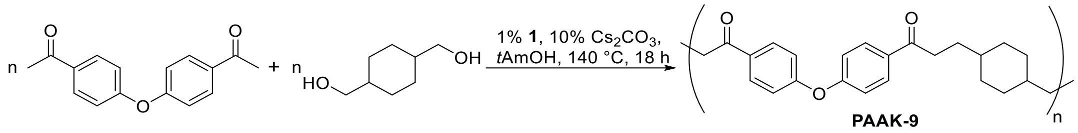

1,4-cyclohexanedimethanol (72 mg, 0.5 mmol) and 4-acetylphenylether (127 mg, 0.5 mmol) were used. The polymer was obtained in 71% yield (128 mg) as a white solid.

^{13}C CP MAS NMR (100.6 MHz): δ 198.6, 161.7, 159.1, 153.9, 131.6, 120.5, 76.4, 68.4, 48.4, 36.5, 32.3, 30.8.

IR (ATR-FTIR, cm^{-1}): ν 2916w (C-H), 2851w (C-H), 1674s (C=O), 1589s (C=C), 1499s, 1412w, 1236s (C-O), 1163s, 1011w, 835m, 567w.

TGA: T_d = 383 °C

GPC: monomodal: MW = 53.4 kDa, PDI = 1.7.

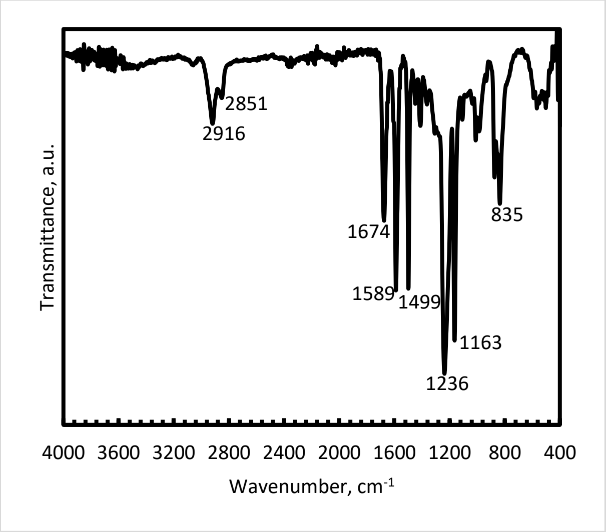

Figure S97. Infrared spectrum (ATR-FTIR) of polyketone **PAAK-9**.

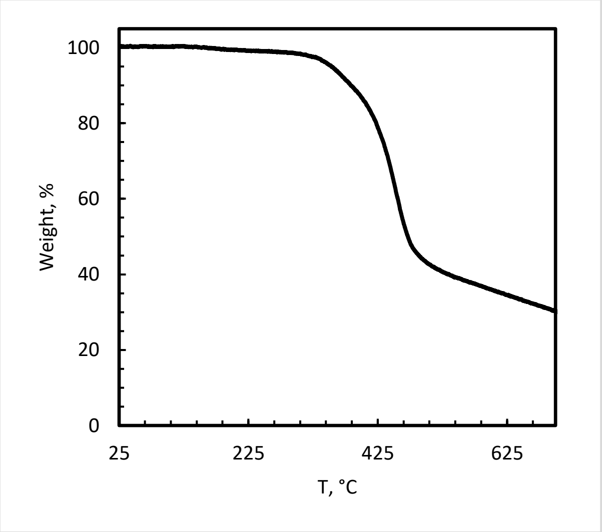

Figure S98. Mass loss as a function of temperature for polyketone **PAAK-9**.

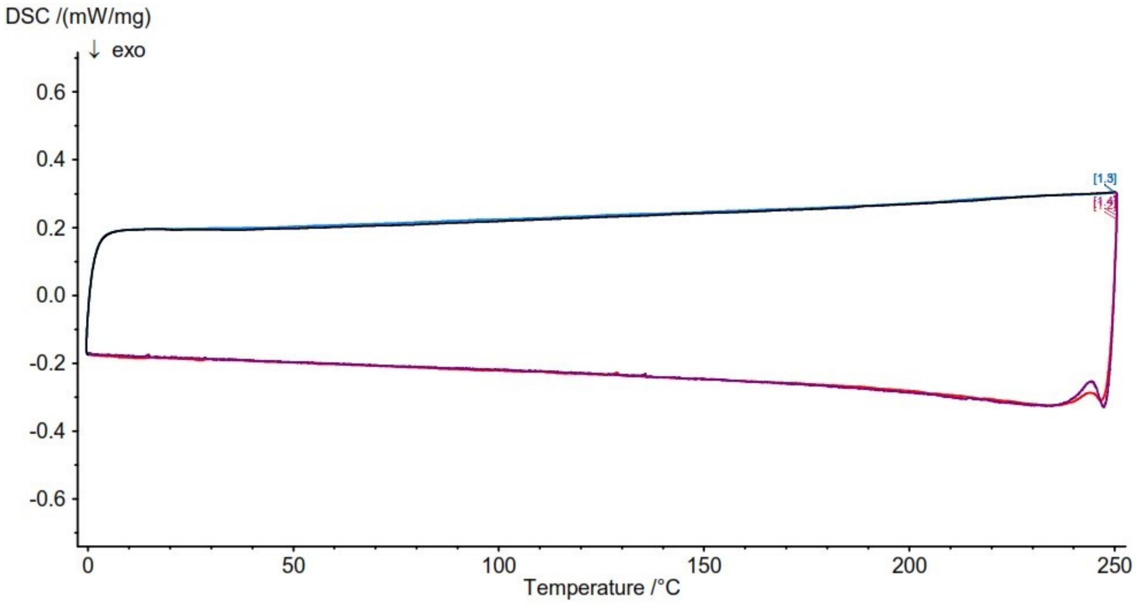

Figure S99. DSC trace corresponding to **PAAK-9** .

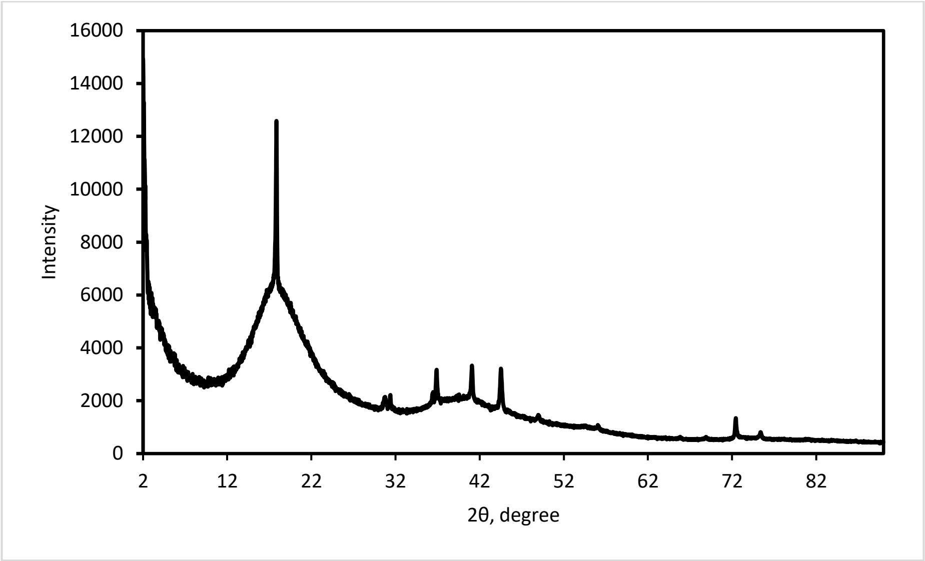

Figure S100. Experimental powder XRD patterns of **PAAK-9**. Unidentifiable crystalline phase present.

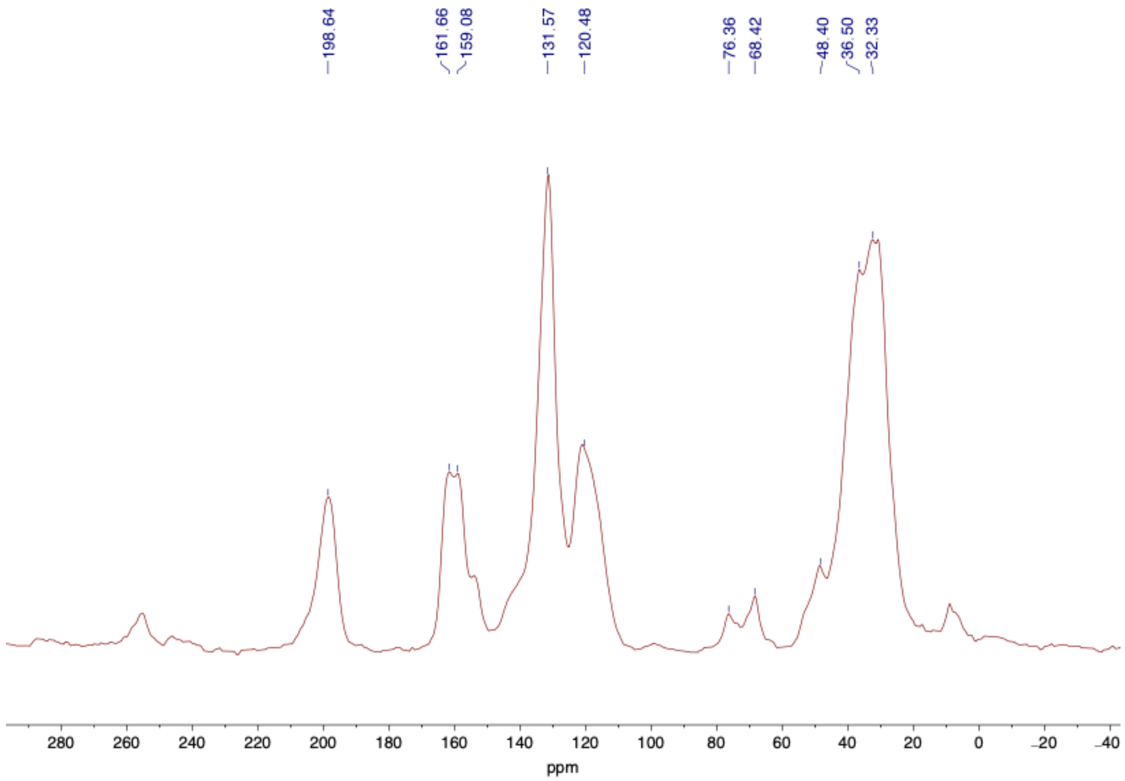

Figure S101. ^{13}C CP MAS NMR spectrum of **PAAK-9**.

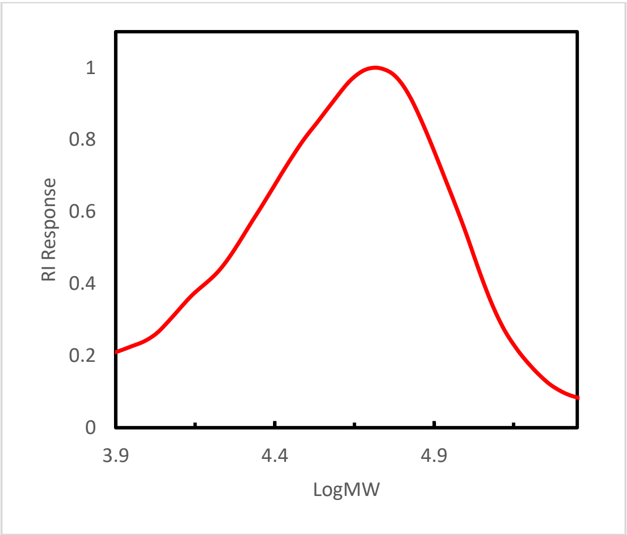

Figure S102. GPC chromatograph corresponding to **PAAK-9**.

PAAK-10

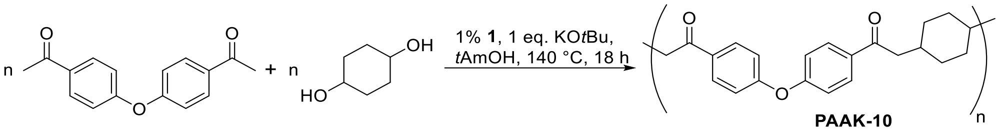

Cyclohexane-1,4-diol (58 mg, 0.5 mmol) and 4-acetylphenylether (127 mg, 0.5 mmol) were used. The polymer was obtained in 57% yield (95 mg) as a yellow solid.
^{13}C CP MAS NMR (100.6 MHz): δ 197.9, 157.6, 154.7, 129.8, 121.7, 71.5, 35.6, 28.7.
IR (ATR-FTIR, cm^{-1}): ν 3346w (O-H), 2918w (C-H), 1676w (C=O), 1591m (C=C), 1497s, 1231s (C-O), 1161s, 1013m, 826m, 509m.
TGA: T_d = 363 °C
GPC: broad dispersity, outside calibration curve: MW = 621.6 kDa, PDI = 108.6.

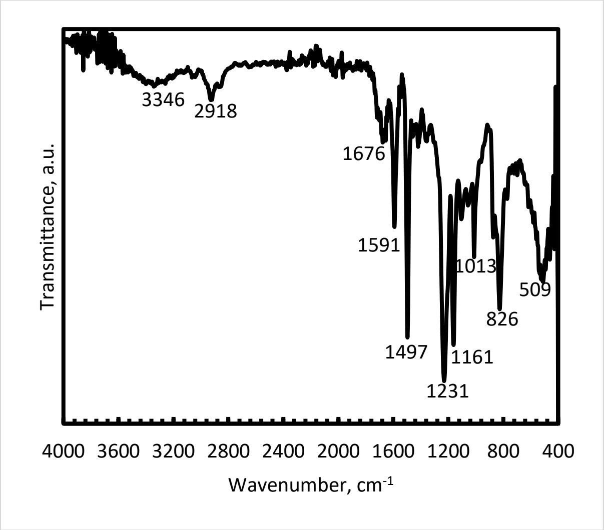

Figure S103. Infrared spectrum (ATR-FTIR) of polyketone **PAAK-10**.

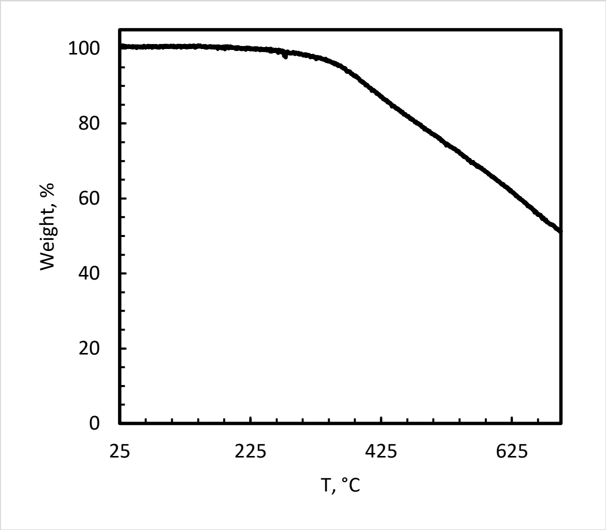

Figure S104. Mass loss as a function of temperature for polyketone **PAAK-10**.

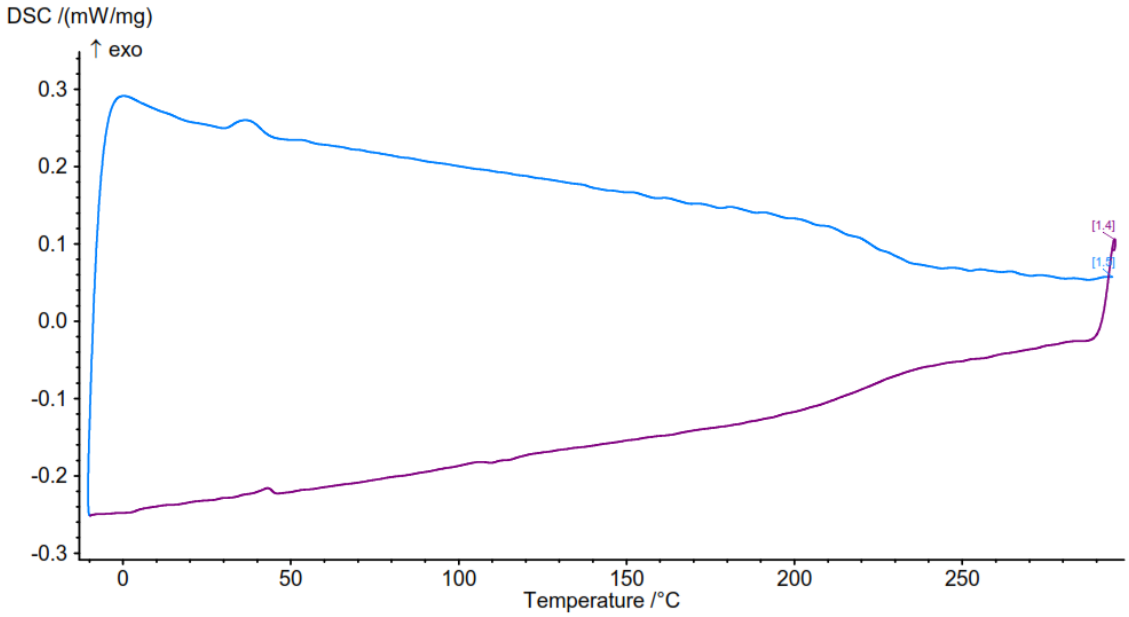

Figure S105. DSC trace corresponding to **PAAK-10**.

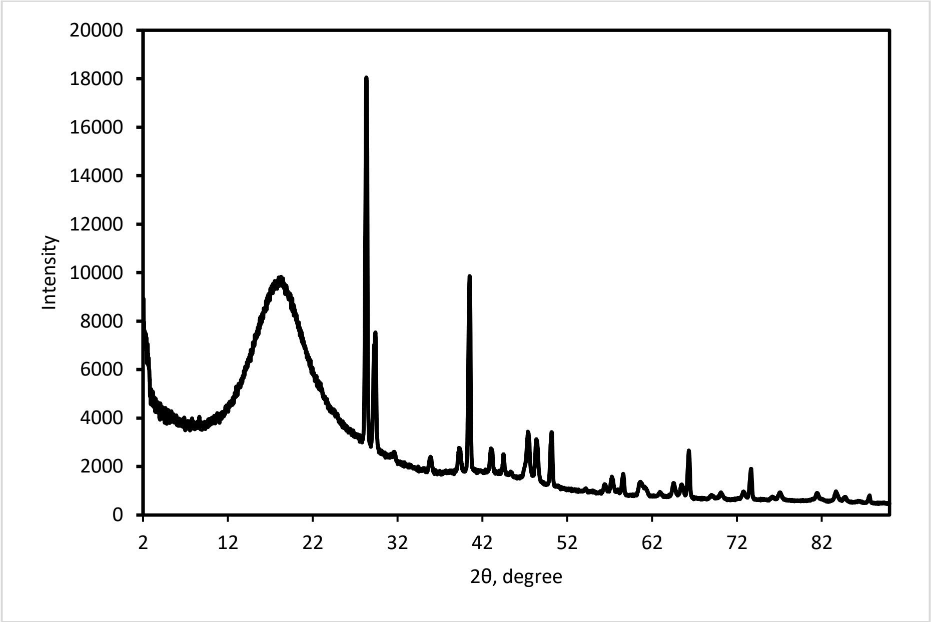

Figure S106. Experimental powder XRD patterns of **PAAK-10**. Crystalline peaks were indexed in the small-volume unit cell which remains unidentified.

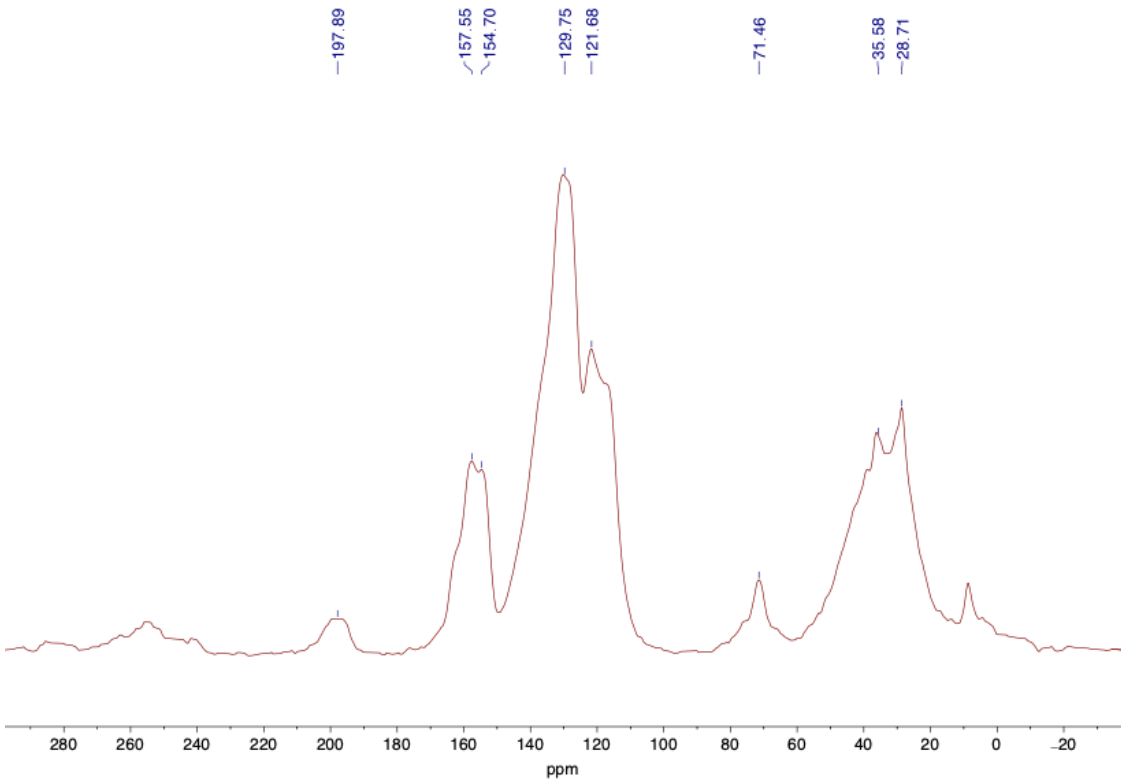

Figure S107. ^{13}C CP MAS NMR spectrum of **PAAK-10**.

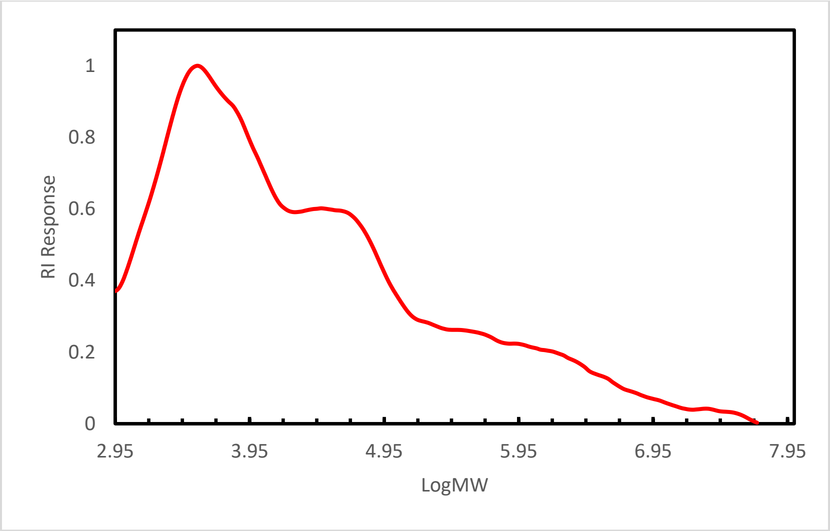

Figure S108. GPC chromatograph corresponding to **PAAK-10**.

PAAK-11

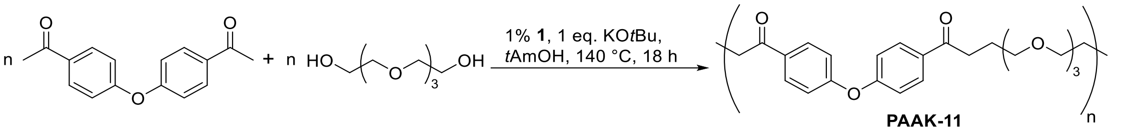

Tetraethylene glycol (115 mg, 0.6 mmol) and 4-acetylphenylether (127 mg, 0.5 mmol) were used. The polymer was obtained in 64 % yield (131 mg) as an orange solid.
^{13}C CP MAS NMR (100.6 MHz): δ196.8, 162.8, 157.9, 130.8, 116.0, 70.3, 67.8, 39.4.
IR (ATR-FTIR, cm^{-1}): ν 2922w (C-H), 2868w (C-H), 1655w (C=O), 1595s, 1501s, 1233s (C-O), 1096s, 827s, 507m.
TGA: T_d = 375 °C
GPC: broad dispersity, outside calibration curve: MW = 899.1 kDa, PDI = 148.8.

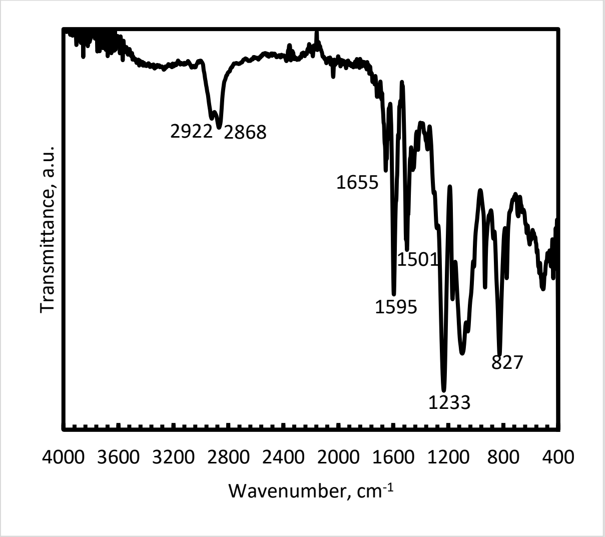

Figure S109. Infrared spectrum (ATR-FTIR) of polyketone **PAAK-11**.

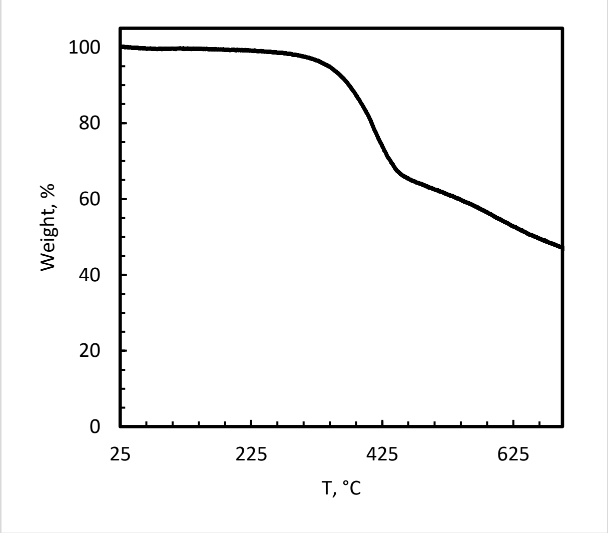

Figure S110. Mass loss as a function of temperature for polyketone **PAAK-11**.

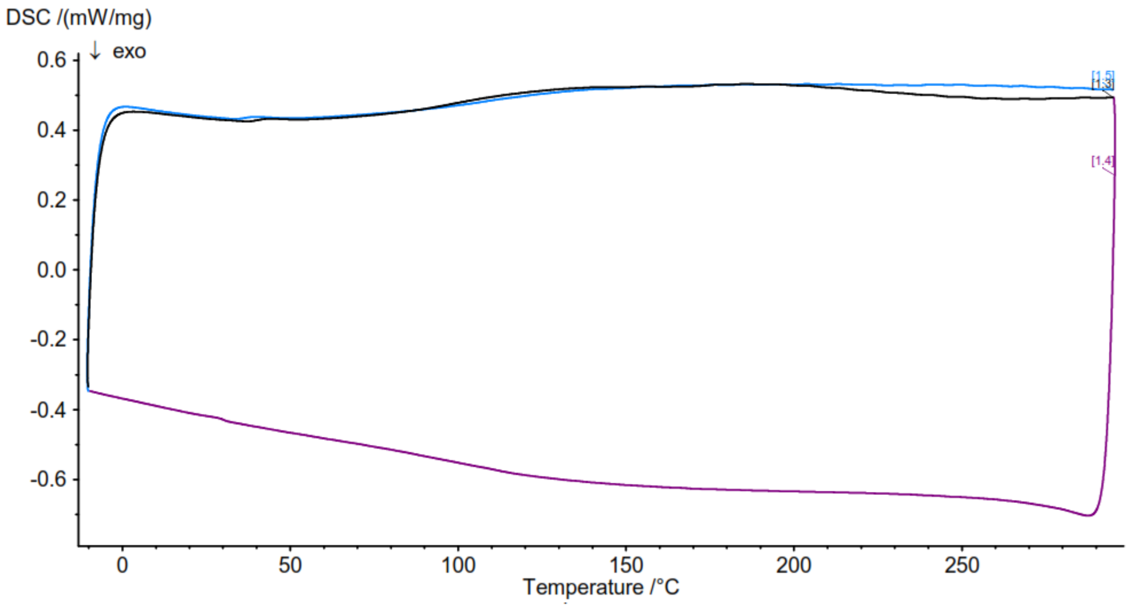

Figure S111. DSC trace corresponding to **PAAK-11**.

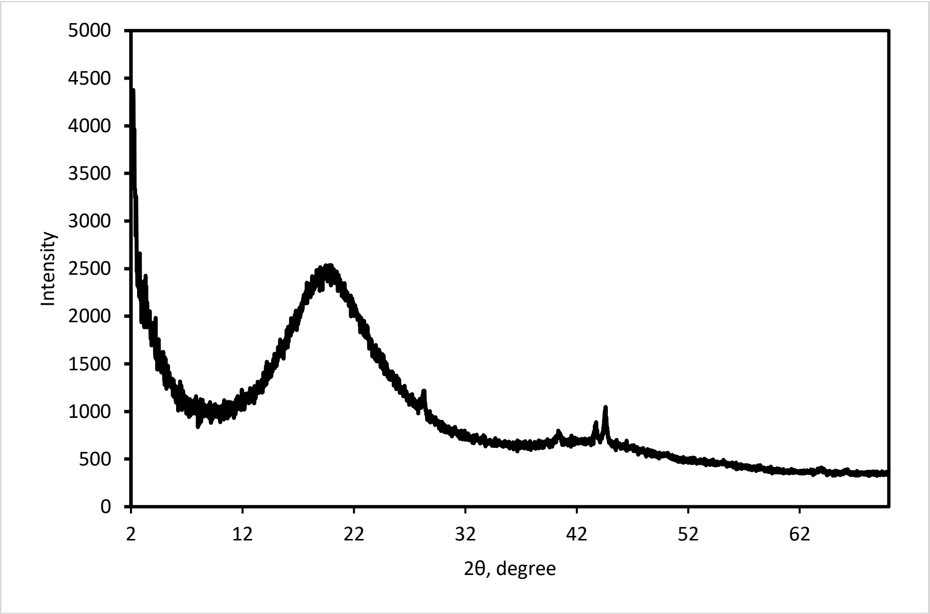

Figure S112. Experimental powder XRD patterns of **PAAK-11**. Some small crystalline peaks are from Teflon substrate, other were indexed in the small-volume unit cell which remains unidentified.

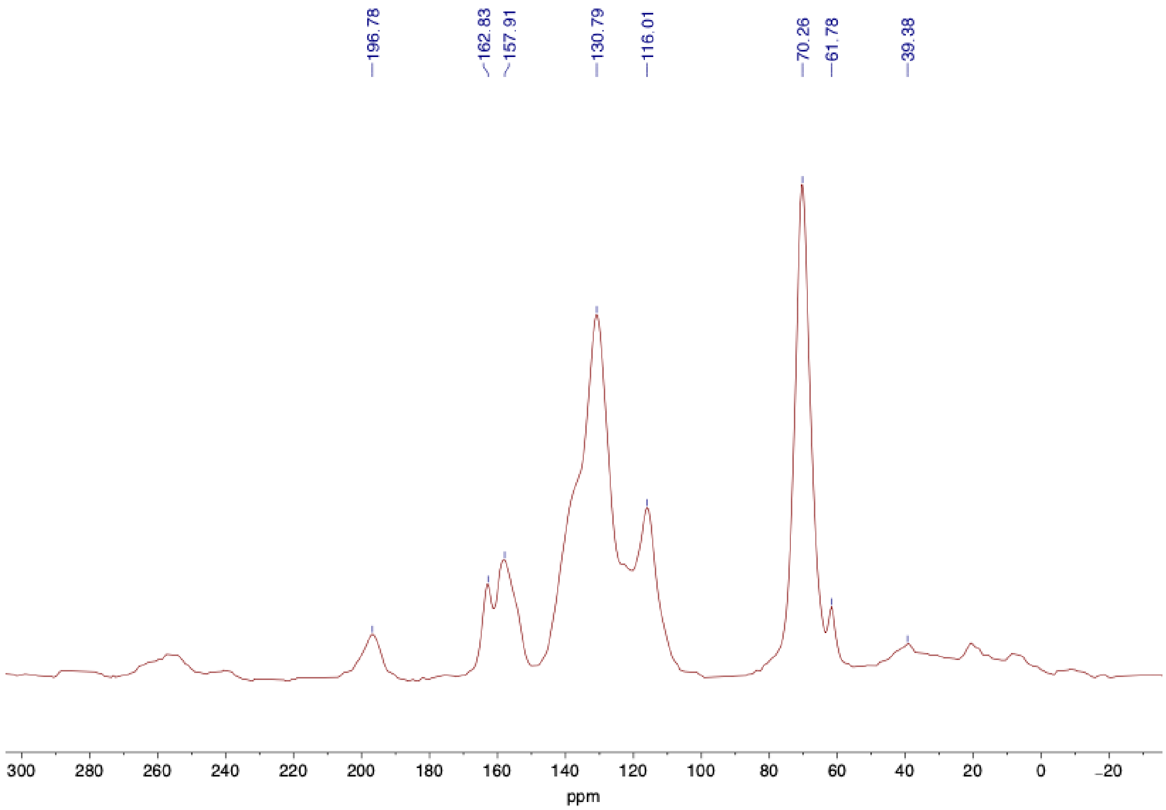

Figure S113. ^{13}C CP MAS NMR spectrum of **PAAK-11** coupling product.

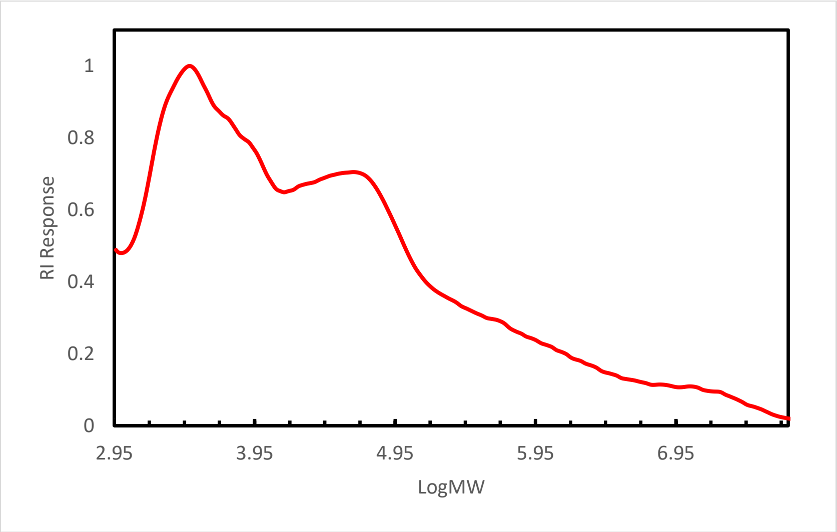

Figure S114. GPC chromatograph corresponding to **PAAK-11**.

PAAK-12

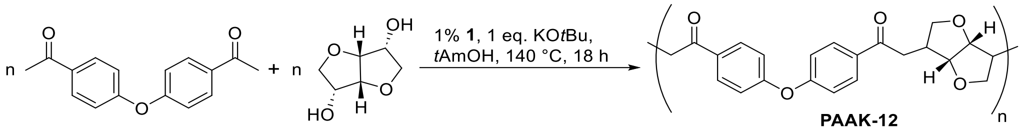

D-isosorbide (73 mg, 0.5 mmol) and 4-acetylphenylether (127 mg, 0.5 mmol) were used. The polymer was obtained in 52% yield (94 mg) as an orange solid.

^{13}C CP MAS NMR (100.6 MHz): δ 196.4, 158.1, 130.3, 120.5, 75.8, 39.9, 27.0.

IR (ATR-FTIR, cm^{-1}): ν 2963w (C-H), 1676w (C=O), 1591m (C=C), 1497s, 1231s (C-O), 1161s, 1013m, 827m, 527m.

TGA: T_d = 381 °C

GPC: bimodal: MW(1) = 3.0 kDa, PDI = 1.3, MW(2) = 53.6 kDa, PDI = 1.7.

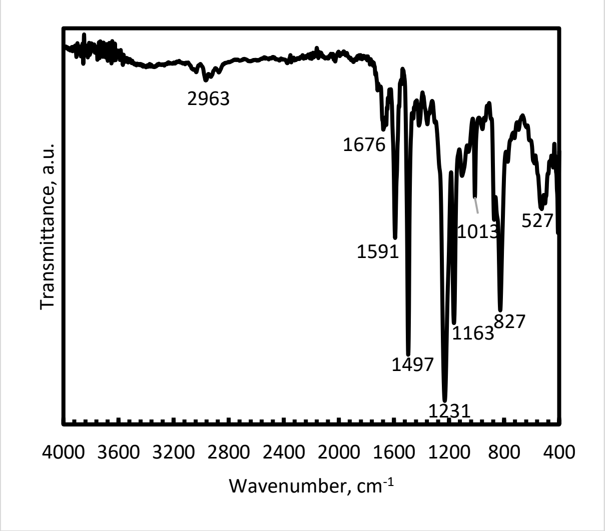

Figure S115. Infrared spectrum (ATR-FTIR) of polyketone **PAAK-12**.

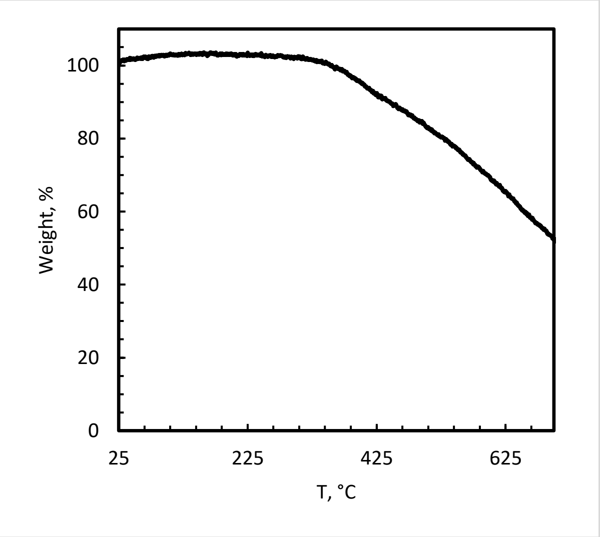

Figure S116. Mass loss as a function of temperature for polyketone **PAAK-12**.

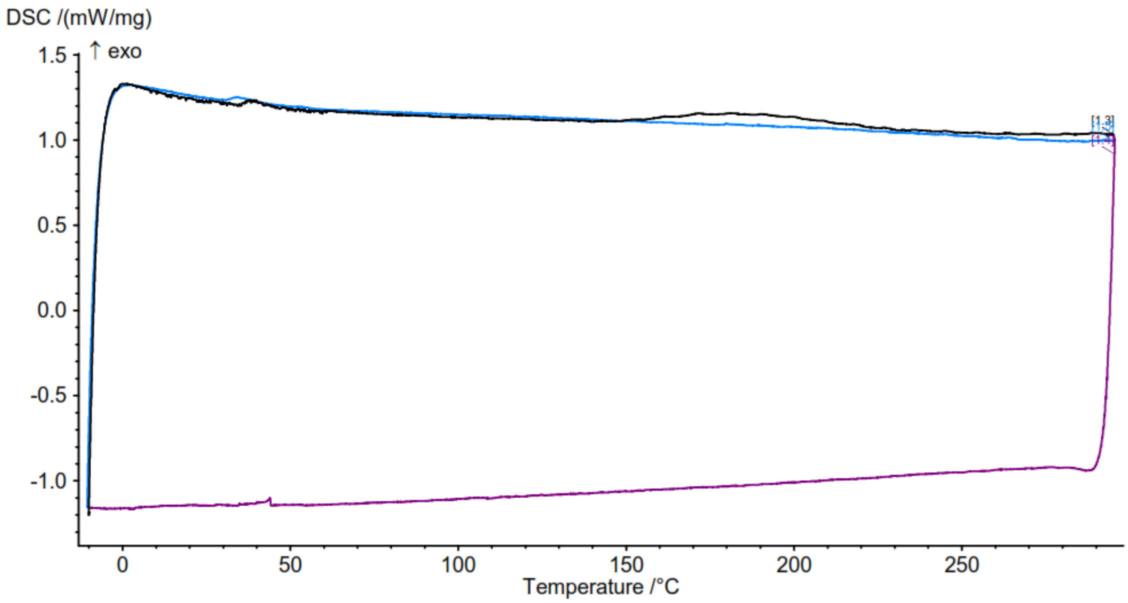

Figure S117. DSC trace corresponding to **PAAK-12**.

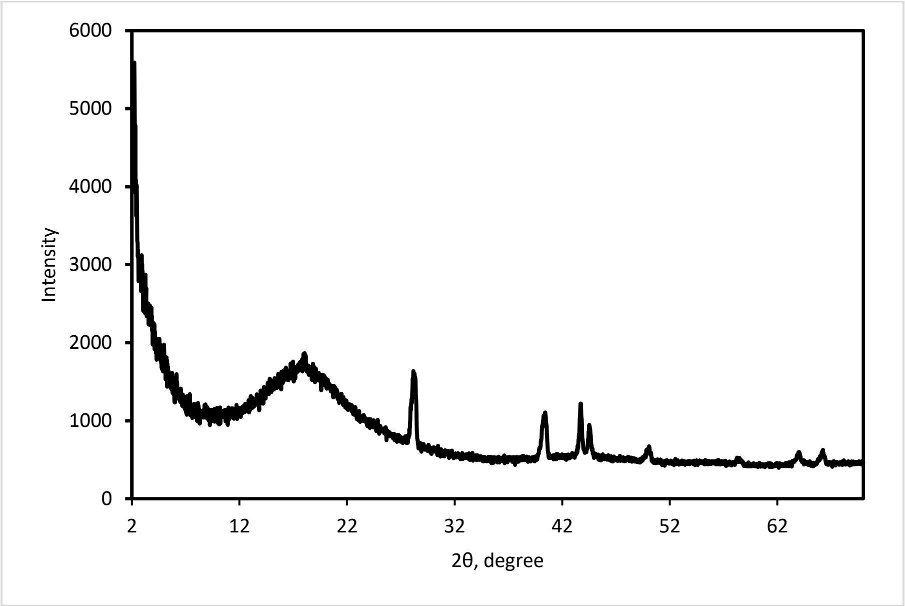

Figure S118. Experimental powder XRD patterns of **PAAK-12**. Crystalline peaks are either from Teflon substrate or were indexed in the small-volume unit cell which remains unidentified.

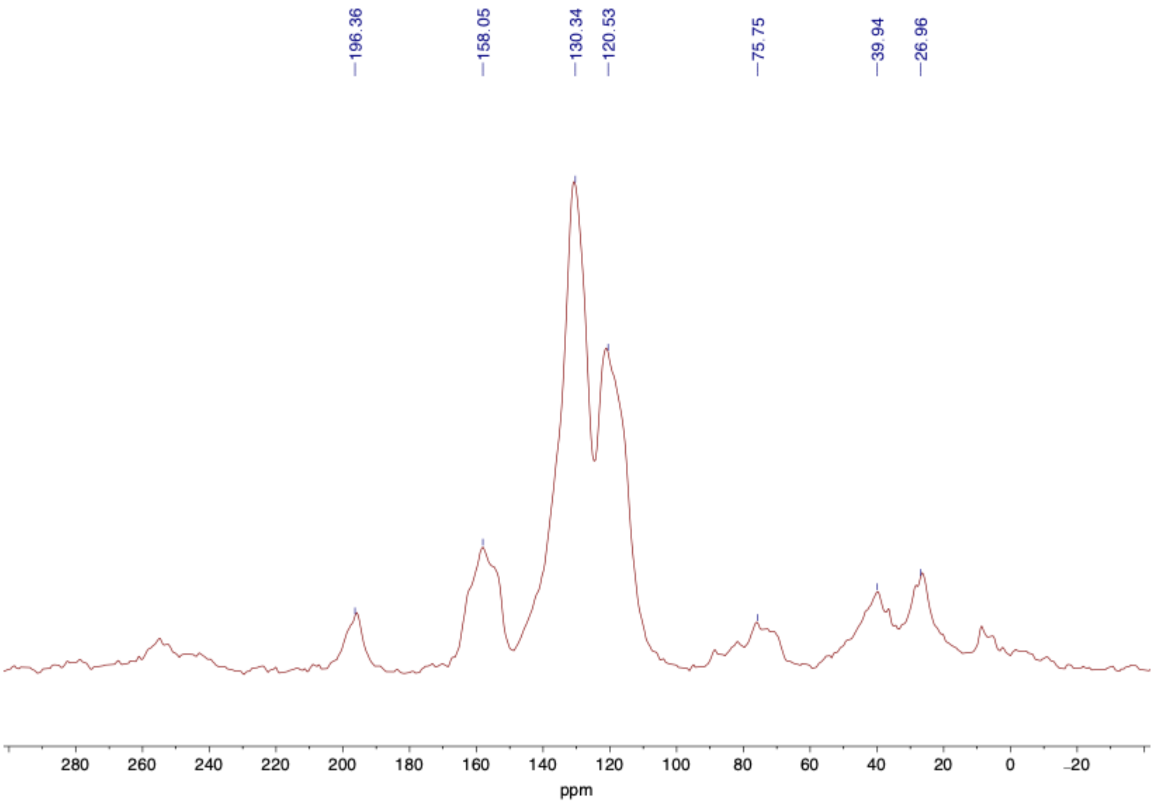

Figure S119. ^{13}C CP MAS NMR spectrum of **PAAK-12**.

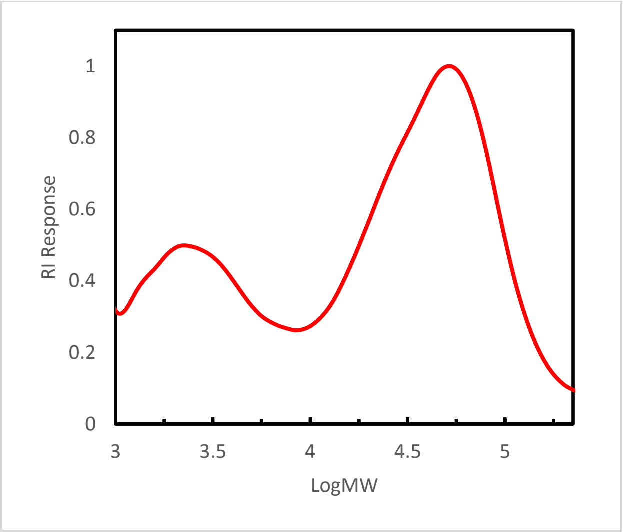

Figure S120. GPC chromatograph corresponding to **PAAK-12**.

1.6 Syntheses of polychalcones from diketones and dialdehydes

A 100 mL ampoule equipped with a J-Young's valve was charged with Cs_2CO_3 (16.5 mg, 0.05 mmol, 10 mol%), dialdehyde (0.5 mmol) and diketone (0.5 mmol). The flask was sealed under an argon atmosphere and *tert*-amyl alcohol (5 mL) was added before heating to 140 °C for 18 h with stirring. After this period, the reaction vessel was allowed to cool to room temperature. To the resulting mixture, 5 mL of water was added and the flask was heated at 90 °C for 1 h. The precipitate was filtered and dried under reduced pressure at 120 °C.

PCH-1

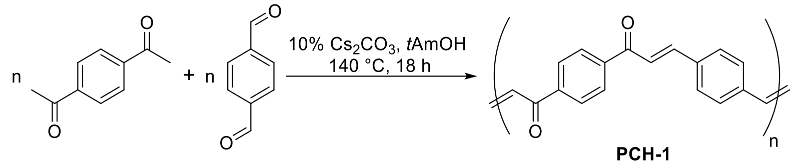

Terephthaldehyde (68 mg, 0.5 mmol) and 1,4-diacetylbenzene (81 mg, 0.5 mmol) were used. The polymer was obtained with 93% yield (121 mg) as a yellow solid.

IR (ATR-FTIR, cm^{-1}): ν 3472w (C-H), 3036w, 2834w, 1657m (C=O), 1599s (C=C), 1566m, 1501w, 1402w, 1327m, 1211s, 980m, 810s.

TGA: Td = 365 °C

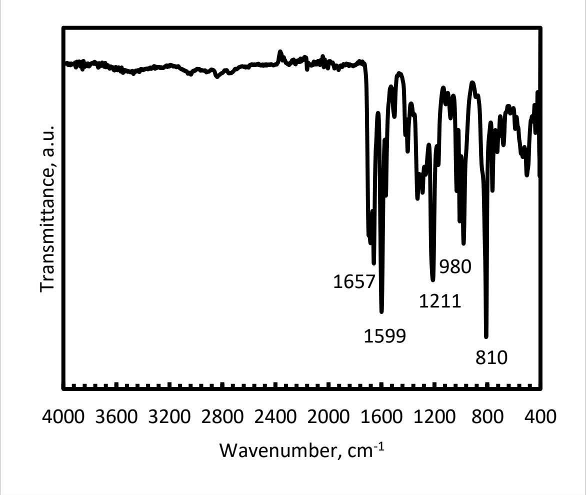

Figure S121. Infrared spectrum (ATR-FTIR) for polychalcone **PCH-1**.

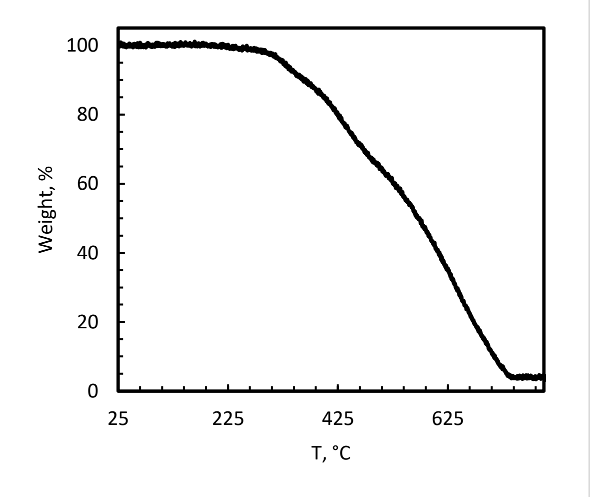

Figure S122. Mass loss as a function of temperature for polychalcone **PCH-1**.

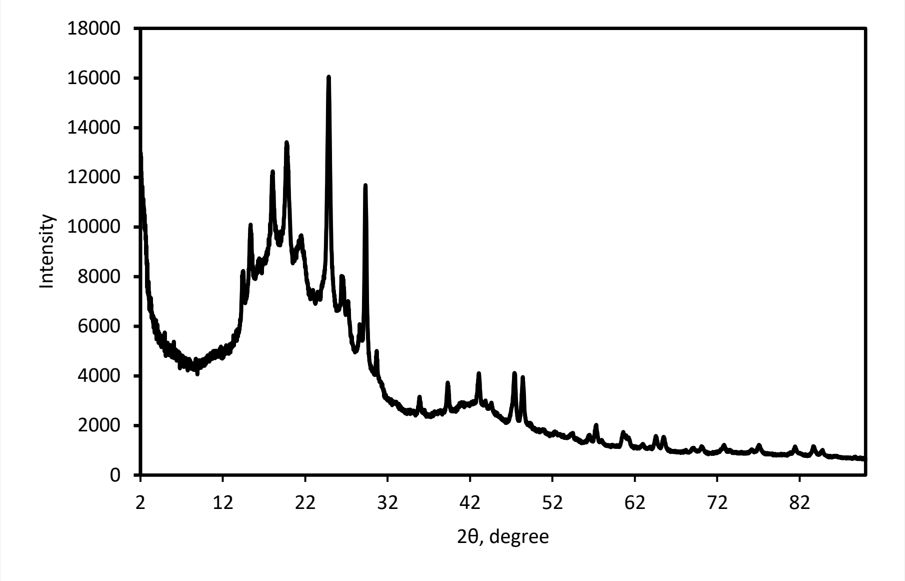

Figure S123. Experimental powder XRD patterns of polychalcone **PCH-1**.

PCH-7

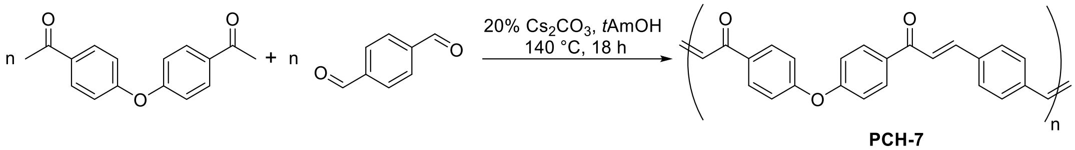

Terephthaldehyde (68 mg, 0.5 mmol) and 4-acetylphenyl ether (127 mg, 0.5 mmol) were used. The polymer was obtained in 91% yield (161 mg) as a yellow solid.

IR (ATR-FTIR, cm^{-1}): ν 3460w (C-H), 3044w, 1657m (C=O), 1589s (C=C), 1499m, 1418w, 1333m, 1215s, 1163s, 978w, 818s, 500m.

TGA: T_d = 393 °C
DSC: T_m = 248 °C

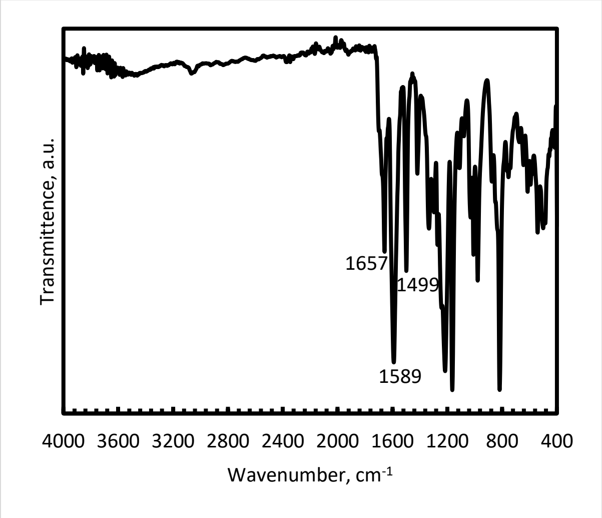

Figure S124. Infrared spectrum (ATR-FTIR) of polychalcone **PCH-7**.

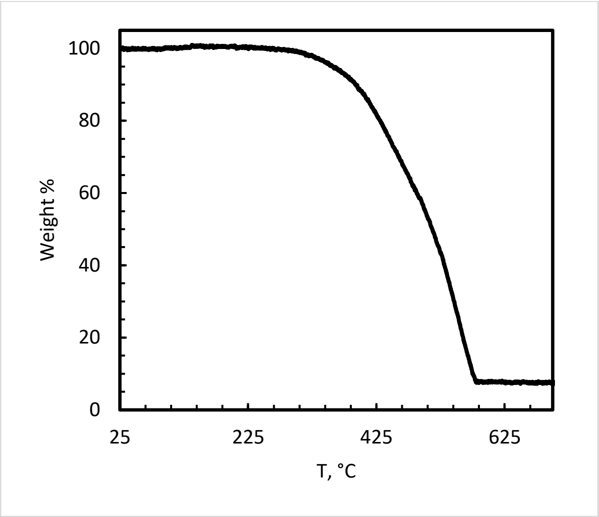

Figure S125. Mass loss as a function of temperature for polychalcone **PCH-7**.

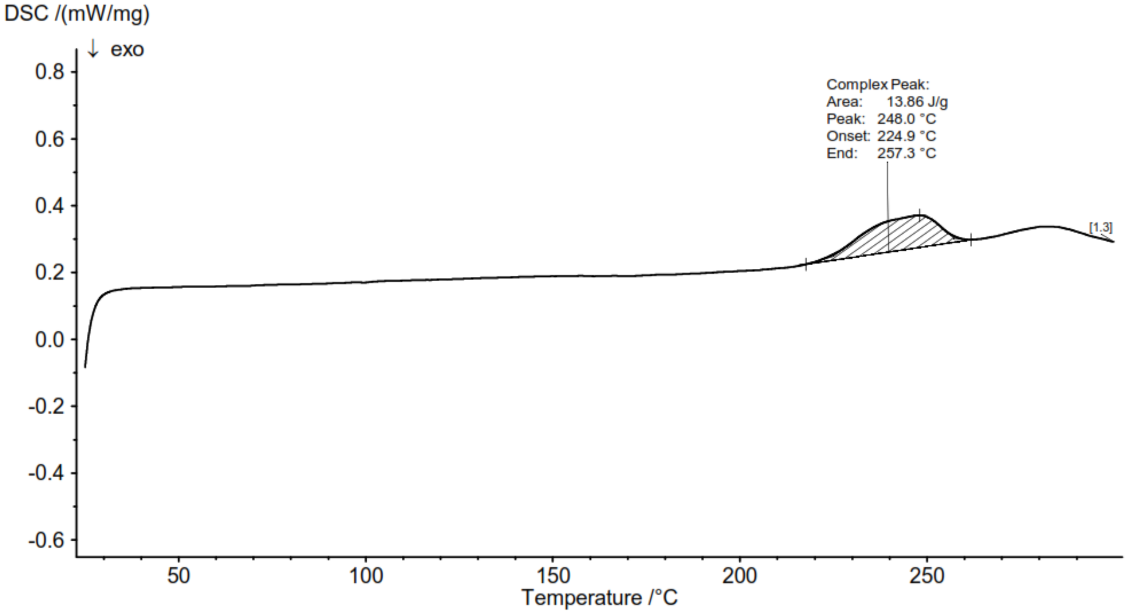

Figure S126. DSC trace corresponding to **PCH-7**.

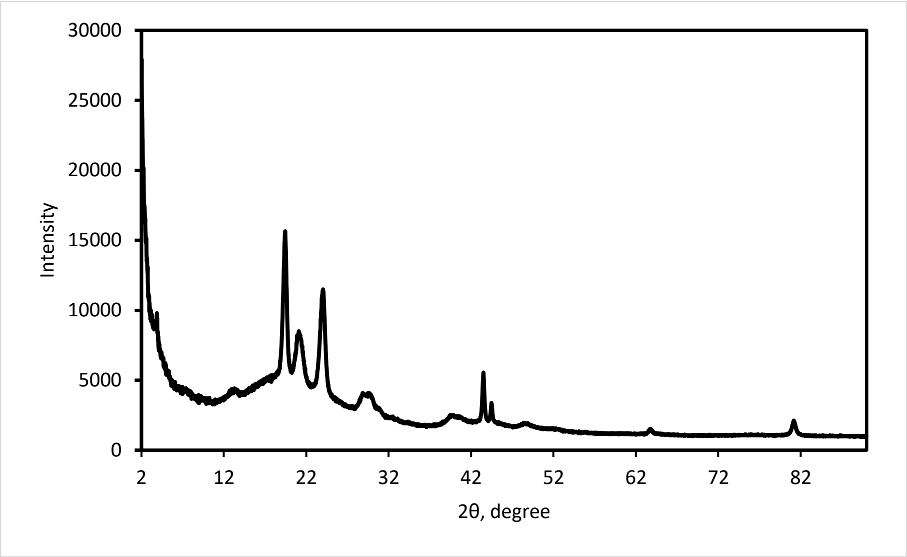

Figure S127. Experimental powder XRD patterns of polychalcone **PCH-7**.

1.7 Reaction 4-acetylphenyl ether and potassium *tert*-butoxide.

A 50 mL ampoule equipped with a J-Young's valve was charged with KO*t*Bu (56 mg, 0.5 mmol, 1 eq. mol%), 4-acylaryl ether (127 mg, 0.5 mmol). The flask was sealed under an argon atmosphere and *tert*-amyl alcohol (5 mL) was added before heating to 140 °C for 18 h with stirring. After this period, the reaction vessel was allowed to cool to room temperature. To the resulting mixture, 5 mL of 1 M solution of HCl was added and the flask was heated at 90 °C for 1 h. The precipitate was filtered, washed with water, acetone and dichloromethane and dried under reduced pressure at 120 °C giving the product (55 mg, 50% yield) as a white solid.
IR (ATR-FTIR, cm^{-1}): ν 1680w, 1591w, 1495s, 1233s, 1163m, 826m, 507w.
TGA: T_d = 455 °C
DSC: T_g = 230 °C

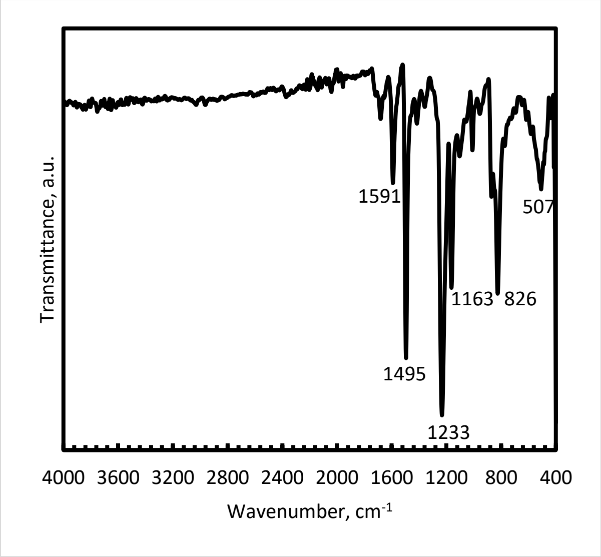

Figure S128. Infrared spectrum (ATR-FTIR) of the reaction product of 4-acetylphenyl ether and potassium tert-butoxide.

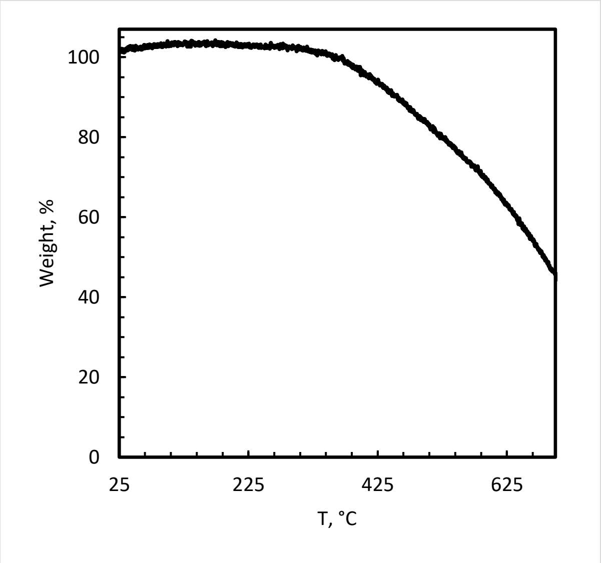

Figure S129. Mass loss as a function of temperature for the reaction product of 4-acetylphenyl ether and potassium tert-butoxide.

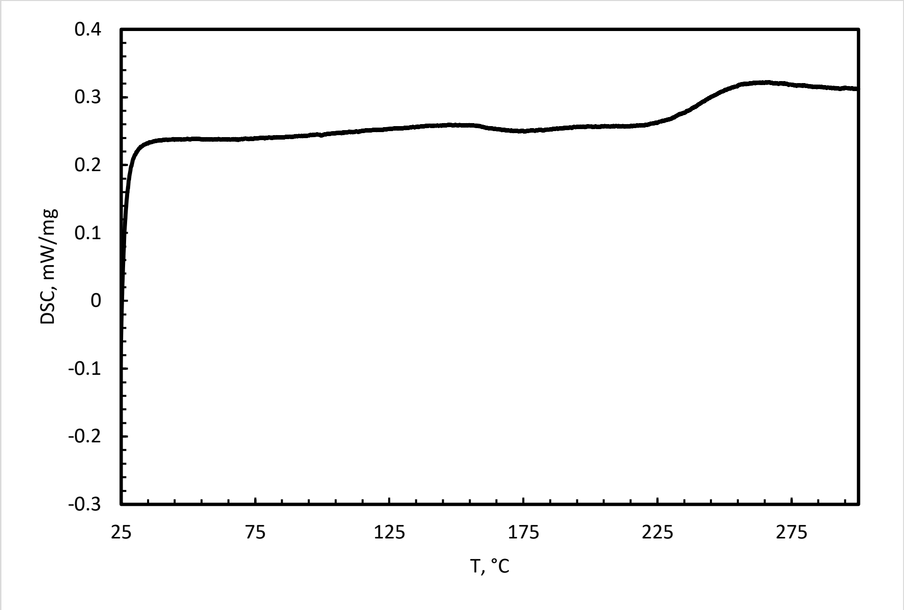

Figure S130. DSC trace corresponding to the reaction product of 4-acetylphenyl ether and potassium *tert*-butoxide.

1.8 Syntheses of polyketones in the presence of hydrogen atmosphere.

*Synthesis of **PAAK-1** in the presence of hydrogen atmosphere.*
A 100 mL ampoule equipped with a J-Young's valve was charged with pre-catalyst **1** (2.5 mg, 0.005 mmol, 1 mol%), 1,4-benzenedimethanol (69 mg, 0.5 mmol), 1,4-diacetylketone (81 mg, 0.5 mmol) and Cs_2CO_3 (16.5 mg, 0.05 mmol, 10 mol%). *tert*-Amyl alcohol (5 mL) was added and the flask was sealed under a hydrogen atmosphere before heating to 140 °C for 18 h with stirring. After this period, the reaction vessel was allowed to cool to room temperature. To the resulting mixture, 5 mL of 1 M HCl was added and the flask had been heated at 90 °C for 1 h. The yellow precipitate (126 mg, 95% yield) was filtered and dried under reduced pressure at 120 °C.
IR (ATR-FTIR, cm^{-1}): ν 3476w (O-H), 2920w (C-H), 1676s (C=O), 1605m (C=C), 1510w, 1402m, 1267m, 1211s, 982m, 818s, 550m.
TGA: Td = 397 °C

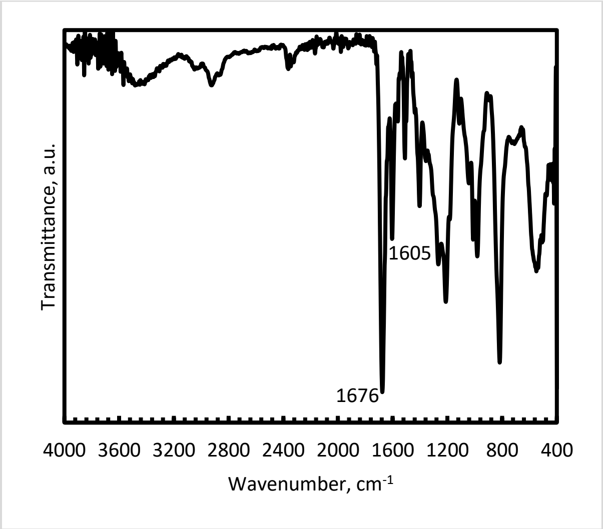

Figure S131. Infrared spectrum (ATR-FTIR) of polyketone **PAAK-1** obtained in the atmosphere of hydrogen.

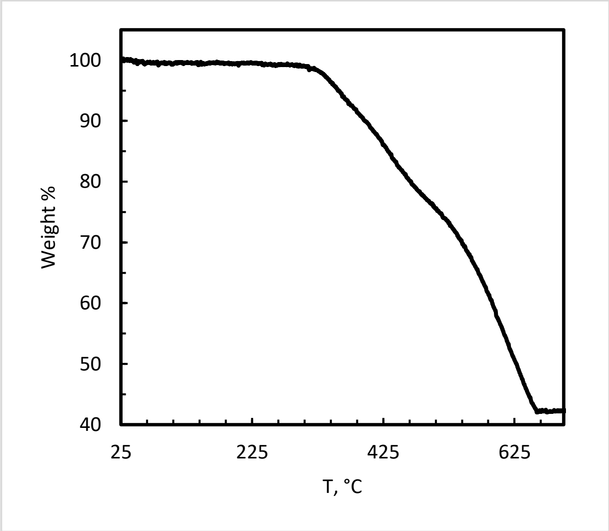

Figure S132. Mass loss as a function of temperature for polyketone **PAAK-1** obtained in the atmosphere of hydrogen.

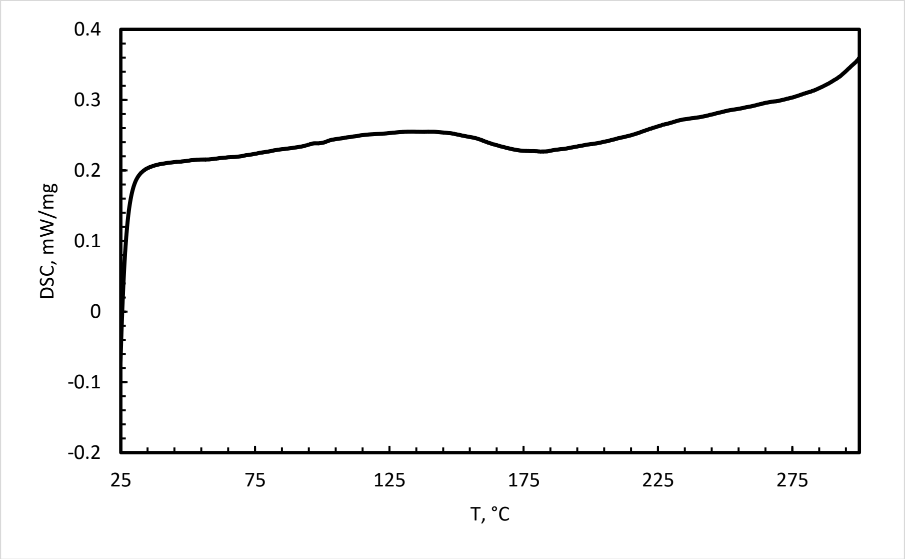

Figure S133. DSC trace corresponding to polyketone **PAAK-1** obtained in the atmosphere of hydrogen.

Synthesis of ***PAAK-7*** *in the presence of hydrogen atmosphere.*

A 100 mL ampoule equipped with a J-Young's valve was charged with pre-catalyst **1** (2.5 mg, 0.005 mmol, 1 mol%), 1,4-benzenedimethanol (69 mg, 0.5 mmol), 4-acetylphenyl ether (127 mg, 0.5 mmol) and Cs_2CO_3 (16.5 mg, 0.05 mmol, 10 mol%). *tert*-Amyl alcohol (5 mL) was added and the flask was sealed under a hydrogen atmosphere before heating to 140 °C for 18 h with stirring. After this period, the reaction vessel was allowed to cool to room temperature. To the resulting mixture, 5 mL of 1 M HCl was added and the flask had been heated at 90 °C for 1 h. The white precipitate (177 mg, 99% yield) was filtered and dried under reduced pressure at 120 °C.

IR (ATR-FTIR, cm^{-1}): ν 2914w (C-H), 1674m (C=O), 1585s (C=C), 1497m, 1233s, 1163s, 822m, 548m.

TGA: Td = 371 °C

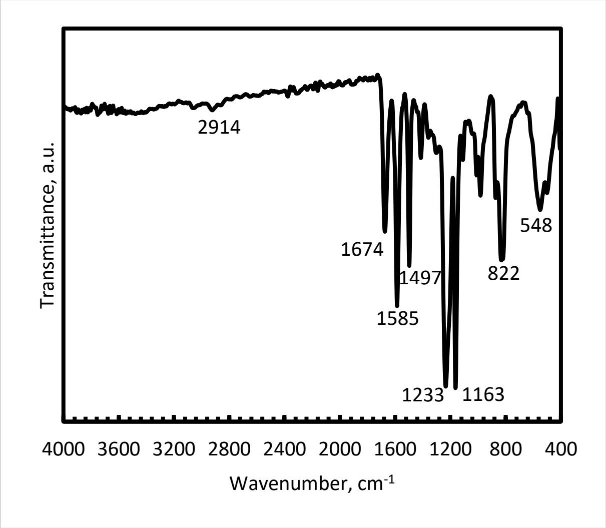

Figure S134. Infrared spectrum (ATR-FTIR) of polyketone **PAAK-7** obtained in the atmosphere of hydrogen.

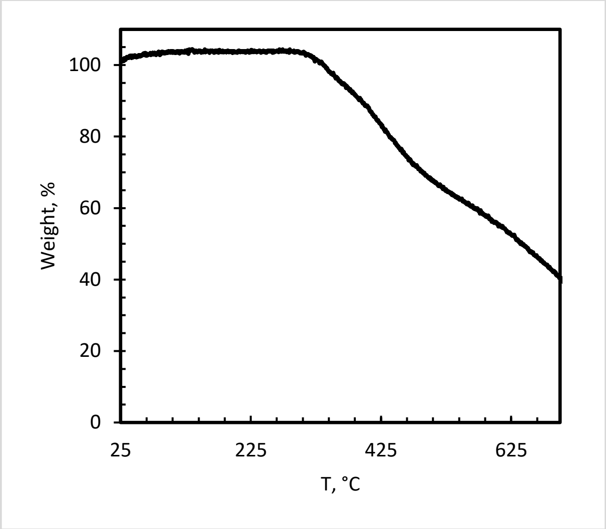

Figure S135. Mass loss as a function of temperature for polyketone **PAAK-7** obtained in the atmosphere of hydrogen.

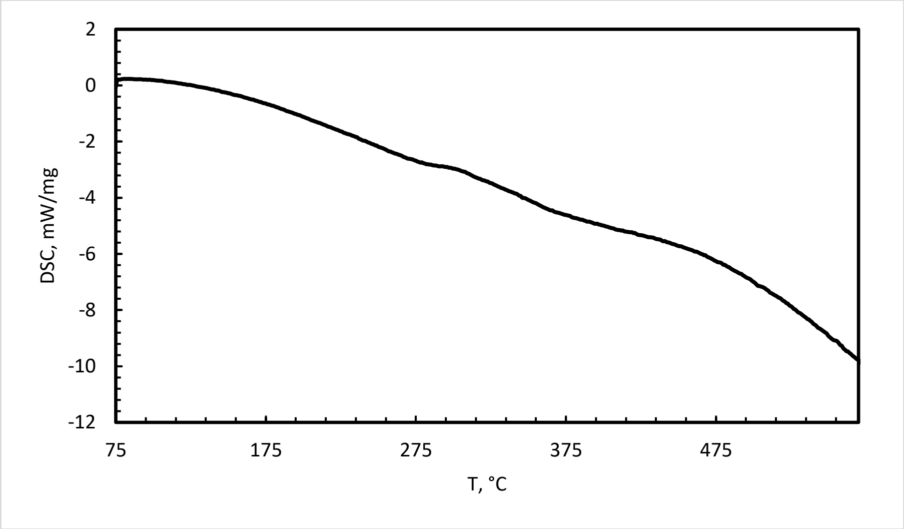

Figure S136. DSC trace corresponding to **PAAK-7** obtained in the atmosphere of hydrogen.

*Synthesis of **PAAK-9** in the presence of hydrogen atmosphere.*
A 100 mL ampoule equipped with a J-Young's valve was charged with pre-catalyst **1** (2.5 mg, 0.005 mmol, 1 mol%), 1,4-cyclohexanedimethanol (72 mg, 0.5 mmol), 4-acetylphenyl ether (127 mg, 0.5 mmol) and Cs_2CO_3 (16.5 mg, 0.05 mmol, 10 mol%). *tert*-Amyl alcohol (5 mL) was added and the flask was sealed under a hydrogen atmosphere before heating to 140 °C for 18 h with stirring. After this period, the reaction vessel was allowed to cool to room temperature. To the resulting mixture, 5 mL of 1 M HCl was added and the flask had been heated at 90 °C for 1 h. The white precipitate (178 mg, 98% yield) was filtered and dried under reduced pressure at 120 °C.
IR (ATR-FTIR, cm^{-1}): ν 2916w (C-H), 2853w (C-H), 1674m (C=O), 1587m (C=C), 1497m, 1234s, 1163s, 835m.
TGA: Td = 384 °C

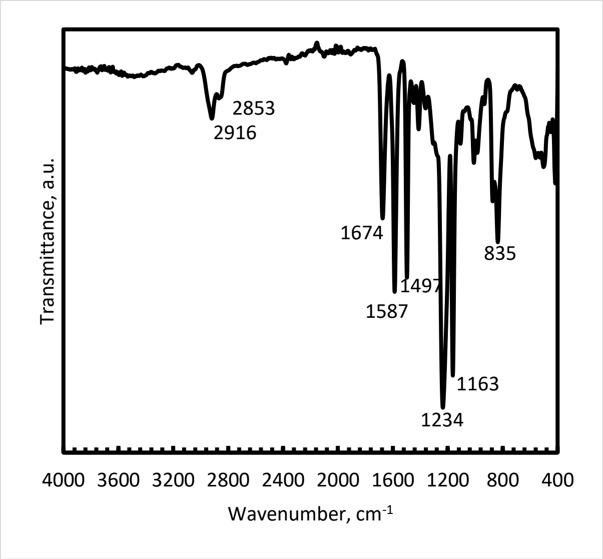

Figure S137. Infrared spectrum (ATR-FTIR) of polyketone **PAAK-9** obtained in the atmosphere of hydrogen.

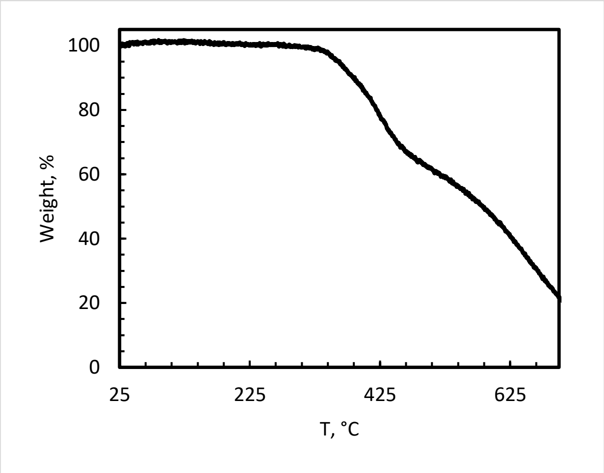

Figure S138. Mass loss as a function of temperature for polyketone **PAAK-9** obtained in the atmosphere of hydrogen.

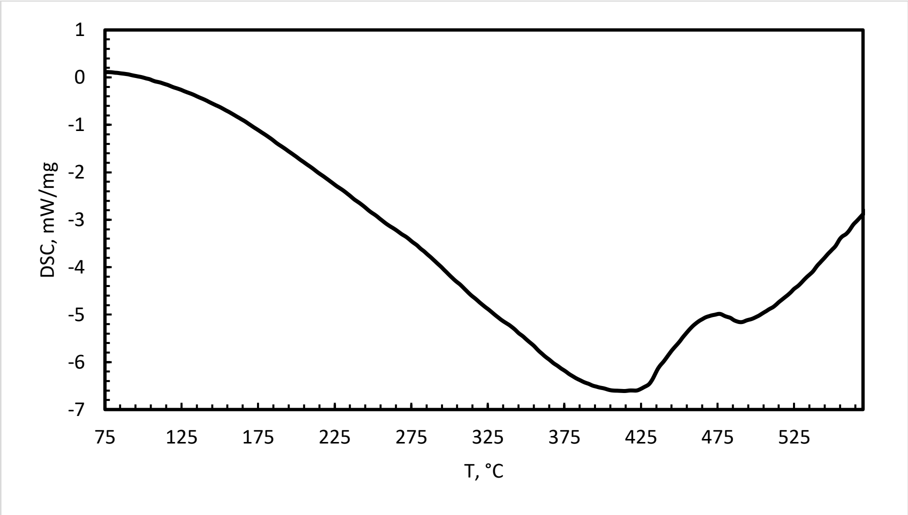

Figure S139. DSC trace corresponding to **PAAK-9** obtained in the atmosphere of hydrogen.

1.9 Catalyst reuse experiments.

Catalyst reuse after synthesis of PAAK-7.

A 100 mL ampoule equipped with a J-Young's valve was charged with pre-catalyst **1** (2.5 mg, 0.005 mmol, 1 mol%) and Cs_2CO_3 (16.5 mg, 0.05 mmol, 10 mol%), 1,4-benzenedimethanol (69 mg, 0.5 mmol) and 4-acetylphenyl ether (127 mg, 0.5 mmol). The flask was sealed under an argon atmosphere and *tert*-amyl alcohol (5 mL) was added before heating to 140 °C for 2 h with stirring. After this period, the reaction vessel was allowed to cool to room temperature. The reaction mixture consisted of a white precipitate (175 mg, 89%) and a yellowish solution. The ampule was connected to the Schlenk line and the yellowish solution was transferred to another 100 mL ampule charged with fresh portion of 1,4-benzenedimethanol (69 mg, 0.5 mmol) and 4-acetylphenyl ether (127 mg, 0.5 mmol) under argon. The freshly charged ampule was sealed and heating to 140 °C for 2 h with stirring. After this period, the reaction vessel was allowed to cool to room temperature. No precipitate was observed after the second iteration.

Catalyst reuse after synthesis of PAAK-7 with addition of base.

A 100 mL ampoule equipped with a J-Young's valve was charged with pre-catalyst **1** (2.5 mg, 0.005 mmol, 1 mol%) and Cs_2CO_3 (16.5 mg, 0.05 mmol, 10 mol%), 1,4-benzenedimethanol (69 mg, 0.5 mmol) and 4-acetylphenyl ether (127 mg, 0.5 mmol). The flask was sealed under an argon atmosphere and *tert*-amyl alcohol (5 mL) was added before heating to 140 °C for 2 h with stirring. After this period, the reaction vessel was allowed to cool to room temperature. The reaction mixture consisted of a white precipitate (161 mg, 90%) and a yellowish solution. The ampule was connected to the Schlenk line and the yellowish solution was transferred to another 100 mL ampule charged with fresh portion of Cs_2CO_3 (16.5 mg, 0.05 mmol, 10 mol%), 1,4-benzenedimethanol (69 mg, 0.5 mmol) and 4-acetylphenyl ether (127 mg, 0.5 mmol) under argon. The freshly charged ampule was sealed and heating to 140 °C for 2 h with stirring. After this period, the reaction vessel was allowed to cool to room temperature. To the resulting mixture, 5 mL of 1 M HCl was added and the flask had been heated at 90 °C for 1 h. The precipitate was filtered and dried under reduced pressure at 120 °C giving the product (98 mg, 55% yield) as a white solid.

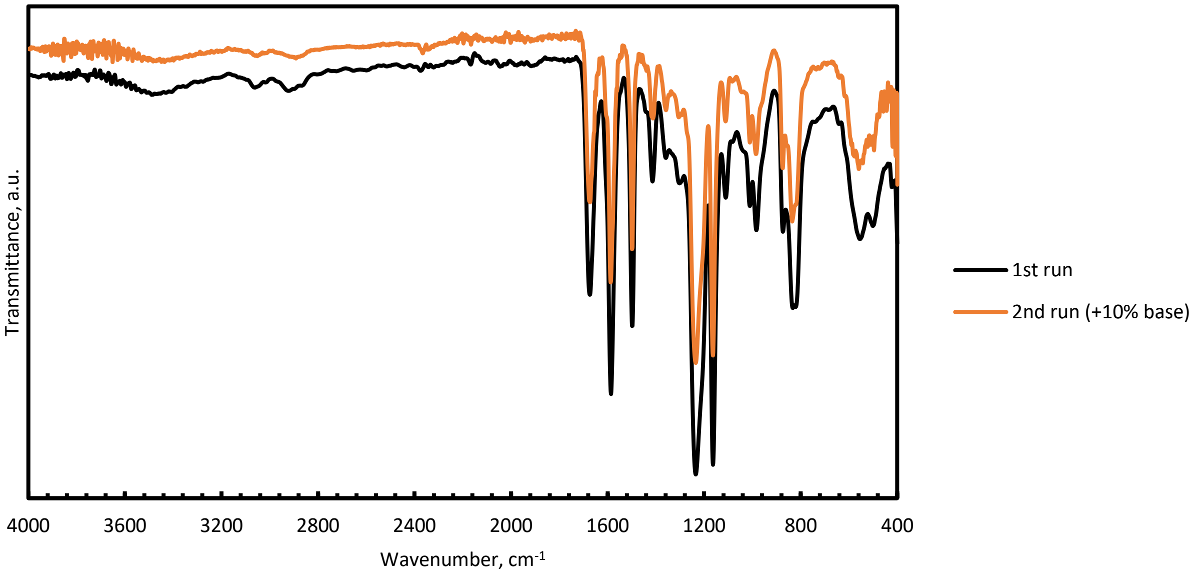

Figure S140. Infrared spectrum (ATR-FTIR) of **PAAK-7** obtained by fresh catalyst (black) and reused reaction solution with addition of 10% base (orange).

1.10 Headspace gas analysis from the synthesis of polyketone PAAK-7.

A 100 mL ampoule equipped with a J-Young's valve was charged with pre-catalyst **1** (2.5 mg, 0.005 mmol, 1 mol%) and Cs_2CO_3 (16.5 mg, 0.05 mmol, 10 mol%), 1,4-benzenedimethanol (69 mg, 0.5 mmol) and 4-acetylphenyl ether (127 mg, 0.5 mmol). The flask was sealed under an argon atmosphere and *tert*-amyl alcohol (5 mL) was added before heating to 140 °C for 2 h with stirring. After this period, the reaction vessel was allowed to cool to room temperature. A sample of the headspace was extracted using a gas tight syringe and analysed using GC-TCD. It was demonstrated that the injected gas consisted mainly from Hydrogen (from the reaction itself) and Nitrogen (from the reaction inert atmosphere) with some Oxygen originated from the atmosphere during sample preparation and injection (see Figure below).

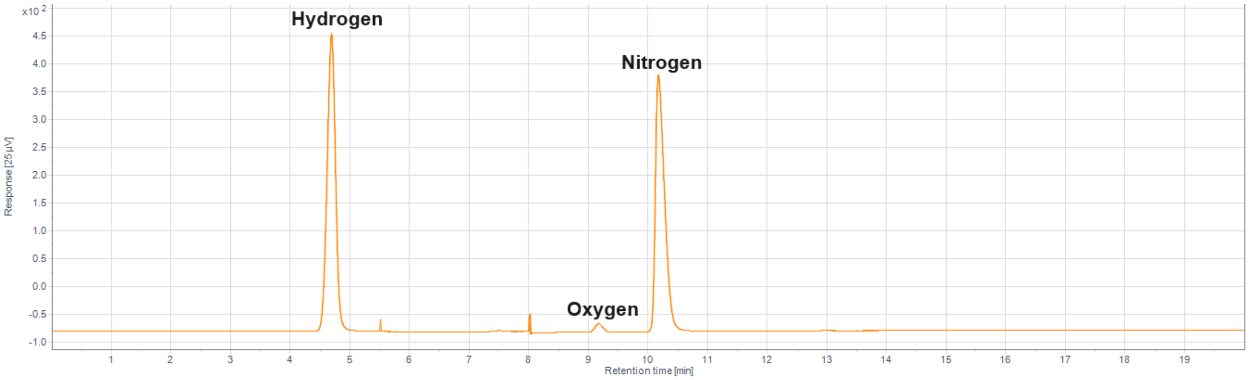

Figure S141. GC-TCD chromatograph of the reaction headspace from synthesis of **PAAK-7**.

1.11 Hydrogenative depolymerization of polyethyleneterephtalate (PET).

Table S2. Tested conditions for hydrogenative depolymerization of PET.[a]

Entry	Amount of catalyst	Catalyst	Base	Amount of base	Conversion[b]	Yield of 1,4-benzene-dimethanol[c]
1	1%	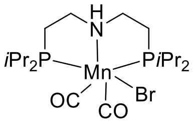	KO*t*Bu	10%	0%	0%
2	3%	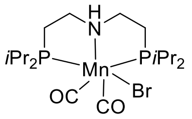	KO*t*Bu	30%	0%	0%
3	2%	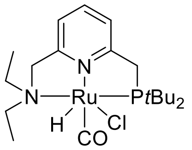	KO*t*Bu	10%	quant.	97%

[a] Polyethylene terephthalate (192.2 mg, 1 mmol, 1 eq., taken from a plastic bottle), a catalyst (1–3%) and KOtBu (10–30%), were weighed under air, placed into an 8 mL glass ampoule, a stir bar was added, and the ampule was sealed with a cap, containing a septum. The ampule was backfilled with argon and 5 mL of anhydrous *tert*-amyl alcohol was added via syringe. The ampules were placed inside a 150 mL autoclave with some metal beans to ensure thermocunductivity. The autoclave was purged with argon, then sealed, purged with H_2, pressurized with 50 bar of H_2, and placed in an oil bath. The reaction was stirred at 140 °C for 88 hours. After that, the autoclave was cooled down to room temperature in an ice bath and carefully vented to atmosphere. [b] Conversion was calculated as 100% minus amount of unreacted PET flakes. [c] Yield was calculated via ^{1}H NMR employing mesitylene as internal standard. Hence, 200-300 mg of the reaction solution and 10 mg mesitylene were weighed in a NMR tube followed by the addition of methanol-d^4 (0.3 mL).

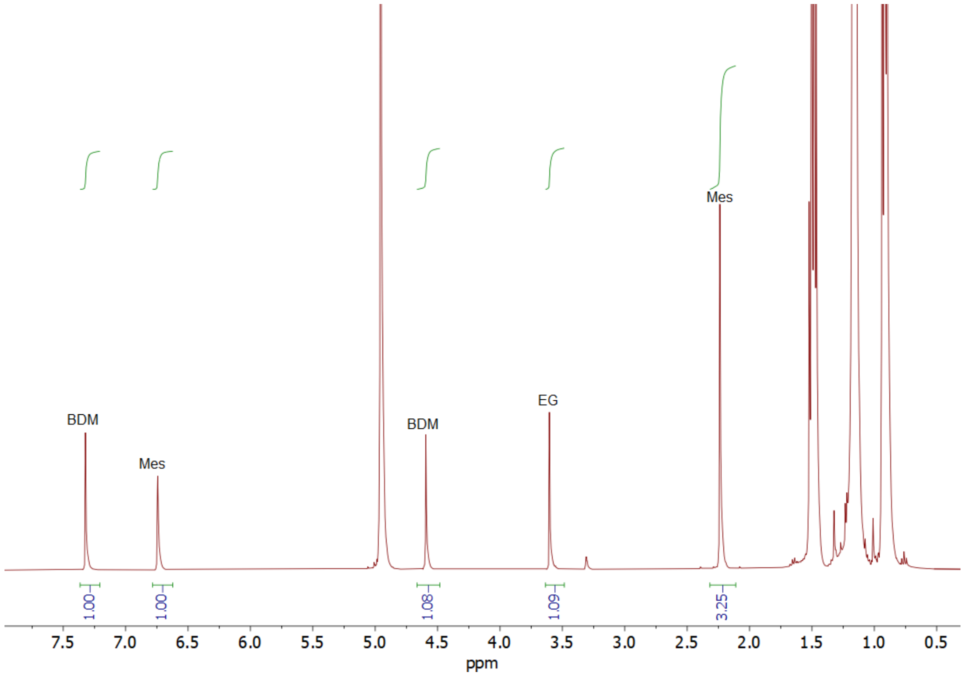

Figure S142. ^{1}H NMR spectrum of reaction mixture from Entry 3 in Table S2 in methanol-d^4 at room temperature. BDA – 1,4-benzenedimethanol, EG – ethylene glycol, Mes – mesitylene. The remaining signals correspond to *tert*-amyl alcohol and methanol-d^4.

1.12 Hydrogenation of trans-Chalcone by transfer hydrogenation.

A 100 mL ampoule equipped with a J-Young's valve was charged with **1** (2.5 mg, 0.005 mmol, 1 mol%), 1,4-benzenedimethanol (69 mg, 0.5 mmol), *trans*-chalcone (104 mg, 0.5 mmol) and Cs_2CO_3 (16.5 mg, 0.10 mmol, 10 mol%). The flask was refiled with argon and *tert*-amyl alcohol (5 mL) was added. The flask was sealed and heated 140 °C for 2 h with stirring. After this period, the reaction vessel was allowed to cool to room temperature and the resulting mixture was analysed with GC-MS and NMR (with $CDCl_3$ as a solvent) using mesitylene as an internal standard. The yield was estimated to be 46%. The NMR spectrum of 1,3-diphenylpropan-1-one corresponds to the literature data [2].

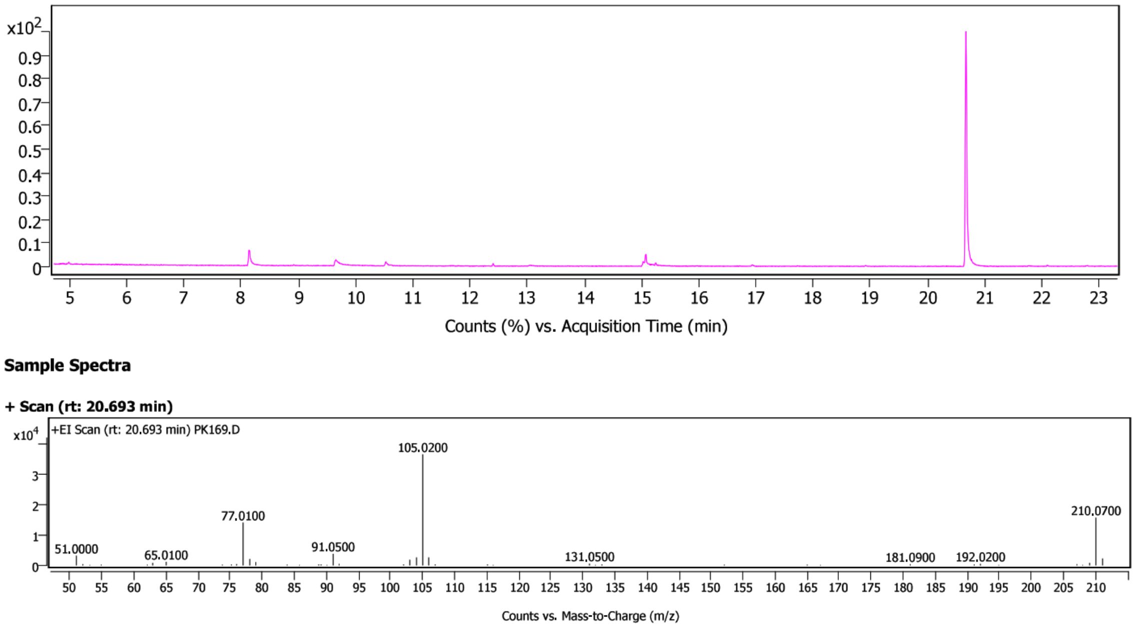

Figure S143. GC chromatograph of the products of transfer hydrogenation of *trans*-chalcone (above) and mass spectrum (EI) of 1,3-diphenylpropan-1-one extracted from the chromatogram.

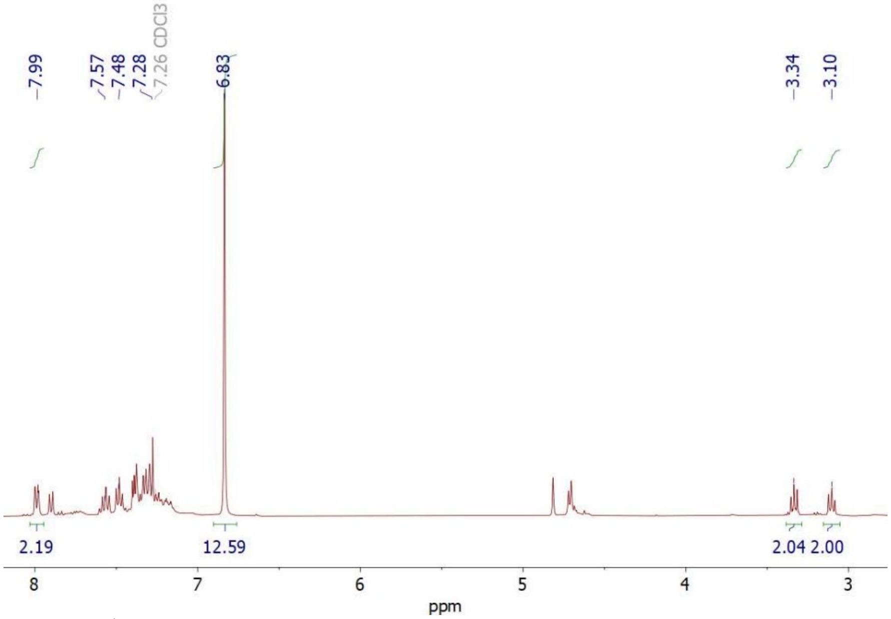

Figure S144. Fragment of ^1H NMR (400 MHz, $CDCl_3$) of the reaction mixture of transfer hydrogenation of *trans*-chalcone in the presence of 1,4-benzenedimethanol.

1.13 Mass spectroscopy investigation of mother liquor from reaction mixtures.

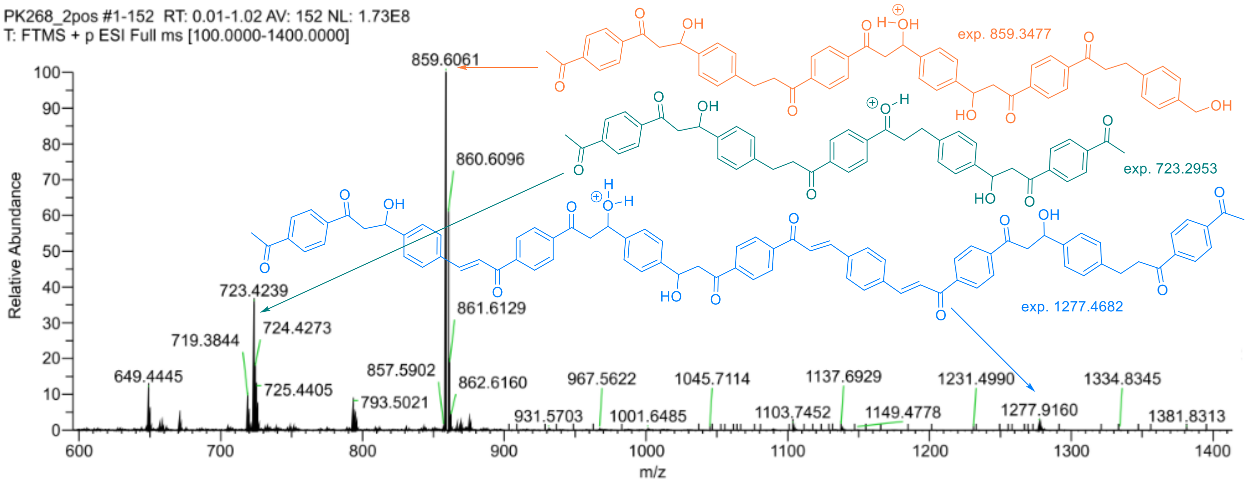

Figure S145. HRMS (EI, MeCN) spectrum for mother liquor from reaction mixture after synthesis of **PAAK-1**. The peak assignment is illustrative and might not represent the precise structure.

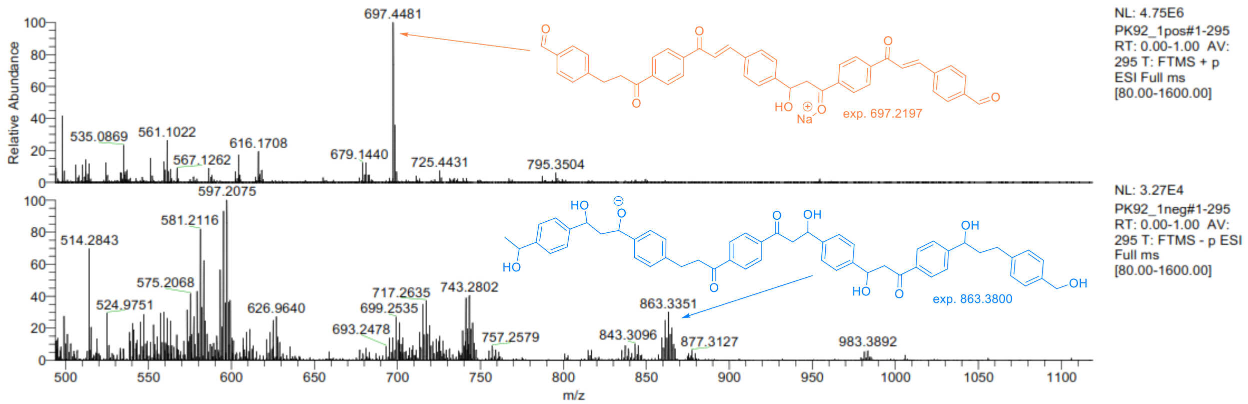

Figure S146. HRMS (EI, MeCN) spectrum for mother liquor from reaction mixture after synthesis of **PAAK-1** corresponding to Entry 10, Table S1. The peak assignment is illustrative and might not represent the precise structure.

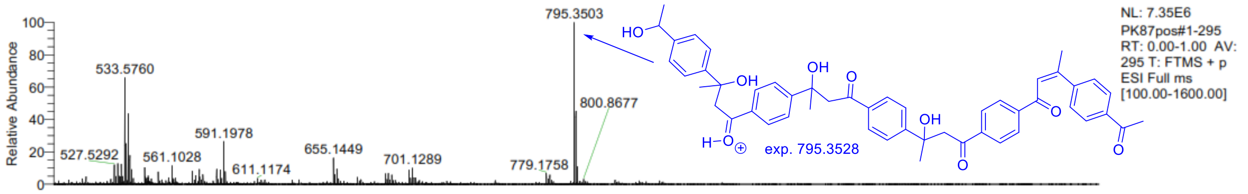

Figure S147. HRMS (EI, MeCN) spectrum for mother liquor from reaction mixture after synthesis of **PAAK-1** corresponding to Entry 18, Table S1. The peak assignment is illustrative and might not represent the precise structure.

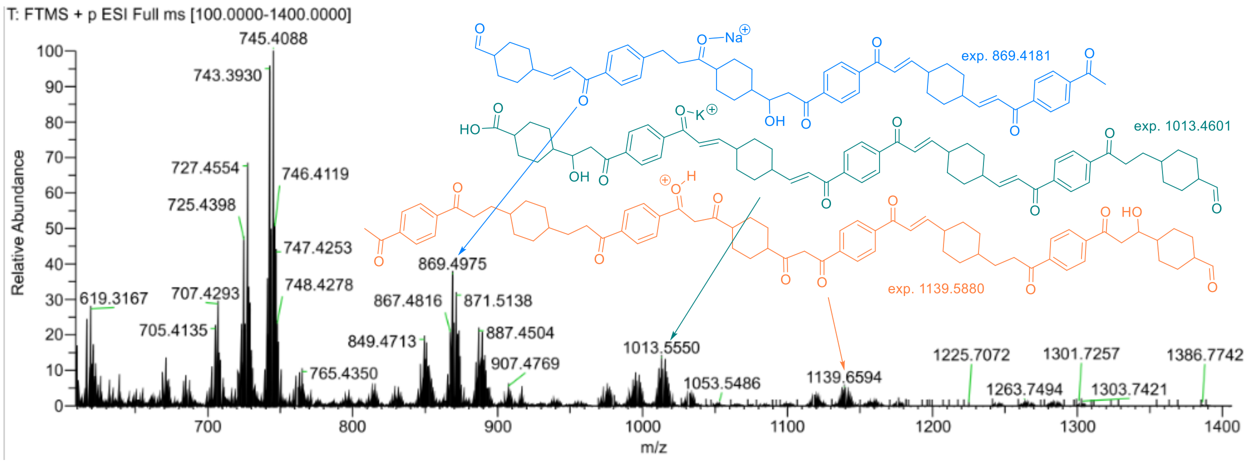

Figure S148. HRMS (EI, MeCN) spectrum for mother liquor from reaction mixture after synthesis of **PAAK-3**. The peak assignment is illustrative and might not represent the precise structure.

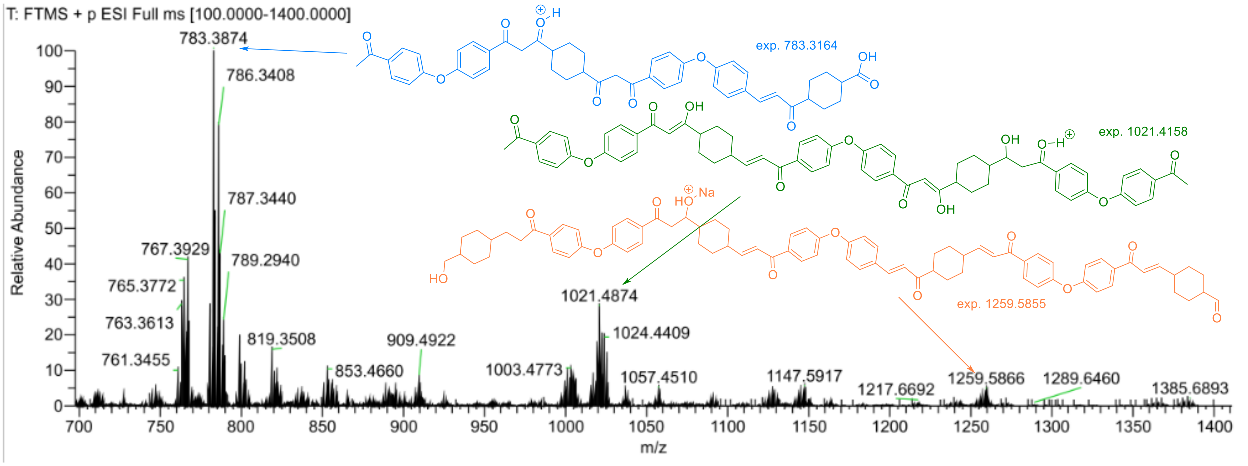

Figure S149. HRMS (EI, MeCN) spectrum for mother liquor from reaction mixture after synthesis of **PAAK-9**. The peak assignment is illustrative and might not represent the precise structure.

1.14 TGA and DSC analysis combined with mass-spectrometry.

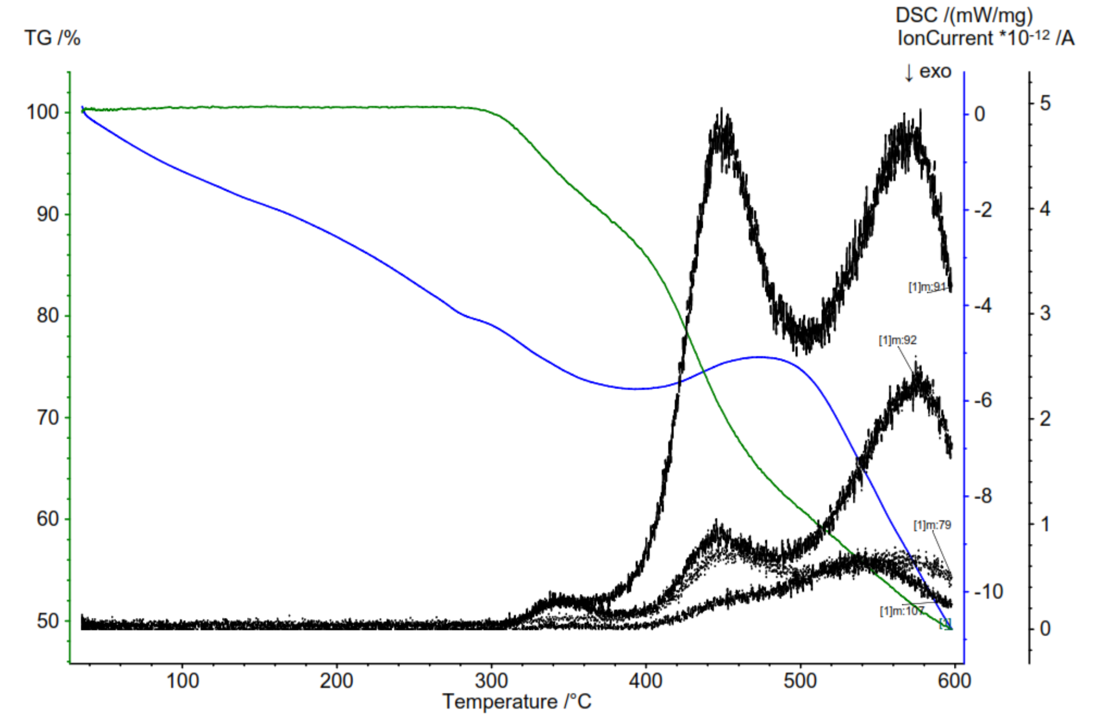

Figure S150. TGA curve (green), DSC trace (blue) and ion current (black) at temperatures 30–600 °C corresponding to **PAAK-7**. Ion current for m=79; 91; 92 and 107 g/mol is shown which corresponds to 1,4-benzenedimethanol-derived fragments in the polymer.

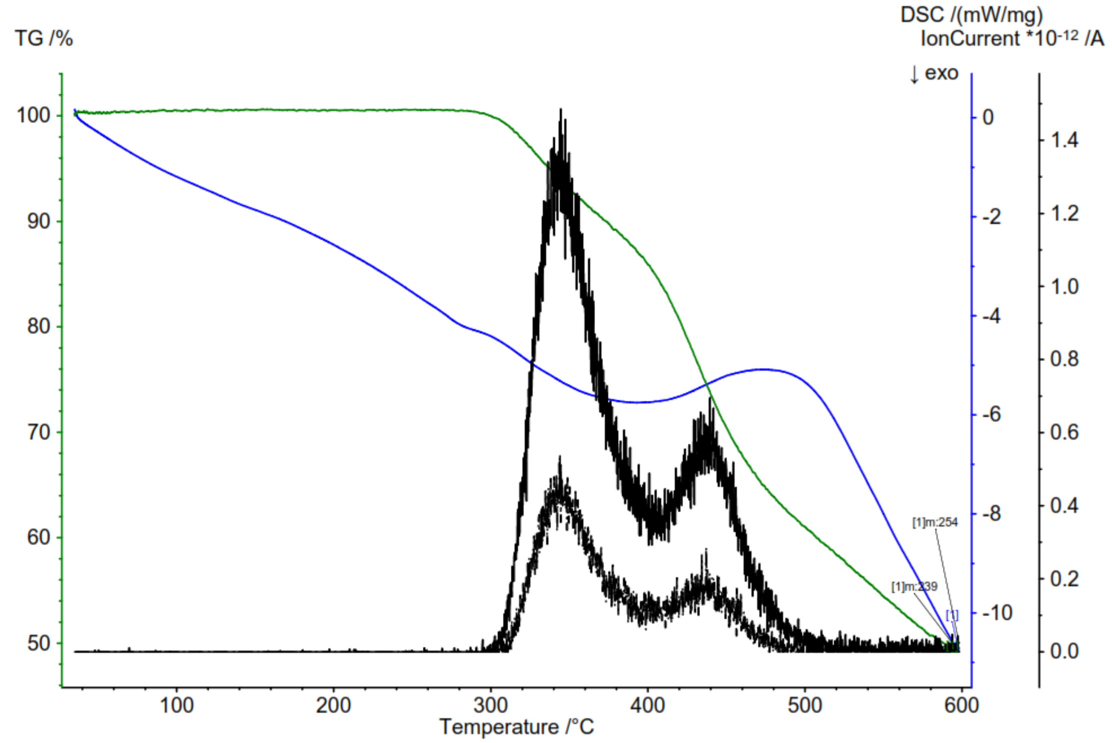

Figure S151. TGA curve (green), DSC trace (blue) and ion current (black) at temperatures 30–600 °C corresponding to **PAAK-7**. Ion current for m=239 and 254 g/mol is shown which corresponds to 4-acetylphenyl ether-derived fragments in the polymer.

1.15 Infrared spectra of starting materials and commercially available compounds.

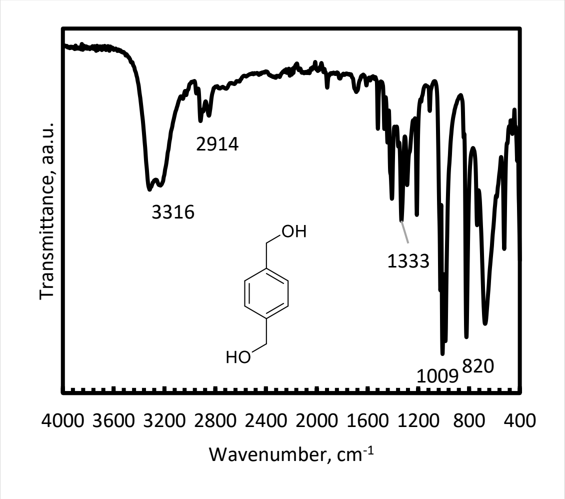

Figure S152. Infrared spectrum (ATR-FTIR) of 1,4-benzenedimethanol.

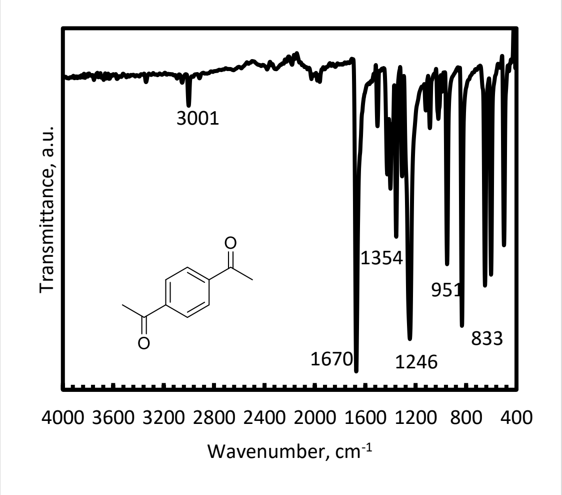

Figure S153. Infrared spectrum (ATR-FTIR) of 1,4-diacetylbenzene.

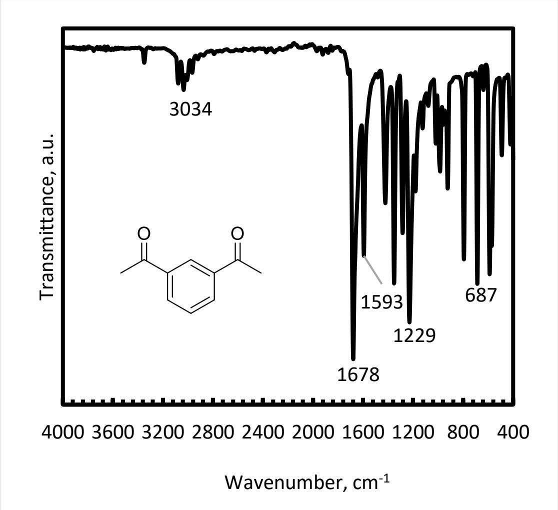

Figure S154. Infrared spectrum (ATR-FTIR) of 1,3-diacetylbenzene.

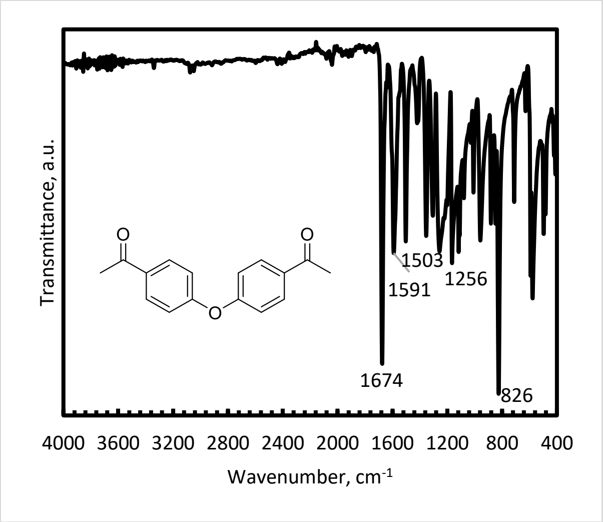

Figure S155. Infrared spectrum (ATR-FTIR) of 4-acetlphenyl ether.

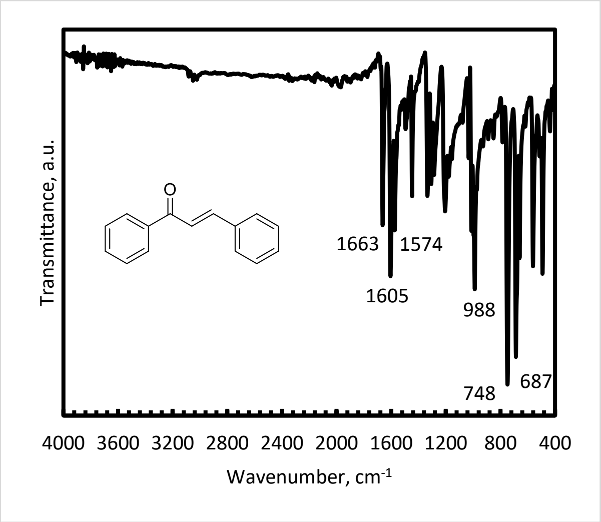

Figure S156. Infrared spectrum (ATR-FTIR) of *trans*-chalcone.

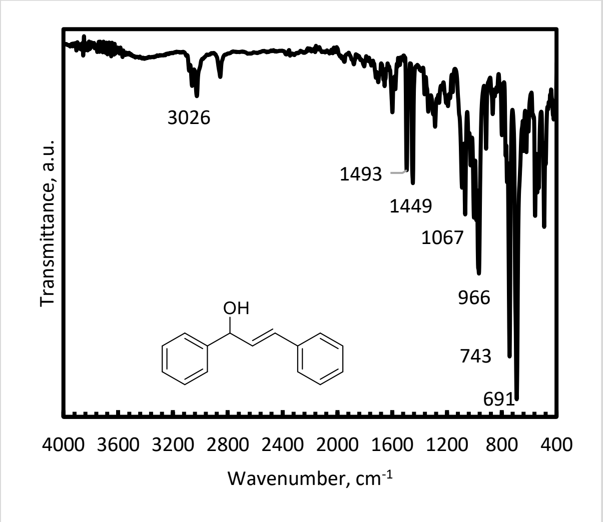

Figure S157. Infrared spectrum (ATR-FTIR) of (*E*)-1,3-diphenylprop-2-en-1-ol.

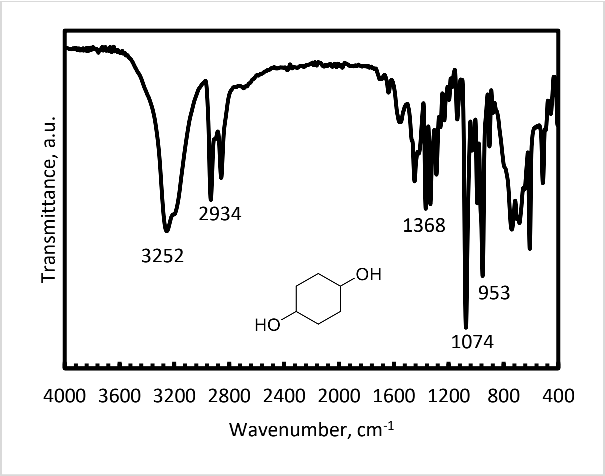

Figure S158. Infrared spectrum (ATR-FTIR) of 1,4-cyclohexanediol.

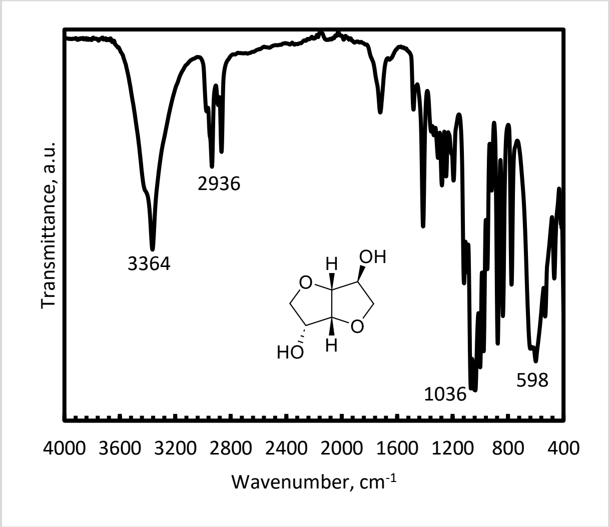

Figure S159. Infrared spectrum (ATR-FTIR) of D-isosorbide.

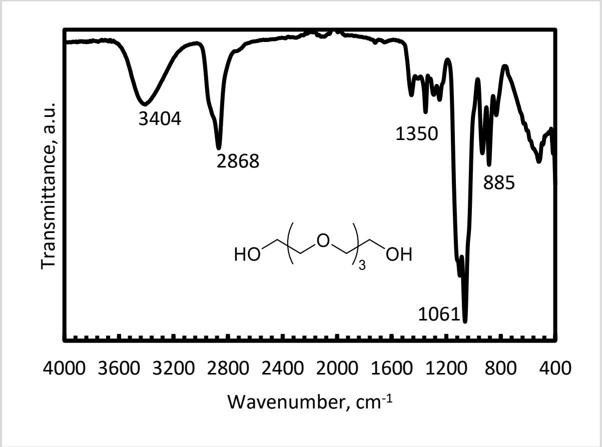

Figure S160. Infrared spectrum (ATR-FTIR) of tetraethylene glycol.

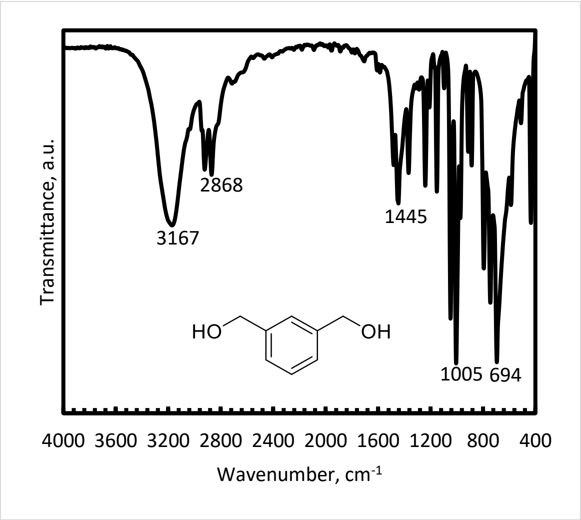

Figure S161. Infrared spectrum (ATR-FTIR) of 1,3-benzenedimethanol.

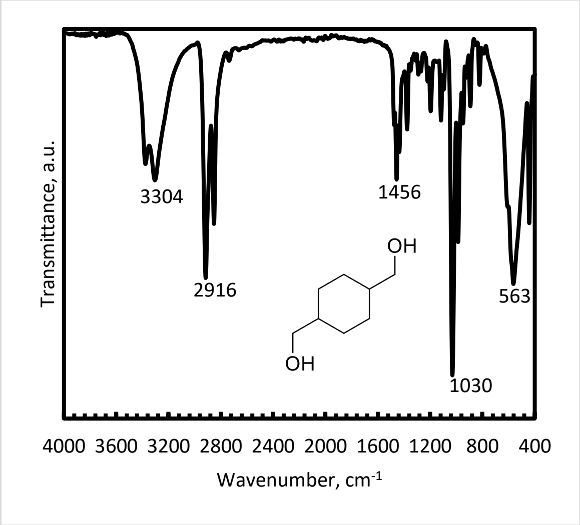

Figure S162. Infrared spectrum (ATR-FTIR) of 1,4-cyclohexanedimethanol.

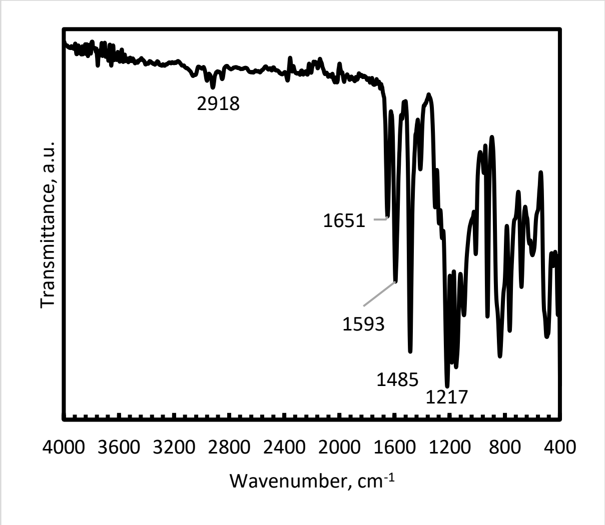

Figure S163. Infrared spectrum (ATR-FTIR) of commercial polymer PEEK.

1.16 Thermal properties and GPC analysis of commercial polyketones.

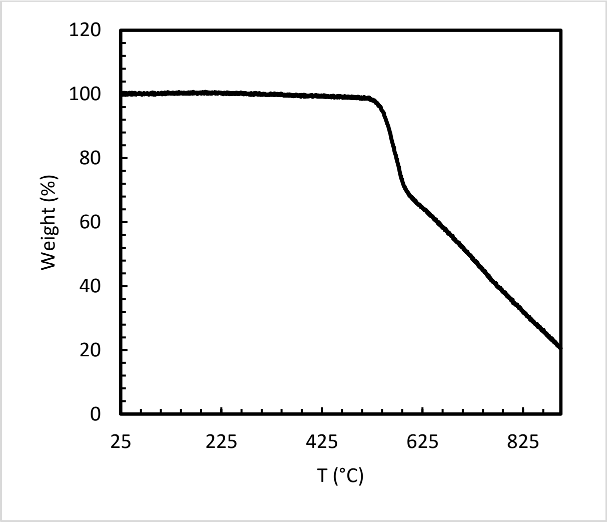

Figure S164. Mass loss as a function of temperature for commercial PEEK.

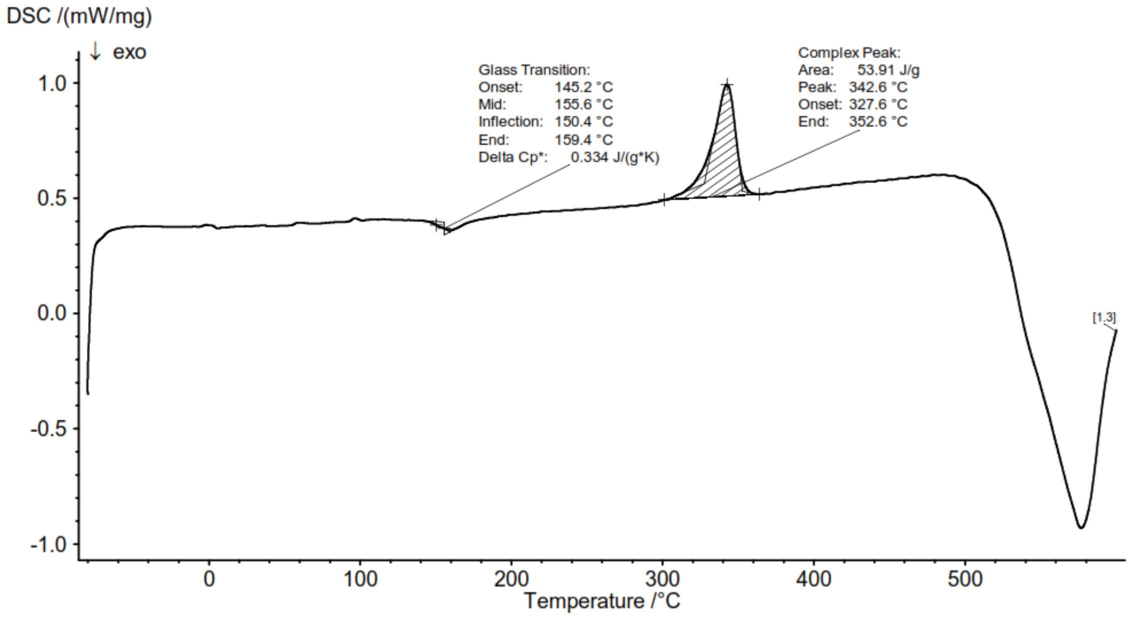

Figure S165. DSC trace corresponding to commercial PEEK.

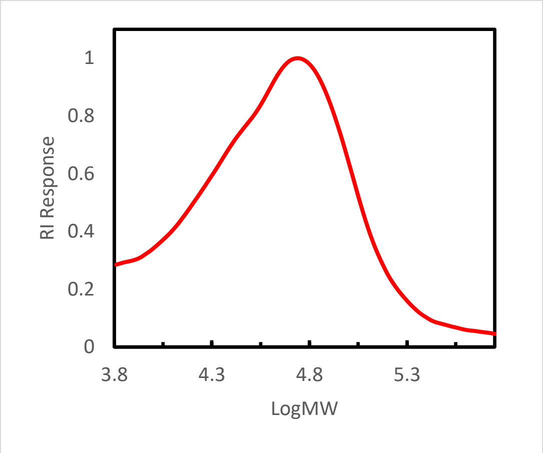

Figure S166. GPC chromatogram corresponding to commercial PEEK.

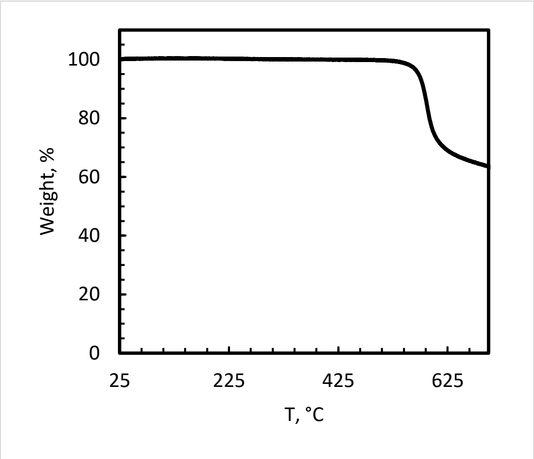

Figure S167. Mass loss as a function of temperature for commercial PEKK.

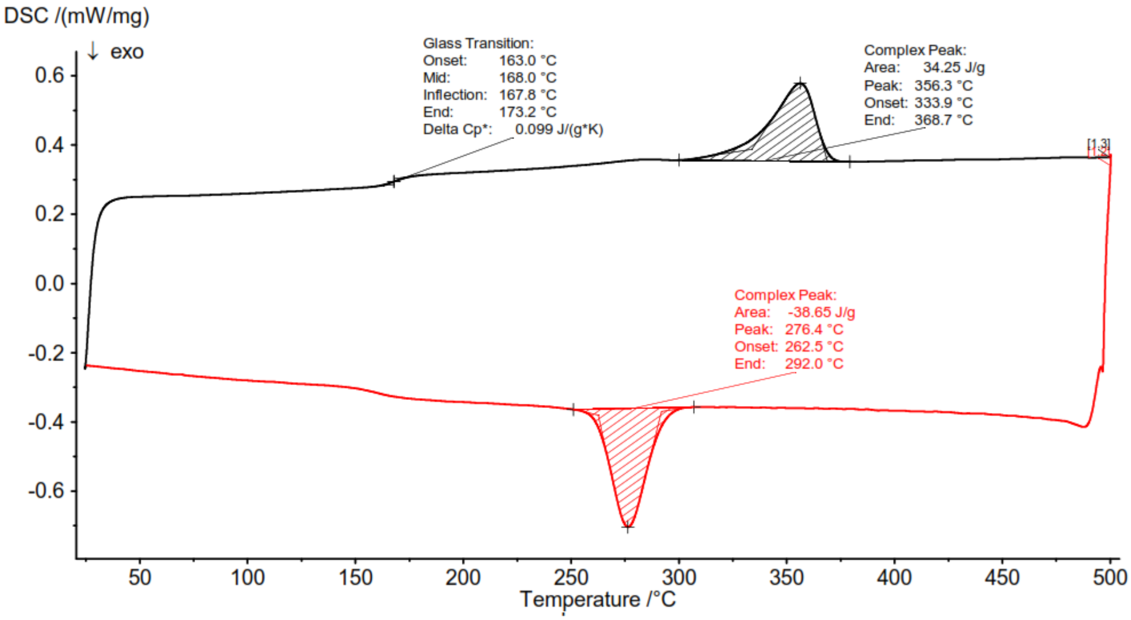

Figure S168. DSC trace corresponding to commercial PEKK.

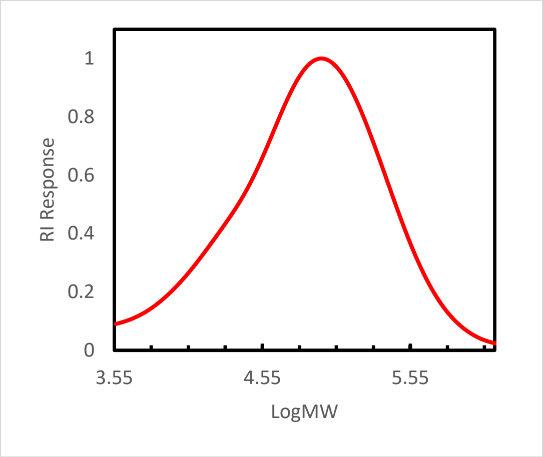

Figure S169. GPC chromatogram corresponding to commercial PEKK.

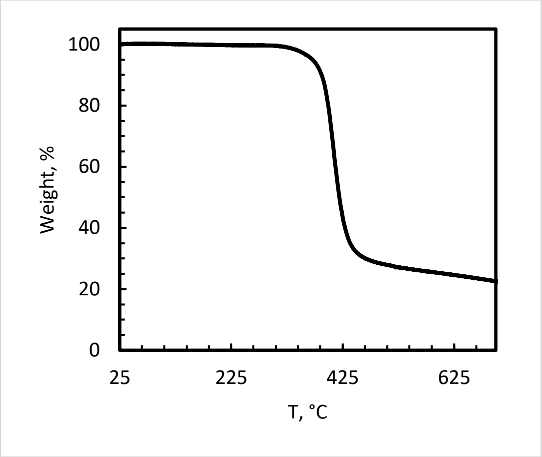

Figure S170. Mass loss as a function of temperature for commercial aliphatic polyketone POK.

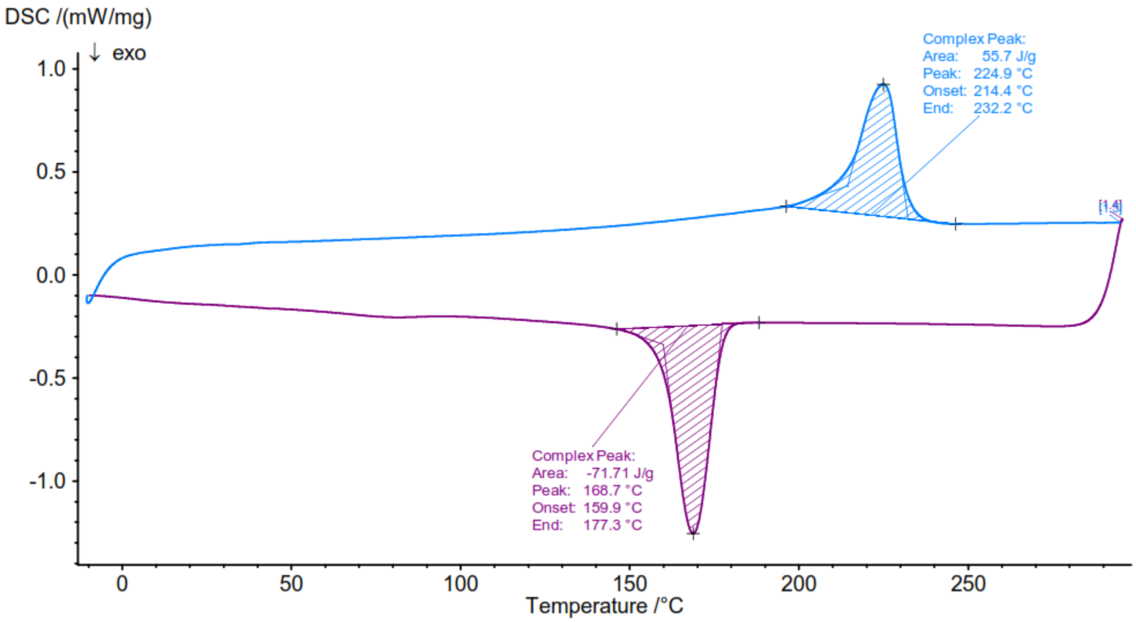

Figure S171. DSC trace corresponding to commercial POK.

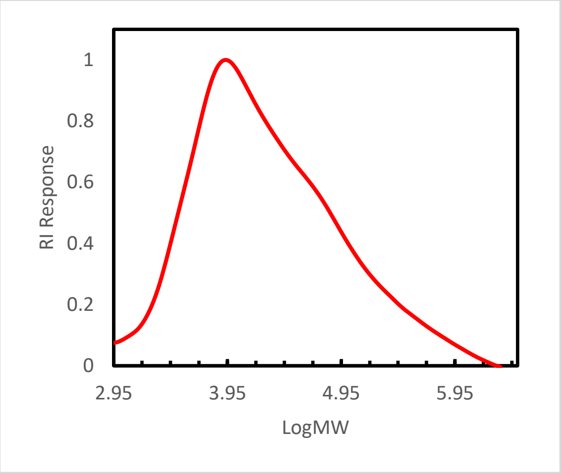

Figure S172. GPC chromatogram corresponding to commercial POK.

Table S3. Thermal properties and molecular weight of the commercial polyketones.

Entry	Polymer	T_d, °C	T_m, °C	T_c, °C	T_g, °C	Mw, kDa	*Đ*
1	POK	387	225	169	n.d.	66.2	7.5
2	PEEK	557	343	276	156	63.3	2.2
3	PEKK	581	356	n.d.	168	126.8	3.4

1.17 Powder XRD patterns of starting materials.

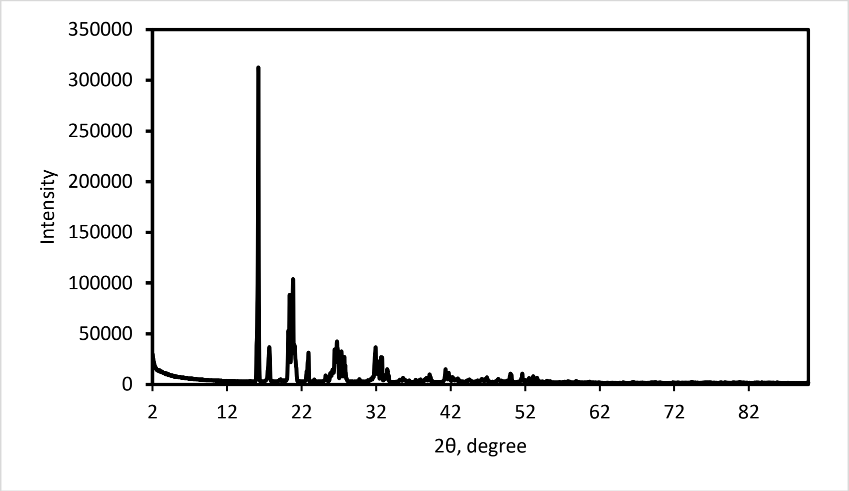

Figure S173. Experimental powder XRD patterns of 1,4-diacetylbenzene.

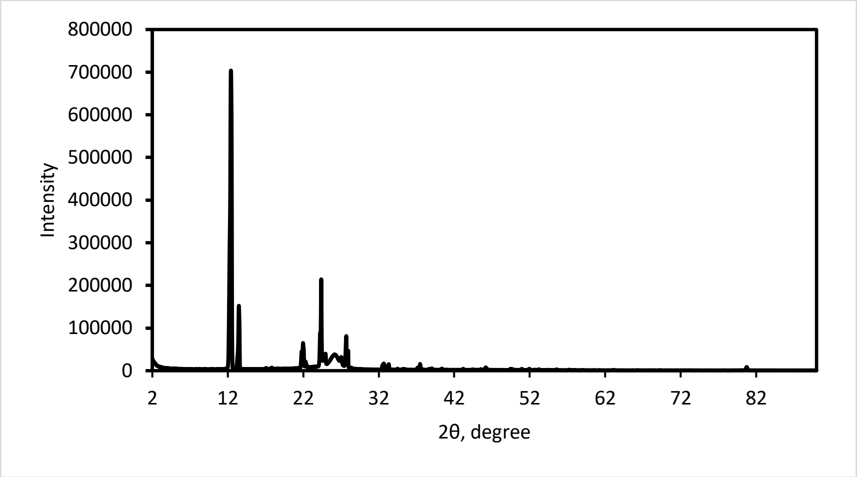

Figure S174. Experimental powder XRD patterns of 1,3-diacetylbenzene.

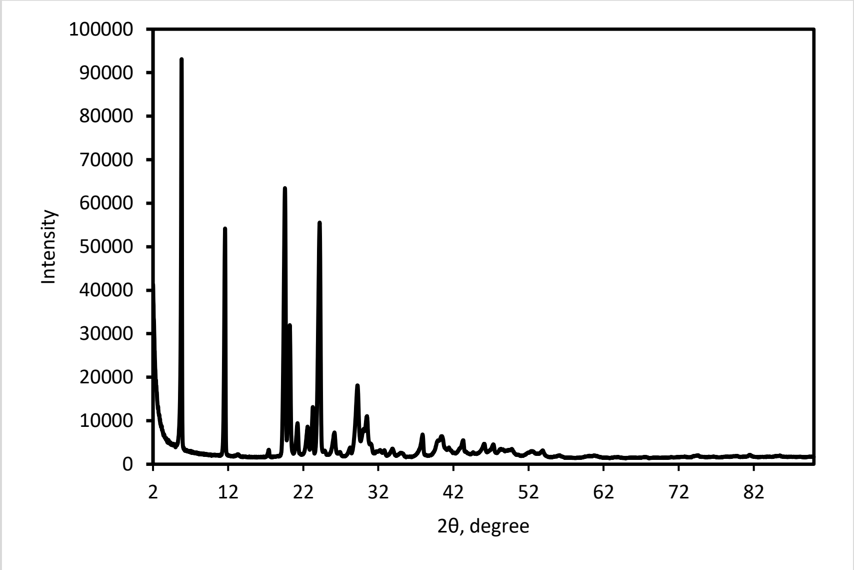

Figure S175. Experimental powder XRD patterns of 4-acetylphenyl ether.

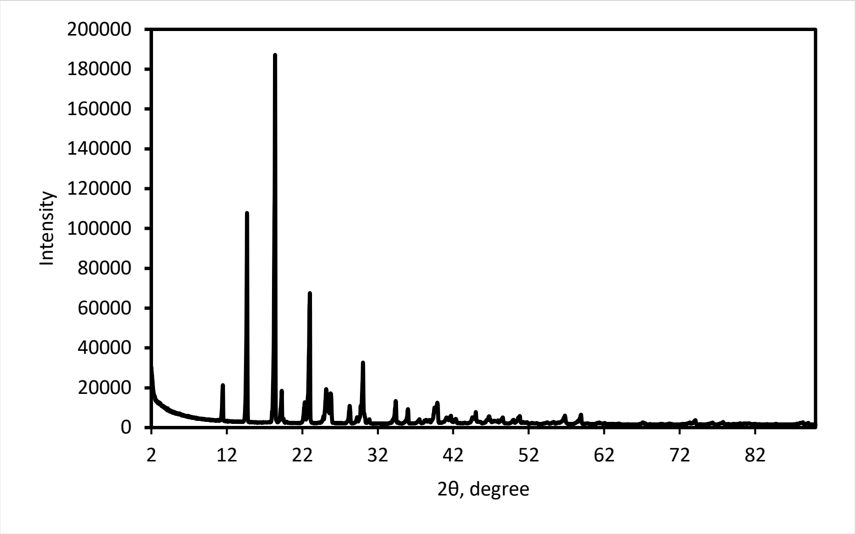

Figure S176. Experimental powder XRD patterns of 1,4-benzenedimethanol.

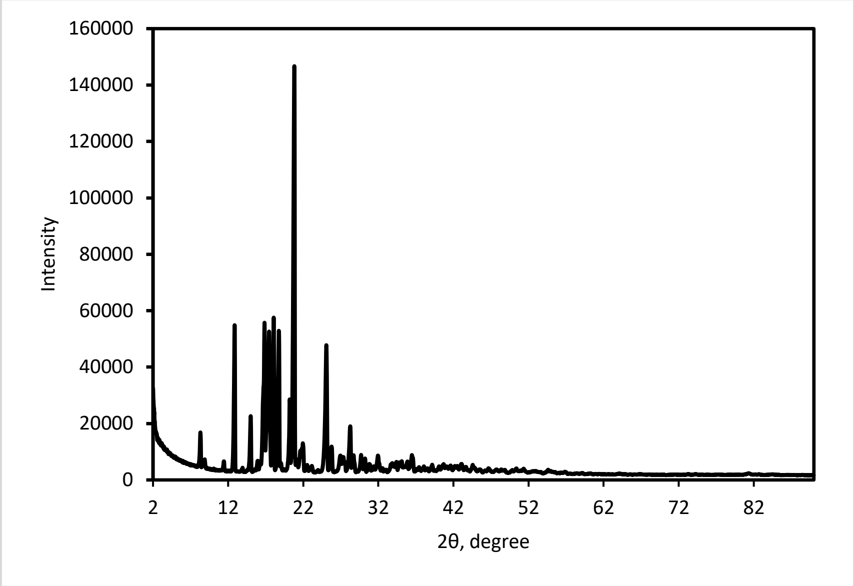

Figure S177. Experimental powder XRD patterns of 1,4-cycloheanedimethanol.

1.18 Scanning Electron Microscopy

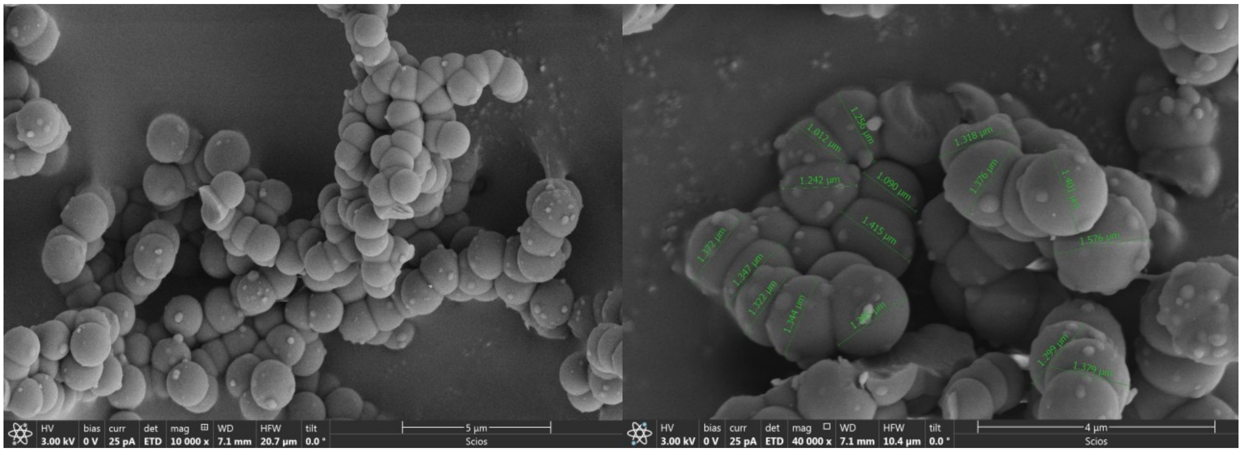

Figure S178. SEM micrographs of polyketone **PAAK-1** corresponding to Table S1, Entry 9.

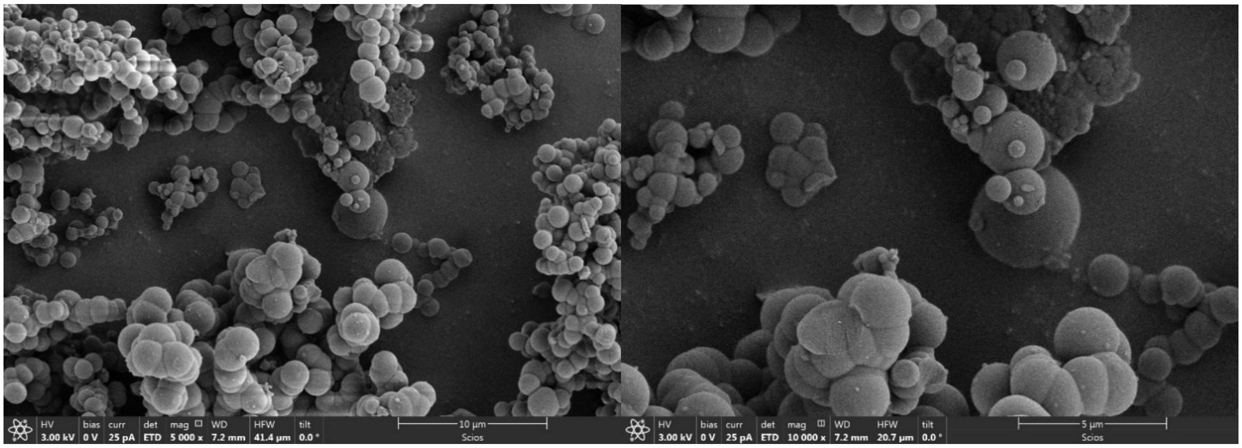

Figure S179. SEM micrographs of polyketone **PAAK-2**.

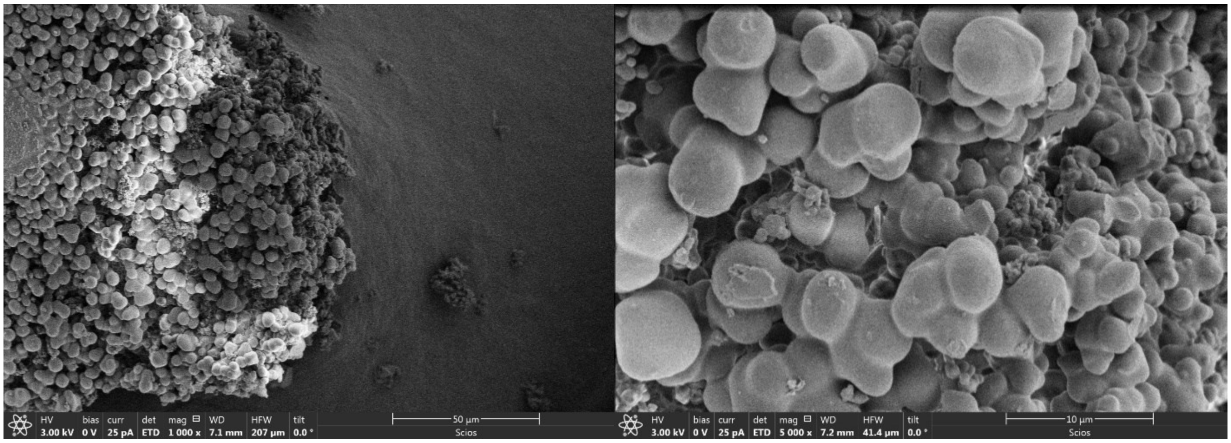

Figure S 180. SEM micrographs of polyketone **PAAK-3**.

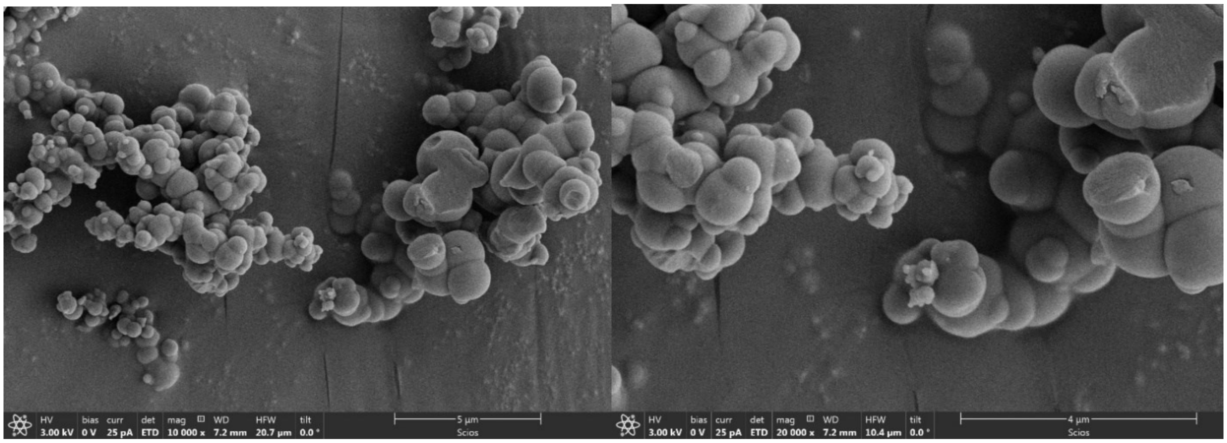

Figure S181. SEM micrographs of polyketone **PAAK-4**.

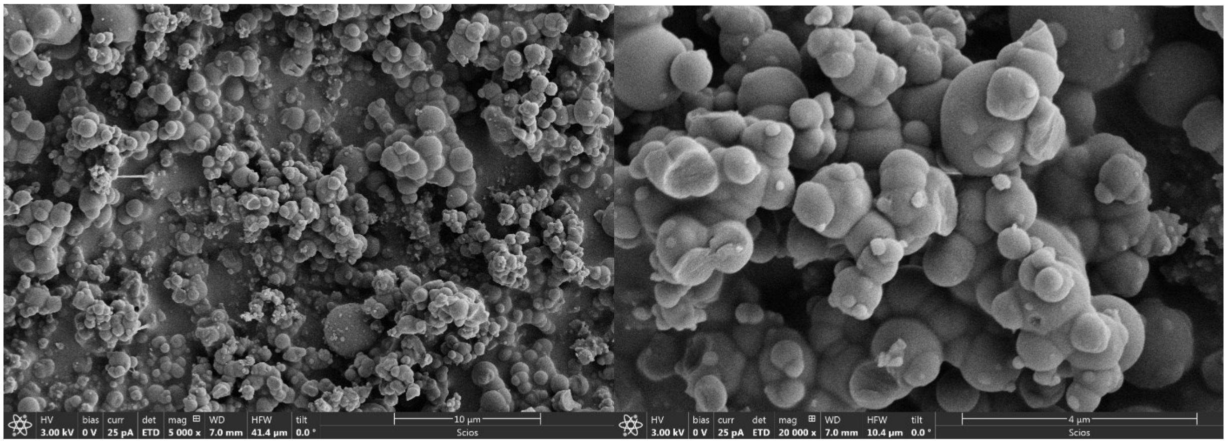

Figure S182. SEM micrographs of polyketone **PAAK-5**.

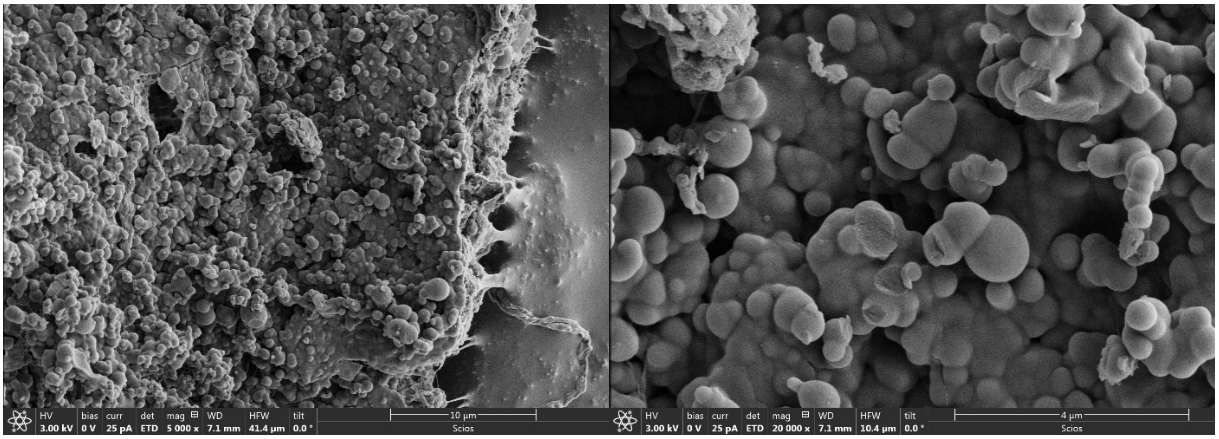

Figure S183. SEM micrographs of polyketone **PAAK-6**.

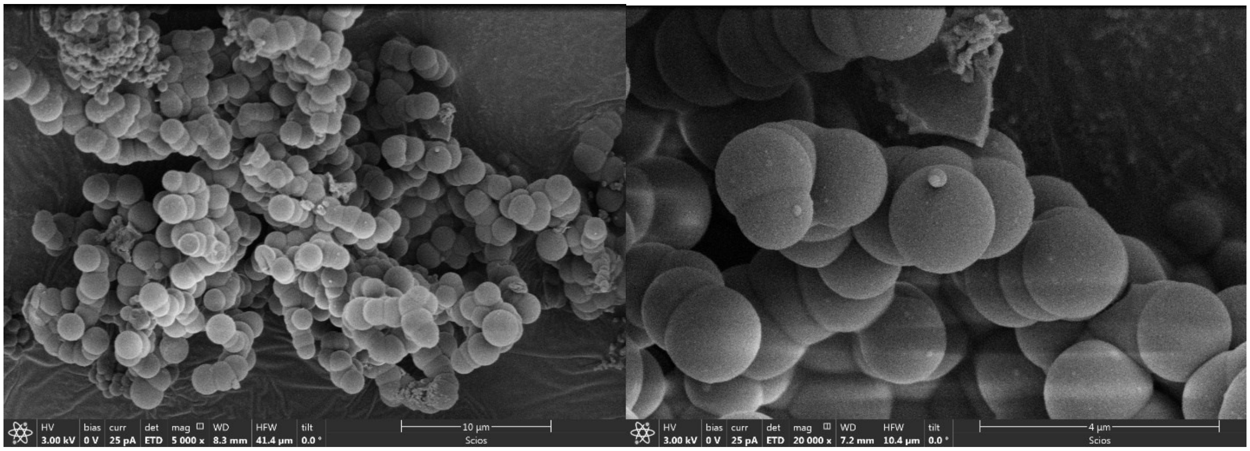

Figure S184. SEM micrographs of polyketone **PAAK-7**.

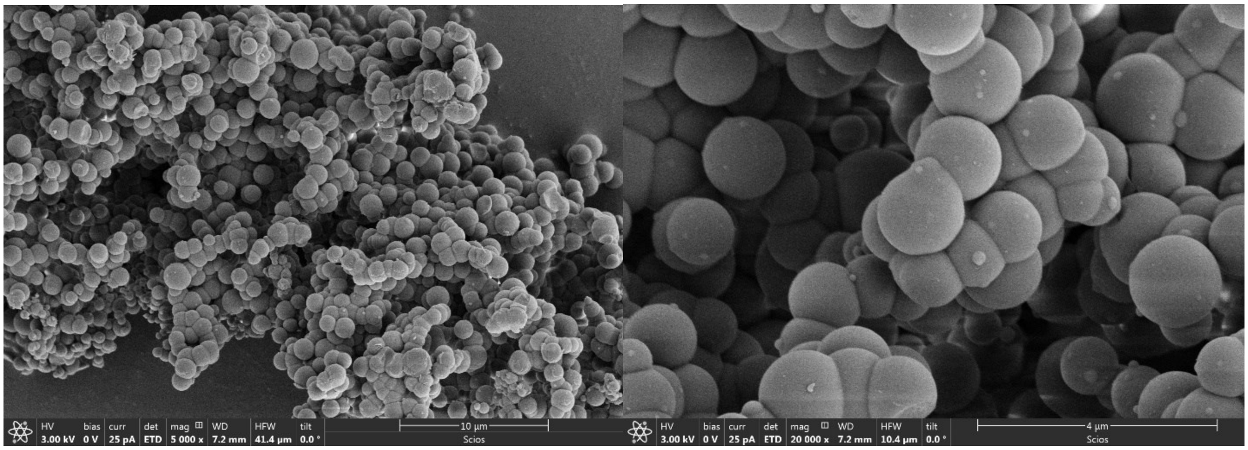

Figure S185. SEM micrographs of polyketone **PAAK-8**.

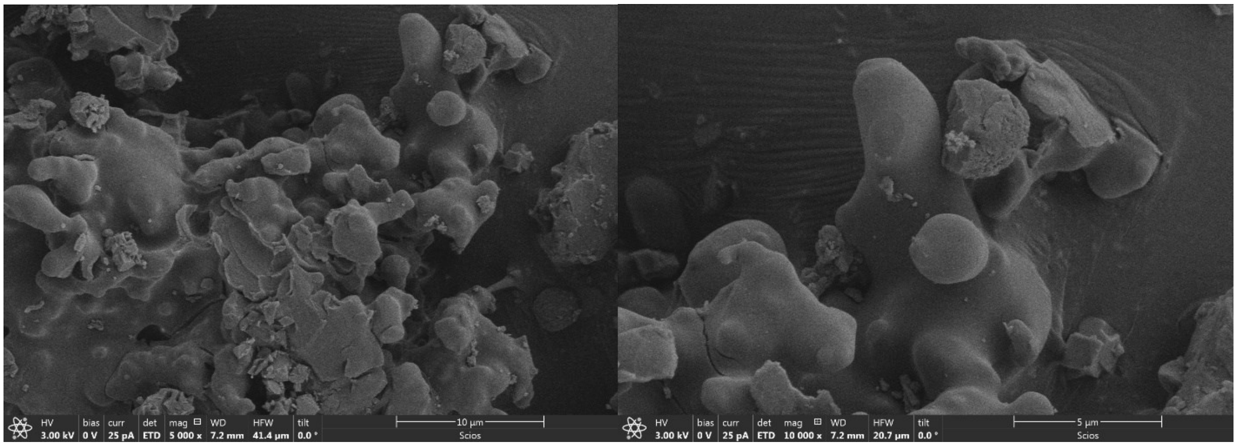

Figure S186. SEM micrographs of polyketone **PAAK-9**.

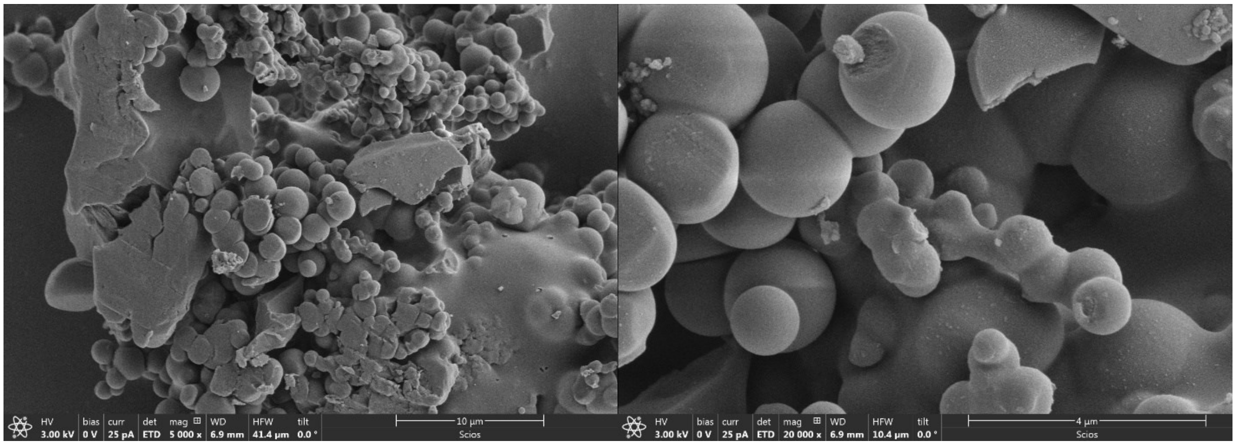

Figure S187. SEM micrographs of polyketone **PAAK-10**.

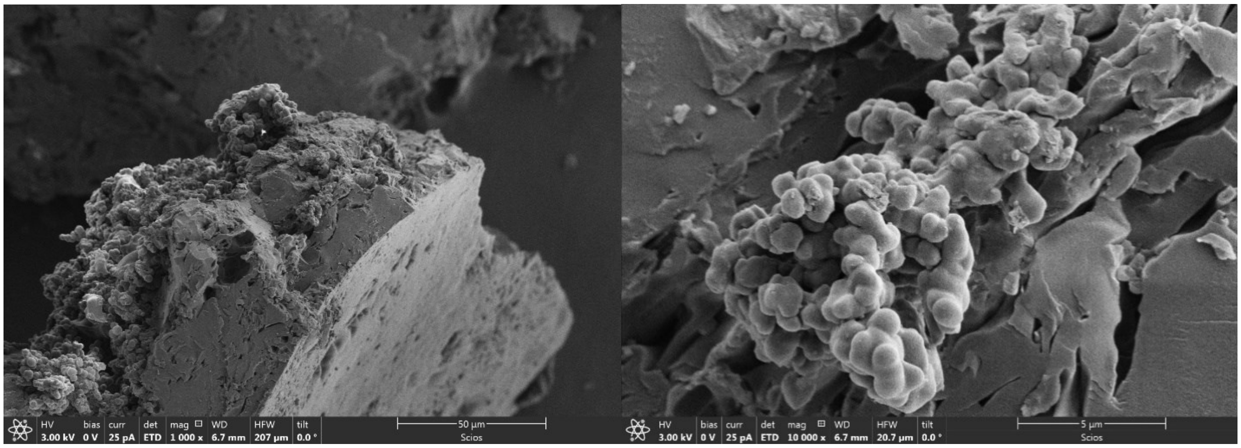

Figure S188. SEM micrographs of polyketone **PAAK-11**.

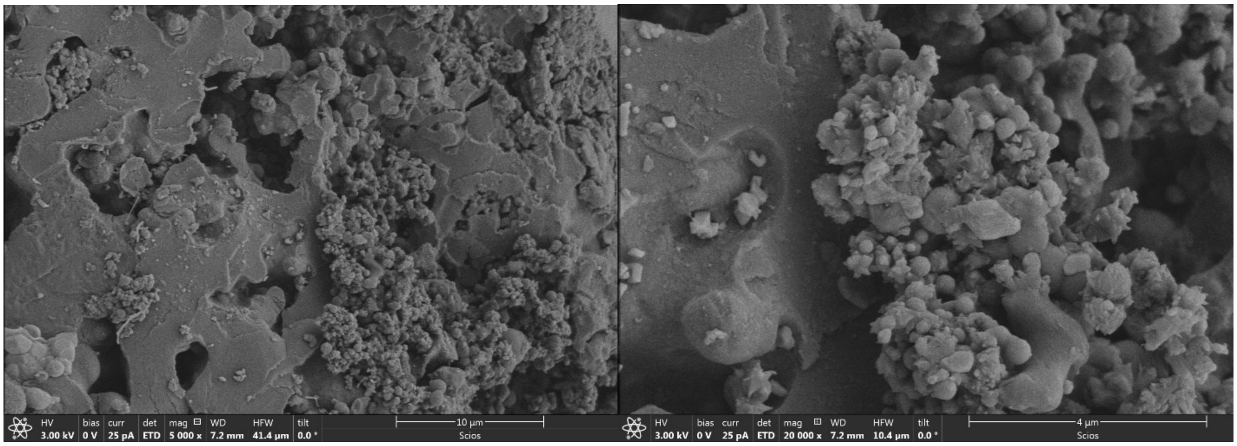

Figure S189. SEM micrographs of polyketone **PAAK-12**.

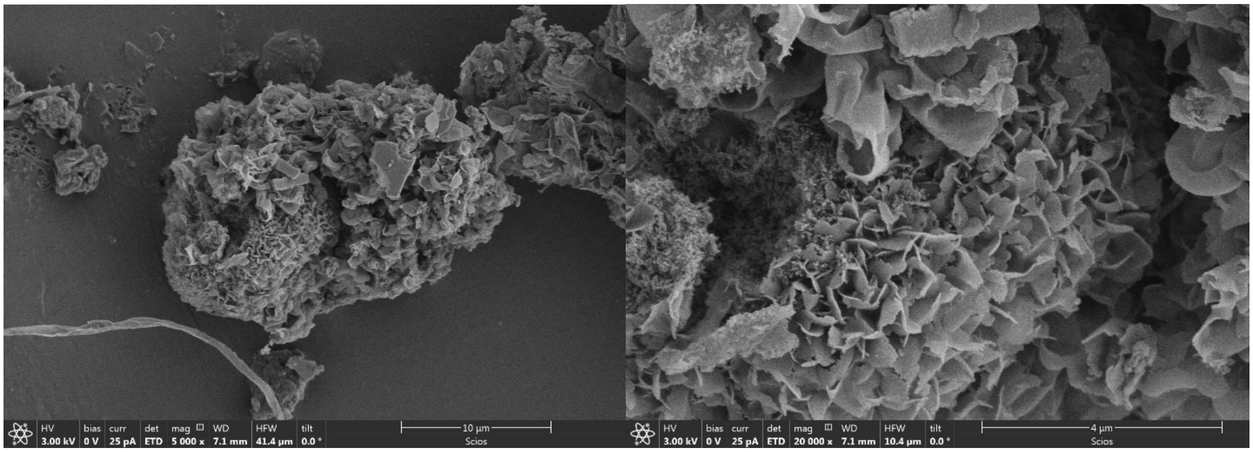

Figure S190. SEM micrographs of polychalcone **PCH-1**.

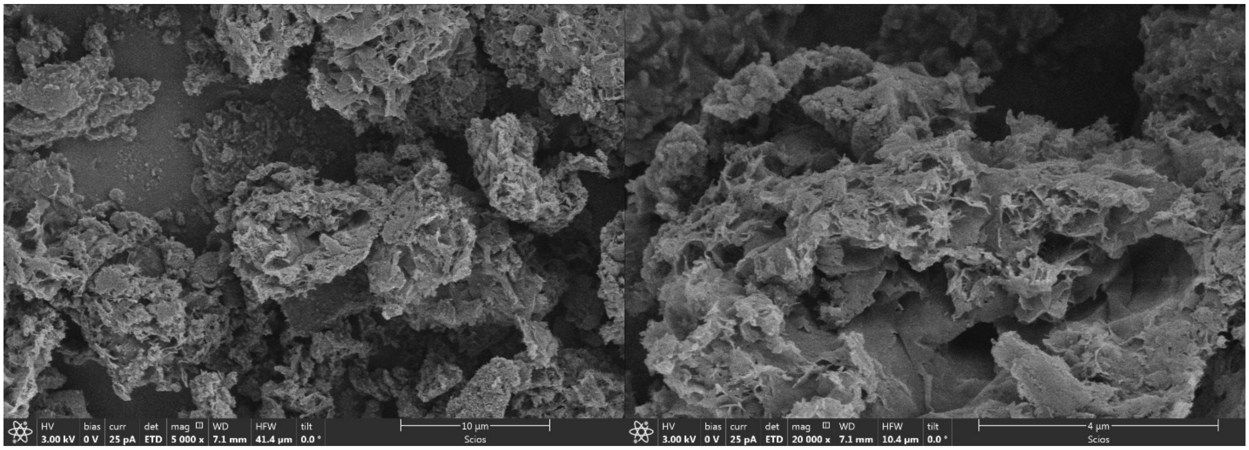

Figure S191. SEM micrographs of polychalcone **PCH-7**.

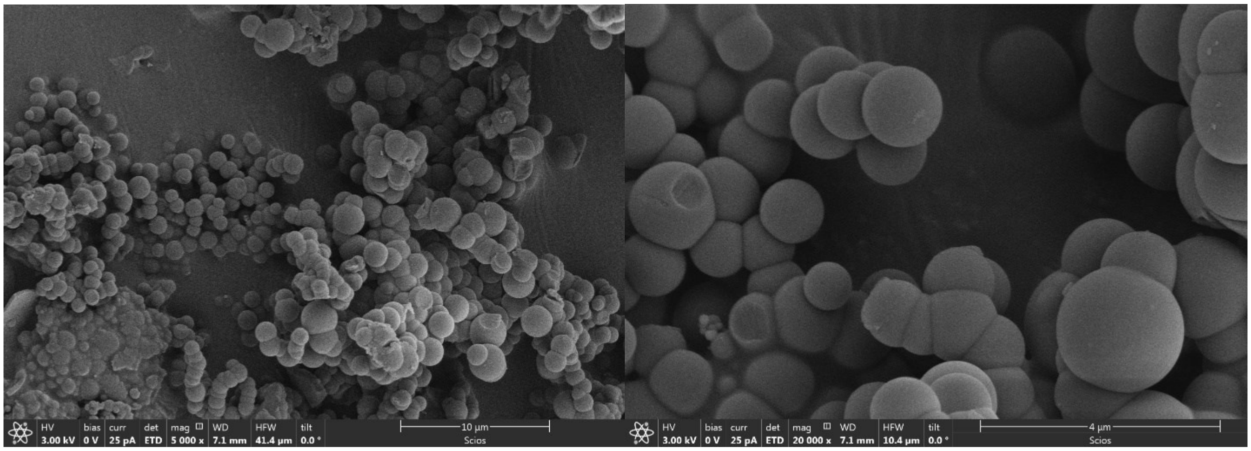

Figure S192. SEM micrographs of polyketone **PAAK-1** obtained in atmosphere of hydrogen.

1.19 Powder XRD and Scanning Electron Microscopy analysis

Table S4. Comments on the analysis of the obtained polymers with SEM and pXRD

Entry	Polymer	SEM	Powder XRD	
			Crystalline state	d-spacing of the largest amorphous bump
1	**PAAK-1**	Spherical agglomerates (size ~1.5 µm)	Amorphous phase clearly formed; size < 2 nm	4.94 Å
2	**PAAK-2**	Spherical agglomerates (size ~0.5 µm)	Amorphous phase clearly formed; size < 2 nm	4.90 Å
3	**PAAK-3**	Inhomogeneous agglomerates with spherical particles (size ~3 µm)	Amorphous phase clearly formed; size < 2 nm	5.19 Å
4	**PAAK-4**	Spherical agglomerates (0.2-2 µm)	Amorphous phase clearly formed; size < 2 nm	4.51 Å
5	**PAAK-5**	Spherical agglomerates (0.1-2 µm)	Amorphous phase clearly formed; size < 2 nm	4.90 Å
6	**PAAK-6**	Inhomogeneous agglomerates with spherical particles (0.1-1 µm)	Amorphous phase clearly formed; size < 2 nm	5.02 Å
7	**PAAK-7**	Spherical agglomerates (size ~2 µm)	Amorphous phase clearly formed; size < 2 nm	4.88 Å
8	**PAAK-8**	Spherical agglomerates (size ~1 µm)	Amorphous phase clearly formed; size < 2 nm	5.18 Å
9	**PAAK-9**	Inhomogeneous agglomerates with spherical particles	Amorphous phase clearly formed; size < 2 nm	5.01 Å
10	**PAAK-10**	Complex inhomogeneous agglomerates With incorporated spherical agglomerates (size ~2 µm)	Amorphous phase clearly formed; size < 2 nm	4.52 Å
11	**PAAK-11**	Complex inhomogeneous agglomerates	Amorphous phase clearly formed; size < 2 nm	4.52 Å
12	**PAAK-12**	Complex inhomogeneous agglomerates	Amorphous phase clearly formed; size < 2 nm	4.97 Å
13	**PCH-1**	Flakes-like crystallites	Semicrystalline material with large unit cell. Peaks are very broad so not possible to index.	
14	**PAAK-1** obtained in H_2	Spherical agglomerates (size ~0.7–2.1 µm)	Amorphous phase clearly formed; size < 2 nm	4.84 Å

2. Mechanical properties

PAAK-1 was prepared according to the method described in Table 2, entry 1 (main paper). The commercial sample of PEEK (450g rod, 300 mm length and 6mm diameter) studied here was purchased from the RS (https://uk.rs-online.com/web/). The commercial HDPE sample (granule, 2-4 mm particle, density: 0.930 g/cm^3) studied here was purchased from the Goodfellow (https://www.goodfellow.com/).

Processing of PAAK-1: The polymer sample was immersed in liquid nitrogen for 30 minutes and pulverised. The polymer was then immersed in THF solvent overnight. Subsequently, the polymer was subjected to vacuum oven drying at 60°C for one hour to achieve complete solvent removal. The dried polymer powder was carefully sandwiched between the bottom and top plates of the mould, ensuring proper alignment. The assembled mould was then transferred to the heated surface of the Specac constant film maker (set to 300 ºC). A constant pressure of 1900 N was applied to the mould for a minimum of one minute to induce the film formation. Upon completion of the film formation process, the mould was removed from the heating surface and allowed to cool completely. Once the mould had cooled, the film was carefully peeled off from the plates. The mould was specifically designed to create a cavity between the top and bottom plates, enabling the formation of a 2 mm film of uniform thickness.

Similarly, HDPE granules and PEEK samples were employed to fabricate 2 mm thick films through compression at the respective temperatures.

Measurement of mechanical properties: The mechanical properties were estimated using nanoindentation method. Nanoindentation techniques are widely employed to assess the mechanical properties of both materials and thin films. It enables the direct measurement of load applied to a sharp indenter and its displacement as a function of indentation depth. The values of hardness and elastic modulus can be extracted from the load-displacement curves obtained during loading and unloading cycles. All three films were individually mounted on a flat magnetic surface of the Nanoindenter. All measurements were conducted using a Nanoindenter Optics11 Chiaro instrument, employing a continuous stiffness method at a frequency of 25 Hz and a suitable displacement amplitude. A spherical indenter with a diameter of 18 µm and a stiffness of 39.53 N/m was utilised. Prior to the measurements, the equipment was calibrated using spherical indenter. Load-displacement curves were analysed using the Oliver and Pharr method [2] to extract mechanical properties. The elastic modulus was calculated based to the following relation. Further changes in equations are used as discussed in [3].

$$S = 2\beta E\sqrt{\frac{A}{\pi}}$$

where A, E, and S represent the contact area, elastic modulus, and contact stiffness, respectively. The geometric correction factor b was assumed to be unity [4]. Given the direct proportionality between the contact area and the square of the contact depth, its value was estimated through calibration using known polymer properties. The proportionality constant provided both the contact area and contact depth values in accordance with the methodology proposed in [5]. The contact stiffness value was determined by measuring the initial slope of the unloading curve, as depicted in **Figure S193** and **Figure S194**. The computed elastic moduli for each polymer are depicted in **Figure S195**. Additionally, the Vickers hardness (HV) values for each polymer were measured using a Mitutoyo series 810 HM 210/220 instrument and reported in **Figure S196**. The obtained values are consistent with previously reported values [6–9].

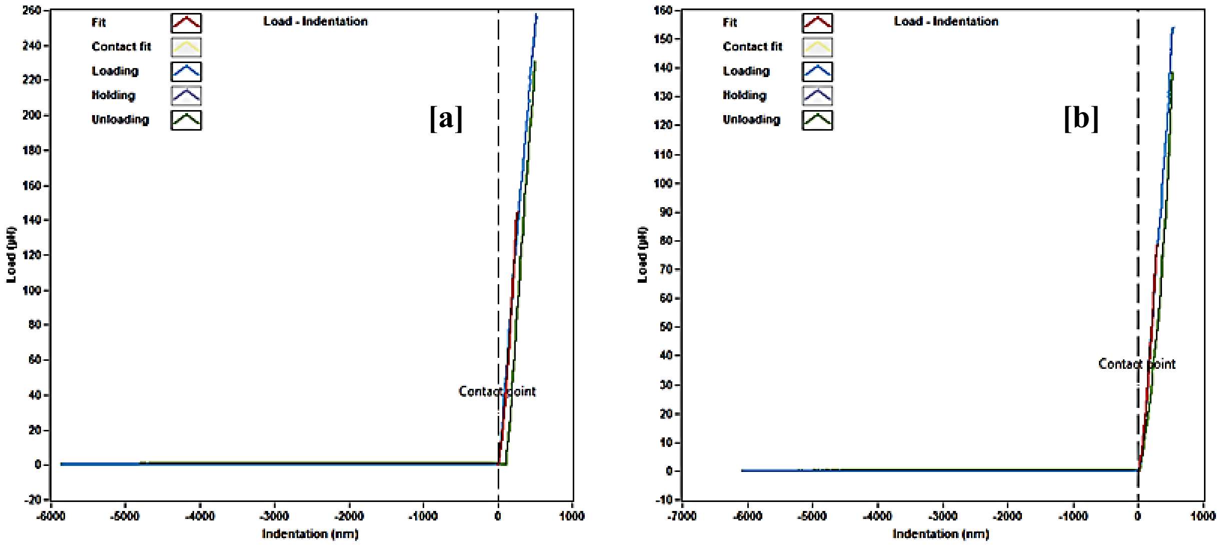

Figure S193. Loading and unloading curves for [a] PEEK and [b] HDPE.

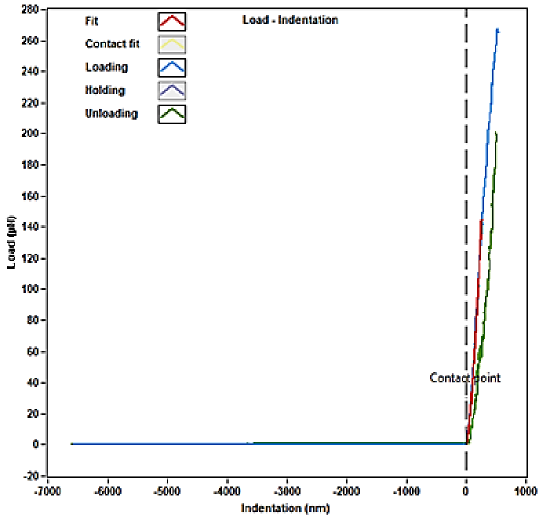

Figure S194. Loading and unloading curves for Polyketone **PAAK-1**.

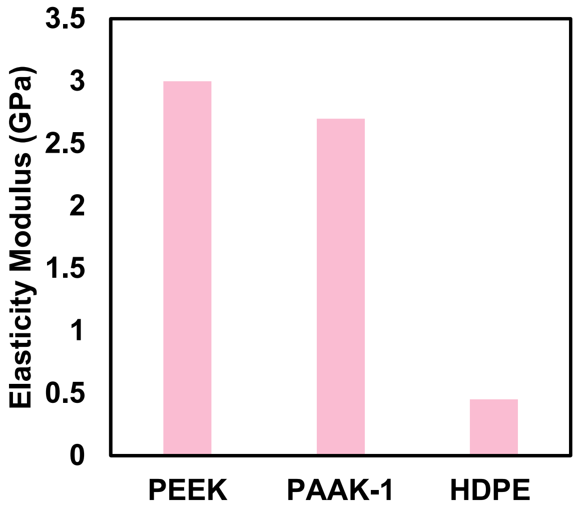

Figure S195. Elastisity modulus of polymers PEEK, PAAK-1 and HDPE.

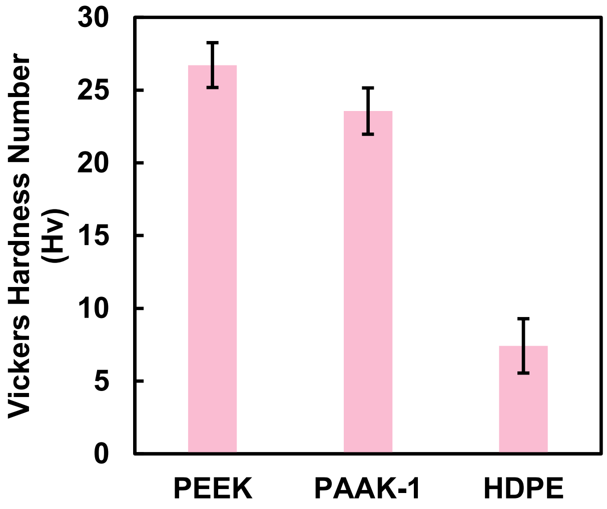

Figure S196. Vickers hardness number of polymers PEEK, PAAK-1 and HDPE.

3. References

[1] Peña-López, M.; Piehl, P.; Elangovan, S.; Neumann, H.; Beller, M. "Manganese-Catalyzed Hydrogen-Autotransfer C−C Bond Formation: α-Alkylation of Ketones with Primary Alcohols." *Angew. Chem. Int. Ed.* **2016**, 55, 14967-14971.

[2] Liu, J.; Hu, K.-F.; Qu, J.-P; Kang, Y.-B. "Organopromoted Selectivity-Switchable Synthesis of Polyketones" *Org. Lett.* **2017** 19 (20), 5593-5596.

[3] Carl, O. C W.; Pharr G. M. "An improved technique for determining hardness and elastic modulus using load and displacement sensing indentation experiments." *Journal of materials research* **1992**, 7(6), 1564-1583.

[4] Fischer-Cripps, A. C. "Examples of nanoindentation testing." *Nanoindentation* **2002**, 159.

[5] Hochstetter, G.; Jimenez, A; Loubet, J. L. "Strain-rate effects on hardness of glassy polymers in the nanoscale range. Comparison between quasi-static and continuous stiffness measurements. *J. Macromol. Sci. Phys.* **1999**, 38(5-6), 681-692.

[6] Chengzhu, L.; Li, Y.; Tjong, S. C. "Polyetheretherketone and its composites for bone replacement and regeneration." *Polymers* **2020**, 12 (12), 2858.

[7] Yutao, Y.; Jiang, C.; Huo, Y.; Li C. "Preparation and tribological behaviors of lubrication-enhanced PEEK composites." *Applied Sciences* **2020**, 10 (21), 7536.

[8] Tebeta, R. T.; Fattahi, A. M.; Ahmed N. A. "Experimental and numerical study on HDPE/SWCNT nanocomposite elastic properties considering the processing techniques effect." *Microsystem Technologies* 2020, 26, 2423-2441.

[9] Nectarios, V.; Petousis M.; Maniadi A. "Sustainable Additive Manufacturing: Mechanical Response of High-Density Polyethylene over Multiple Recycling Processes." *Recycling* **2021**, 6(1), 4.
